# Assessing the efficacy of varicocelectomy, antioxidants, FSH treatment, and lifestyle modifications on sperm DNA fragmentation: a systematic review and meta-analysis

**DOI:** 10.1038/s41598-025-93267-z

**Published:** 2025-03-24

**Authors:** Anett Szabó, Szilárd Váncsa, Péter Hegyi, Tamás Kói, Júlia Ács, Réka Juhász Hermánné, Nándor Ács, Tibor Szarvas, Péter Nyirády, Zsolt Kopa

**Affiliations:** 1 Department of Urology, Semmelweis University, Üllői út 78/b, H-1082 Budapest, Hungary; 2 Centre for Translational Medicine, Semmelweis University, Budapest, Hungary; 3 Institute for Translational Medicine, Medical School, University of Pécs, Pécs, Hungary; 4 Institute of Pancreatic Diseases, Semmelweis University, Budapest, Hungary; 5 Department of Stochastics, Institute of Mathematics, Budapest University of Technology and Economics, Budapest, Hungary; 6 Department of Dietetics and Nutrition Sciences, Semmelweis University, Budapest, Hungary; 7 Department of Obstetrics and Gynecology, Semmelweis University, Budapest, Hungary; 8 Department of Urology, University of Duisburg-Essen and German Cancer Consortium, Essen, Germany

**Keywords:** DNA fragmentation index, DFI, Male fertility, Subfertility, Varicocele repair, Sperm quality, Biomarkers, Endocrinology, Health care, Medical research, Urology

## Abstract

Infertility affects 15% of couples in developed countries, 50% accounting for male origin. In 2021, sperm DNA fragmentation (SDF) testing was incorporated into male fertility evaluations as an evidence-based functional test. We aimed to assess the effect of interventions that could potentially improve SDF. A random-effect meta-analysis was performed with a systematic search in three databases. Pooled mean differences (MD) with 95% confidence intervals (CI) were calculated. Initially, 36,531 articles were found, of which 86 papers with over 8,000 patients were included. Three months following varicocelectomy, the decrease in SDF was -6.74% (CI: -9.40, -4.08) compared to preoperative data of patients, whereas it was -12.39% (CI: -22.41, -2.36) after six months and -10.06% (CI: -22.69, + 2.56) at twelve months. A dose-dependent effect could also be observed in grade II and III varicoceles. The overall SDF decrease at three months was -4.27% (CI: -6.11, -2.43) for antioxidants, -4.51% (CI: -6.81, -2.20) for combined antioxidant therapy and -3.36% (CI: -4.44, -2.28) for monotherapy. In terms of follicle-stimulating hormone treatment (FSH) at three months, the change in SDF was -6.66% (CI: -9.64, -3.69). For lifestyle modifications, a change of -3.24% (CI: -5.33, -1.16) was observed at three months. Other interventions are narratively summarized in the systematic review. Of the interventions reviewed, varicocelectomy is the most effective in reducing SDF at six months, FSH treatment might be useful, antioxidants appear questionable, and lifestyle interventions require more studies of similar designs to draw firm conclusions.

**Supplementary Information:**

The online version contains supplementary material available at 10.1038/s41598-025-93267-z.

## Introduction

In 2020, 50-70 million couples worldwide were affected by infertility, with a gradual increase over the past decades^1–3^. Despite popular misconceptions, male factor infertility is as common as female. Thus, current guidelines suggest that the identification of male causes should be started simultaneously with a diagnostic workup of the female partner^4^.

Besides conventional methods for assessing male infertility, a functional test of sperm DNA fragmentation (SDF) was included in the European Association of Urology (EAU) guidelines in 2021 as the only evidence-based test available. There is still a lack of definitive criteria to differentiate fertile from infertile, and there is no gold standard assay for SDF measurement. However, an optimal SDF is generally considered to be below 25%^5^. The most commonly used assays include sperm chromatin dispersion (SCD), sperm chromatin structure assay (SCSA), Comet, and terminal deoxynucleotidyl transferase dUTP nick end labeling (TUNEL) assays^6^.

Despite these gaps in our knowledge, it is obvious that a more fragmented DNA could have a negative effect on time-to-pregnancy, in vitro fertilization (IVF), and pregnancy outcomes, as well as on the offspring itself; thus, potential risk factors influencing SDF should be identified and targeted to minimise their effects^6^. In our most recent meta-analysis of over 200 risk factors, we found that the presence of varicocele, impaired glucose tolerance, testicular tumors, smoking pollution, and paternal age above 50 years had the most harmful effects on SDF. During pregnancy planning, the effects of non-modifiable risk factors such as age or pollution cannot be eliminated, yet others such as the presence of varicocele, smoking, obesity, and many others can be individually targeted by medications, surgical treatments, or lifestyle modifications. However, SDF-lowering interventions were not examined at that time^7^.

Several articles have summarised the most commonly studied interventions, which are varicocelectomy and antioxidant treatment, of which the former yielded positive results almost without exception when appropriate indications for surgery were applied^8–13^. In contrast, the latter had ambiguous results varying greatly on the exact type of substance used and the measurement method. A meta-analysis by Noegroho et al. summarising the effect of antioxidant treatments based on nine articles found that four studies showed statistically significant reductions in SDF, whereas the other five did not^10^.

Therefore, we aimed to conduct a comprehensive meta-analysis and systematic review to summarise the effects of all interventions studied to date that had the potential to reduce SDF.

## Results

### Search and selection

**Fig. 1:**
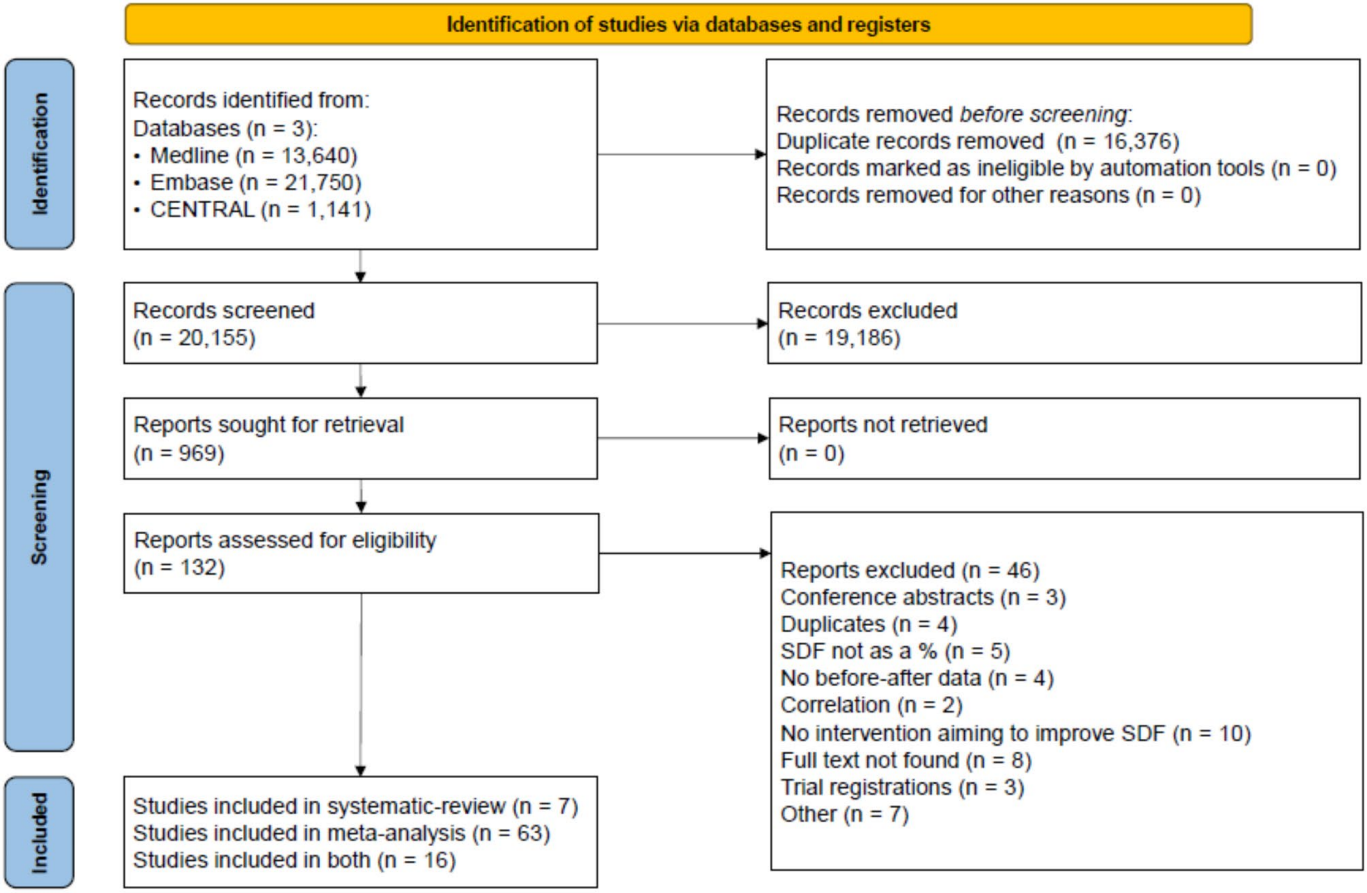
PRISMA flowchart.

Our search key yielded 36,531 articles, of which 86 were suitable for the systematic review or meta-analysis (Fig. 1).

### Basic characteristics of included studies and summary of results

The baseline data of the included studies are detailed in Supplementary Table 2. The earliest study was from 2005, and the latest from 2022, mainly from Asia and Europe. Single-arm studies were the most common, followed by non-randomized studies, and RCTs were the least common. The majority included infertile patients, with the most common interventions being varicocelectomy, antioxidant treatment, follicle-stimulating hormone (FSH) treatment, and lifestyle-based changes. The most common measurement of SDF was performed via TUNEL assays, followed by SCSA, and then SCD.

We summarised the eligibility criteria of each original article in Supplementary Tables 3, and details of the applied interventions and definitions of fertility statuses were provided in Supplementary Table 4.

### Interventions – varicocelectomy

The effect of varicocelectomy was studied in 27 papers with a total of 1,818 men involved. Subgroup analyses were performed based on measurement time after the intervention, assays used for measurement, fertility status of population used as a control and grade of varicocele.

**Fig. 2:**
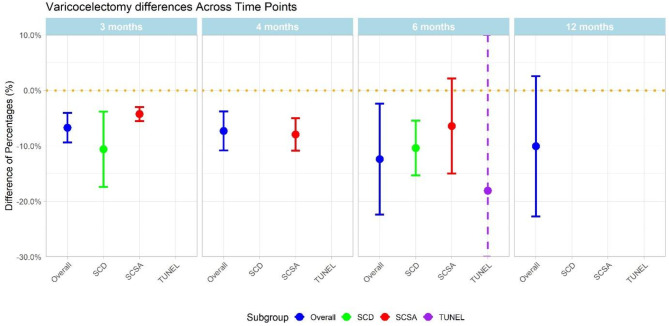
Pooled effect of varicocelectomy on sperm DNA fragmentation with 95% confidence intervals at three, four, six and twelve months pooling results of all assays, SCD, SCSA and TUNEL.

The effect of varicocelectomy was assessed at three, four, six and twelve months following surgery. (Fig. 2) The greatest change compared to pre-operative values was observed at six months with an SDF decrease of -12.39% (CI: -22.41, -2.36) for all assays. When baseline data were compared to fertile controls, the difference in SDF was 14.70% (CI: +8.09, + 21.30), compared to 7.34% (CI: -8.68, + 23.37) after surgery.

On the basis of severity of varicocele, the reduction in SDF following surgery for grade II varicoceles was -4.55% (CI: -5.87, -3.22), compared to -7.35% (CI: -9.28, -5.43) for grade III.

Articles including varicocelectomy that could not be grouped into the above categories or that contained further details as subgroups within the article are summarised in Supplementary Figs. 1–14, 49, and 52–67.

### Interventions – antioxidants

The effect of antioxidants was studied in 39 papers with a total of 4,958 men involved. Subgroup analyses were performed based on measurement time following antioxidant supplementation, assays used for measurement, fertility status of control population and the use of the antioxidants in combination or as a single substance.

**Fig. 3:**
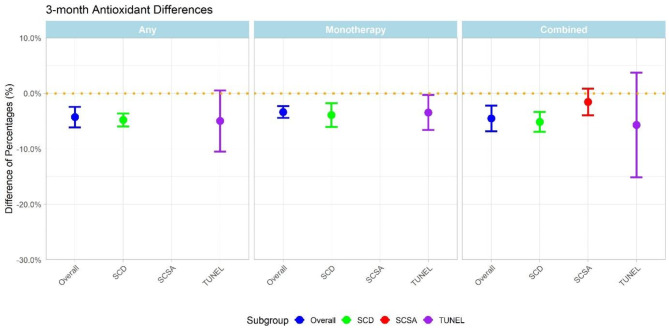
Pooled effect of antioxidant treatment with 95% confidence intervals after three months of mono-, combined or any therapy pooling the results of all assays, SCD, SCSA and TUNEL.

At three months, the change in SDF was similar regardless of merged data of combined antioxidants and monotherapy (-4.27%, CI: -6.11, -2.43), combined antioxidants only (-4.51%, CI: -6.81, -2.20) or monotherapy only (-3.36%, CI: -4.44, -2.28). (Fig. 3).

Looking at RCTs only did not have a significant effect on these results either. (Suplementary Figs. 34–44).

Supplementary Figs. 15–44, 50, and 68–95 summarise articles that included antioxidant treatments that could not be grouped into the above categories or that contained further details as subgroups within the article.

### Interventions – follicle stimulating hormone

The effect of FSH was studied in 8 papers with a total of 637 men involved. Subgroup analyses were performed based on assays used for measurement and FSH dosage.

**Fig. 4:**
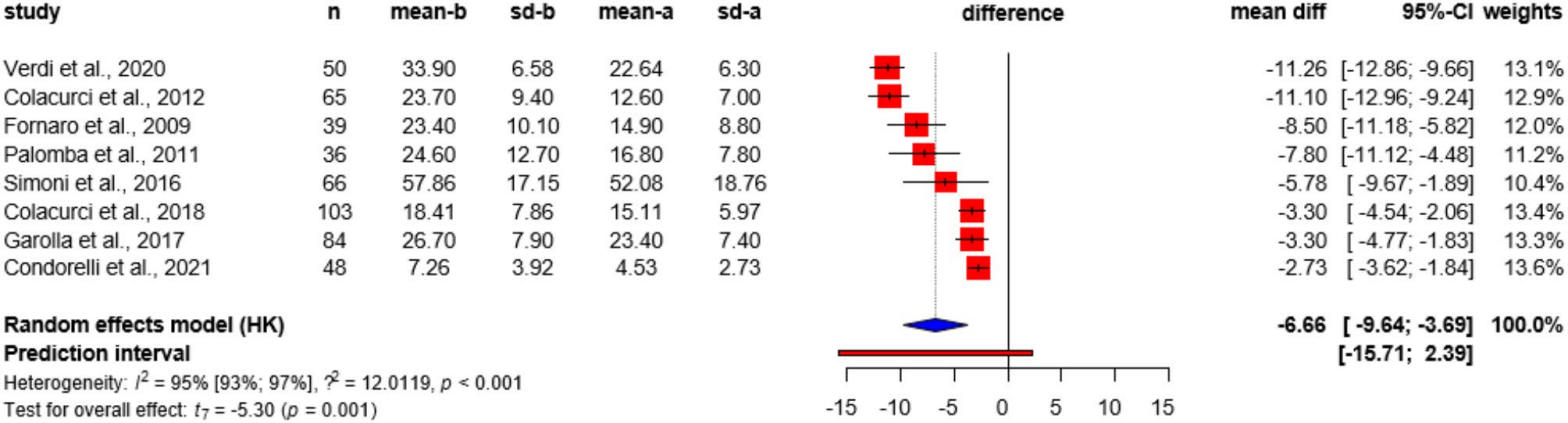
Comparison of mean difference in sperm DNA fragmentation values of patients before FSH treatment and 3 months after FSH treatment, with an input correlation of 0.6.

At three months following treatment, SDF decreased by -6.66% (CI: -9.64, -3.69) compared to baseline data of the same population. (Fig. 4).

Supplementary Figs. 45–47, 51, and 96–103 summarise publications containing FSH treatments that could not be grouped into the above categories or that contained further details as subgroups within the article.

### Interventions – lifestyle changes

The effect of lifestyle interventions was studied in 7 papers with a total of 587 men involved. Subgroup analyses were based on measurement time post-intervention.

After lifestyle changes mainly related to exercise, the reduction in SDF compared to baseline values was -2.94% (CI: -4.94, -0.95), with a difference of -3.24% (CI: -5.33, -1.16) when measured at three months.

Lifestyle interventions are summarised in Supplementary Fig. 48, and 104–105.

### Interventions – other interventions

**Fig. 5:**
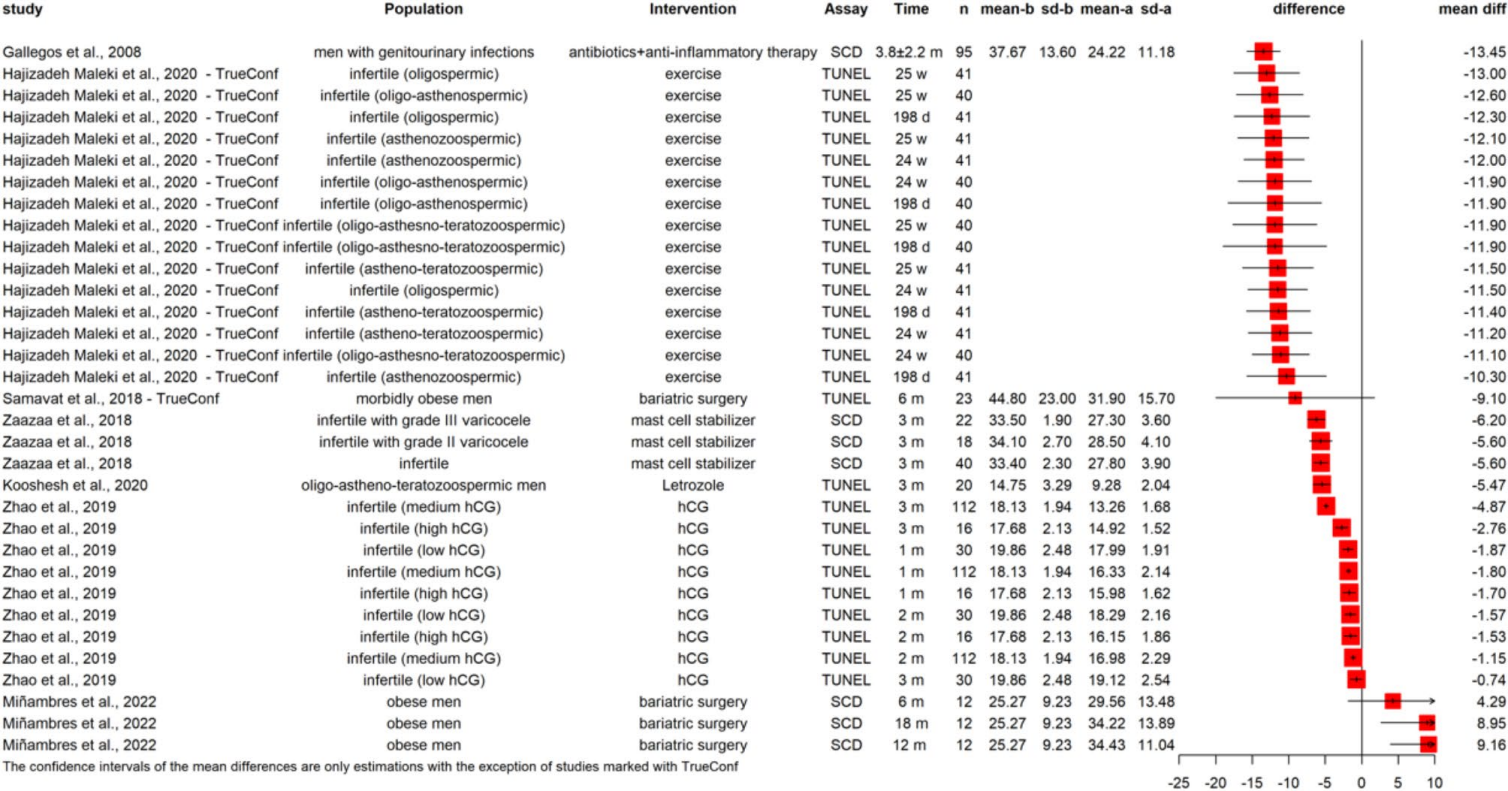
Summary figure of interventions not categorised otherwise with a 0.6 input correlation and 95% CI (n: number of men involved, m: months, w: weeks, d: days, hCG: human chorionic gonadotropin, TrueConf: true confidence intervals defined by the original authors).

Figure 5 includes interventions that were examined in only one or two articles and those that could not be grouped together. Of these interventions, the greatest improvement was achieved in men with genitourinary infections treated with a combination of antibiotics and anti-inflammatory drugs (MD: -13.45%).

Subgroup analyses based on SDF assays used and fertility status of men included are also summarised in the Supplementary Material.

### Risk of bias and grade assessment

Of the 46 single-arm studies, most had a low risk of bias. The 19 two-arm studies were downgraded mainly because they did not take confounding factors into account, whereas the 21 RCTs included had mainly a low risk of bias. (Supplementary Tables 5–7).

Grade-pro was performed separately for the four main interventions. Low certainty rating was received for varicocelectomy, and very low for the other interventions. (Supplementary Table 8).

### Publication bias and heterogeneity

Publication bias was not assessed as standard errors of the mean differences were only estimates derived from inputed correlation coefficients.

Heterogeneity was high for almost all interventions, most probably due to differences in the implementation of interventions, high variability in baseline data of populations included, the use of multiple assays, difficulty in reproducing measurements and natural variability in sperm parameters in any men.

## Discussion

In our meta-analysis, we summarised the effect of all interventions that had been studied in relation to SDF, with a focus on varicocelectomy, antioxidant supplementation, FSH treatment, and lifestyle modifications.

Our study showed a clinically relevant reduction in SDF in men mainly attending assisted reproductive centres, following varicocelectomy, concordant with previous findings. Moreover, a dose-dependency was also observed when considering the severity of varicocele for grade II or III, and the greatest improvement in SDF was observed at six months, with a slight decrease at twelve months. The beneficial effect of varicocelectomy on SDF values has been extensively studied with several meta-analyses supporting its positive effects on fertility parameters when the presence of varicocele is identified as a cause for infertility^8,11,14,15^. The primary underlying cause, amongst several other theories, is increased scrotal temperature due to increased blood flow and decreased drainage, creating unfavourable conditions for testicular function through increased formation of reactive oxygen species^16^.

In a recent meta-analysis by Neto et al. that included patients with clinical varicocele, the overall reduction in SDF following varicocelectomy was -7.23% (CI: -8.86 to -5.59) compared to preoperative data, with a greater change in the subgroup of patients with an initial SDF of over 20%. Varicocelectomy was effective regardless of surgical technique – microsurgical or non-microsurgical^14^.

Such firm results support the association between varicocelectomy and improved fertility, with research in this field slightly subsiding after a peak in 2021, following the inclusion of SDF measurements in the EAU Guidelines the year before^17^.

On the other hand, the relationship between antioxidant treatments and SDF changes are still being extensively studied in depth as findings are more diverse and their beneficial effects are not yet clear.

In our study, regardless of antioxidant mono- or combined therapy and measurement time, their effects were not clinically relevant, though a statistically significant improvement was observed in all cases, and when compared to other populations, treated infertile men had better SDF outcomes than untreated ones; however, they were still inferior to fertile individuals even after treatment.

The challenge with the use of antioxidants is that the balance between antioxidant and pro-oxidant levels within a person is not known; thus, individuals may experience both oxidative or reductive stress; moreover, prolonged use of antioxidants has been associated with a paradoxical increase in oxidative stress^18–22^. A wide variety of assays are available to measure oxidative stress, depending on the sample type. The drawback is their static, rather than dynamic measurement of the indirect effects of oxidative stress – damage caused by reactive oxygen species, mainly to nucleic acids, lipids or proteins. Furthermore, these measurements are only applicable to the sample and may not be true for the entire organ; thus, their applicability to complex species such as humans is highly questionable^23^. Not surprisingly, in a recent meta-analysis by Noegroho et al., on antioxidant supplementations to improve SDF, despite a statistically significant improvement, no clinically relevant change could be observed following treatment, consistent with data from our meta-analysis^10^.

FSH treatment appears to be a more promising intervention. It was suggested over a decade ago that it may be beneficial in fertility treatments even in cases other than hypogonadotropic hypogonadism, in which condition its effects are undoubted. FSH has several roles in spermatogenesis, including the stimulation of androgen receptor expression, thus increasing the susceptibility of Sertoli cells to endogenous testosterone^24^. Without testosterone, spermatogenesis is incomplete and is arrested at meiosis^25^.

In our study, there was a greater improvement with FSH than with antioxidants, though the level of clinical relevance was not reached either. The population of included studies consisted of infertile men, not specifically those with hypogonadotropic hypogonadism. For FSH treatment, as well as for all other interventions, it is true that results should be interpreted with caution because of high heterogeneity rooting in differences in interventions, included populations and the vast number of potential confounding factors which cannot be eliminated.

Of the four main interventions, lifestyle modifications differed the most, including only exercise-based interventions, as well as exercise-based interventions supplemented with antioxidants, and also, dietary modifications. Therefore, no firm conclusions could be drawn, though slight statistical improvements were seen.

On the basis of our previous meta-analysis from 2023, the effect of lifestyle modifications was anticipated to be negligible, as body mass indexes (BMI) had little effect on SDF. Men with a BMI above normal had similar SDF values as those within the normal range (MD = 0.88%, CI: -1.73, + 3.49). Of the possible lifestyle interventions, smoking cessation would have the greatest impact, with smokers having a higher SDF by 9.19% (CI: +4.33, + 14.06), but this intervention was missing from the assessed articles, likely due to the foreseen low adherence of patients^7^.

Nevertheless, the need for robust assessment tools is essential to understanding the true impact of any intervention, and the use of accurate and reproducible assays is therefore crucial. On the basis of of our study, TUNEL assay measurements mostly yielded statistically non-significant results for any intervention; therefore, it appears to be the least reproducible of the four main assays, whereas SCD results seem consistent throughout the studies. In order to clarify the effect of interventions, it would be necessary to standardise measurement techniques and select the most appropriate assay, potentially favouring one, such as SCD, which is known to be easy to perform, bearing in mind that it underestimates fragmentation compared to TUNEL^26^.

### Strengths and limitations

The strengths of our analysis include adhesion to our pre-registered protocol. Also, methodologically, a sensitivity analysis was performed with the use of different correlation coefficients, yielding very similar answers. We covered every intervention that had been studied in connection with SDF and included more than 8,000 men. Subgroup analyses were performed for assays, time of measurement and other relevant data.

Limitations of our study are due to its nature, namely that confounding factors cannot all be accounted for, interventions are not uniform throughout studies, nor are assays used for SDF measurement, as detailed above. Also, few randomized controlled trials were performed due to study designs or ethical reasons. Moreover, SDF is a highly variable biological parameter, and repeated testing alone can lead to apparent changes regardless of intervention. This potential source of bias should be considered when interpreting results.

## Methods

We present our meta-analysis and systematic review based on the Preferred Reporting Items for Systematic Reviews and Meta-Analyses (PRISMA) 2020 guidelines (see Supplementary Table 1) and the Cochrane Handbook^27^. Our study protocol was registered in advance on PROSPERO with the registration number CRD42021283784, and we fully adhered to it.

### Eligibility criteria

We formulated the clinical question using the PICO (Population, Intervention, Comparison, Outcome) framework. Eligible studies included male patients of any fertility status who had undergone an intervention to improve their SDF. These patients were to be compared with another group of males who had no intervention, placebo, or pre-treatment data on SDF. The most commonly applied interventions included surgeries, mainly varicocelectomy, lifestyle modifications, and hormonal or antioxidant treatments. Any method of SDF measurement was accepted (e.g., SCD, SCSA, Comet assay, TUNEL). Eligible studies reported SDF as a percentage or a rate of high SDF with specific cut-off values. However, the latter were later excluded as only a few studies reported data with cut-off values and thus could not be pooled. A difference of 10% was determined clinically relevant, as such a change was considered likely to exceed random variation, although this threshold was established based on our judgement due to lack of consensus.

Randomised controlled trials (RCTs), retrospective and prospective cohort studies with single-arm or double-arm designs were eligible. Studies were not excluded based on language criteria.

Conference abstracts, case series, case reports and reviews were excluded if they had conflicting data, incomplete results, or if data were presented in a way other than percentages.

### Information sources and search strategy

We performed a systematic search in MEDLINE (via PubMed), Embase, and Cochrane Central Register of Controlled Trials (CENTRAL) on October 17, 2021, and then rerun on January 3, 2023. Our systematic search used the following search key: (“sperm DNA fragmentation” OR “SDF” OR “DNA fragmentation index” OR “DFI”). No filters or other restrictions were applied.

### Selection process

For duplicate removal and selection processes, Endnote v9.0 (Clarivate Analytics, Philadelphia, PA, USA) reference manager software was used. Two pairs of independent review authors (RJH-AS, JÁ-SV) performed the selection by title-abstract, then full-text references. Cohen’s kappa coefficient (κ) was calculated afterwards to measure interrater agreement. Disagreements were resolved by two senior authors on each level (TS, NÁ).

### Data collection process and data items

Two authors collected data from the selected full-text articles into a predefined data collection sheet (AS, JÁ). The following data were extracted: first author, publication year, study period and study design, number of participants and demographic data, fertility status, type of intervention, type of control used, type of SDF assay, cut-off values for dichotomous outcomes, mean differences (MD) with their respective distributions for continuous data, and information to assess risk of bias or grade, if applicable.

In case of missing data, the original study investigator was contacted.

Whenever possible, participants in each study were grouped according to their fertility status (fertile, infertile, or general population of unknown fertility status) based on classifications of the original articles.

Studies with only cut-off values rather than MD were excluded during data synthesis.

The preferred reporting of SDF was the mean with corresponding standard deviation (SD). If data were given as mean and standard error (SE), SE was converted to SD. If data were provided as median with range or interquartile range, calculations were made to approximate the mean and SD based on the work by Wan et al.^28,29^ If populations in the original articles were grouped based on anything other than fertility status, or if several similar interventions were implemented within an article, data were merged based on the recommendation of the Cochrane Handbook^27,30^. However, SDF values provided by different assays were handled separately.

### Study risk of bias and grade assessment

Two review authors (PH, SV) performed the risk of bias assessment independently using the Methodological Index for Non-Randomized Studies (MINORS) tool for single-arm studies, the Risk Of Bias In Non-randomised Studies – of Interventions (ROBINS-I) for two-arm studies, and the Risk of Bias 2 (RoB2) for RCTs. Grade assessment was performed using the Grading of Recommendations Assessment, Development and Evaluation (GRADEpro) tool for RCTs.

Assessment categories were predetermined for each tool (Supplementary Appendix). Any disagreements were resolved by a third review author (PN).

### Synthesis methods

We utilized the statistical software R (version 4.1.2.) for conducting the statistical analyses. The meta-analysis adhered to the guidance provided by Harrer et al.^31,32^.

We meta-analysed the pre- and post-treatment SDF means and their difference within the experimental group. Fertile or infertile control results were also available in several cases. Some of the studies measured control patients at the same time as the intervention before values were measured; some performed the measurement later, and some measured the SDF value at several time points. Due to the lack of intervention, we only expected random changes in these control groups. For this reason, we used only one available control SDF mean in each study without distinguishing time points. We meta-analysed the difference of the resulting control means from before and after mean results in the intervention group. In all meta-analyses, we employed the classical inverse variance random-effects method with the restricted maximum likelihood estimator and Hartung-Knapp adjustment. Alongside the prediction interval, heterogeneity was assessed by calculating the I² measure and its confidence interval and by performing the Cochrane Q test. To gain a deeper understanding of heterogeneity, we performed the leave-one-out method described by Harrer et al. as a supplementary/sensitivity analysis^31^.

In almost all cases, standard deviations of the pre- and post-treatment outcomes were available or could be estimated, but the standard deviation of the change was missing. Following the instructions of the Cochrane Handbook, we inputted several different correlations^27^. All correlations used provided similar results. Published results were obtained with an input correlation of 0.6. It should be emphasised that although the pooled outcome and its CI were roughly the same in all cases, the confidence intervals of the individual study results naturally depended on the inputed correlation.

For the most important outcomes, when approximately 10 studies were involved in the analysis, publication bias in the form of a small study effect was assessed by creating a funnel plot and performing Egger’s test.

## Conclusion

Our results further support the potential benefits of varicocelectomy. In contrast, the effectiveness of lifestyle modifications, antioxidants and FSH treatment remains uncertain due to high heterogeneity, although FSH treatment appears to be the most promising among these interventions.

### Implications for practice and research

The evidence to date suggests that there are clear benefits to be gained from the early translation of research findings into clinical practice^33,34^. Our findings suggest a comprehensive approach to improving male fertility, considering both medical- and lifestyle factors. Patients seeking improvement should not only rely on medical professionals but also be encouraged to contribute to their well-being by optimizing their lifestyles.

From a medical perspective, optimal SDF is achieved by collecting and using sperm six months post-varicocelectomy. Patients with grade III varicoceles are likely to benefit most from the surgery. FSH could also be given for slight improvement, but antioxidants do not seem beneficial.

The most pressing issue for research is the lack of standardisation of assays, as well as a gold standard measurement method. For the extensively studied antioxidants, reliable means are also needed to assess reductive and oxidative stress.

## Electronic supplementary material

Below is the link to the electronic supplementary material.

Supplementary Material 1

## Data Availability

The datasets used in this study can be found in the full-text articles included in the systematic review and meta-analysis.
