# Supplementary material for: Assessing the efficacy of varicocelectomy, antioxidants, FSH treatment, and lifestyle modifications on sperm DNA fragmentation: a systematic review and meta-analysis
Source: Sci Rep. 2025 Mar 24;15:10118. doi: 10.1038/s41598-025-93267-z (PMC11933313; doi:10.1038/s41598-025-93267-z)
Supplement: Supplementary file 1 — Supplementary Material 1 [file 41598_2025_93267_MOESM1_ESM.docx]

**Supplementary Material**

**Title**

Assessing the efficacy of varicocelectomy, antioxidants, FSH treatment, and lifestyle modifications on sperm DNA fragmentation: a systematic review and meta-analysis

**Authors**

Anett Szabó^1,2^, Szilárd Váncsa^2,3,4^, Péter Hegyi^2,3,4^, Tamás Kói^2,5^, Júlia Ács^1,2^, Réka Juhász Hermanné^6^, Nándor Ács^2,7^, Tibor Szarvas^1,2,8^, Péter Nyirády^1,2^, Zsolt Kopa^1,2^

**Affiliations:**

1. Department of Urology, Semmelweis University, Budapest, Hungary
2. Centre for Translational Medicine, Semmelweis University, Budapest, Hungary
3. Institute for Translational Medicine, Szentágothai Research Centre, Medical School, University of Pécs, Pécs, Hungary
4. Institute of Pancreatic Diseases, Semmelweis University, Budapest, Hungary
5. Department of Stochastics, Institute of Mathematics, Budapest University of Technology and Economics, Budapest, Hungary
6. Department of Dietetics and Nutrition Sciences, Semmelweis University, Budapest, Hungary
7. Department of Obstetrics and Gynecology, Semmelweis University, Budapest, Hungary
8. Department of Urology, University of Duisburg-Essen and German Cancer Consortium, Essen, Germany

**TABLE OF CONTENT**

**Supplementary Appendix 1.** MINORS risk of bias assessment methodology

**Supplementary Table 1.** PRISMA 2020 checklist

**Supplementary Table 2.** Basic characteristics of the included article

**Supplementary Table 3.** Eligibility criteria in each included article

**Supplementary Table 4.** Intervention and population definitions in each included article

**Supplementary Table 5.** Risk of bias assessment using the ROBINS-I tool

**Supplementary Table 6.** Risk of bias assessment using the MINORS tool

**Supplementary Table 7.** Risk of bias assessment using the RoB2 tool

**Supplementary Table 8.** Grade assessment using grade-pro

**Supplementary Figures 1-14.** Varicocelectomy

**Supplementary Figures 15-44.** Antioxidants

**Supplementary Figures 45-47.** FSH

**Supplementary Figures 48.** Lifestyle interventions

**Supplementary Figures 49-51.** Summary figures

**Supplementary Figures 52-67.** Varicocelectomy (additional figures)

**Supplementary Figures 68-95.** Antioxidants (additional figures)

**Supplementary Figures 96-103.** FSH (additional figures)

**Supplementary Figures 104-105.** Lifestyle interventions (additional figures)

**Supplementary Appendix 1.** MINORS risk of bias assessment methodology

The items are scored 0 (not reported), 1 (reported but inadequate) or 2 (reported and adequate). The global ideal score being 16 for non-comparative studies and 24 for comparative studies. Points 9-12 were not assessed as all evaluated studies were single-arm studies.

**1. A clearly stated aim:** 0 points were given if the aim was not mentioned. 1 point was given if the aim was vaguely stated. 2 points were given if the aim was clearly defined.

**2. Inclusion of consecutive patients:** 0 points were given if it was likely that patients were left out from the study. 1 point was given if some patients might have been left out. 2 points were given if patients were enrolled consecutively.

**3. Prospective collection of data:** 0 points were given for retrospective studies. 1 point was given for longitudinal cohorts. 2 points were given for prospective studies.

**4. Endpoints appropriate to the aim of the study:** 0 points were given if sperm DNA fragmentation was measured via assays others than SCSA, TUNEL, SCD or alkaline Comet assay and the measurement following the intervention was done less than 3 months afterwards. 1 point was given if either the measurement time or assay was not ideal. 2 points were given if both the measurement time and assay was sufficient.

**5. Unbiased assessment of the study endpoint:** 0 points were given if clinicians might have performed the sperm DNA fragmentation measurements themselves. 1 point was given if it was not clearly stated if the assessors of sperm DNA fragmentation were aware of clinical patient data. 2 points were given if it was clear, that assessors of DNA fragmentation were unaware of clinical data.

**6. Follow-up period appropriate to the aim of the study:** 0 points were given if measurement following the interventions were taken less than 3 months afterwards. 1 point was given if the measurement time was not clearly defined (“at least…”) but was more than 3 months after the intervention. 2 points were given if the measurement time was clear and at was at least 3 months following the intervention.

**7. Loss to follow up less than 5%:** 0 points were given for retrospective studies, where loss to follow-up was not applicable. 1 point was given if loss to follow-up was not clear throughout the study or loss was >5%. 2 points were given if loss to follow-up was <5% and patient numbers were clear throughout.

**8. Prospective calculation of the study size:** 0 points were given if sample size calculations were not mentioned. 1 point was given if it was mentioned that the sample size was sufficient. 2 points were given if sample size calculations were performed.

**Supplementary Table 1.** PRISMA 2020 checklist

| **Section and topic** | **Item #** | **Checklist item** | **Location where item is reported** |  |
| --- | --- | --- | --- | --- |
| **Title** | | | | |
| Title | 1 | Identify the report as a systematic review. | Page 1 |  |
| **Abstract** | | | | |
| Abstract | 2 | See the PRISMA 2020 for Abstracts checklist (table 2). | Page 2 |  |
| **Introduction** | | | | |
| Rationale | 3 | Describe the rationale for the review in the context of existing knowledge. | Page 4 |  |
| Objectives | 4 | Provide an explicit statement of the objective(s) or question(s) the review addresses. | Page 5 |  |
| **Methods** | | | | |
| Eligibility criteria | 5 | Specify the inclusion and exclusion criteria for the review and how studies were grouped for the syntheses. | Page 14 |  |
| Information sources | 6 | Specify all databases, registers, websites, organizations, reference lists and other sources searched or consulted to identify studies. Specify the date when each source was last searched or consulted. | Page 15 |  |
| Search strategy | 7 | Present the full search strategies for all databases, registers, and websites, including any filters and limits used. | Page 15 |  |
| Selection process | 8 | Specify the methods used to decide whether a study met the inclusion criteria of the review, including how many reviewers screened each record and each report retrieved, whether they worked independently, and if applicable, details of automation tools used in the process. | Page 15 |  |
| Data collection process | 9 | Specify the methods used to collect data from reports, including how many reviewers collected data from each report, whether they worked independently, any processes for obtaining or confirming data from study investigators, and if applicable, details of automation tools used in the process. | Page 15 |  |
| Data items | 10a | List and define all outcomes for which data were sought. Specify whether all results that were compatible with each outcome domain in each study were sought (e.g., for all measures, time points, analyses), and if not, the methods used to decide which results to collect. | Page 15-16 |  |
|  | 10b | List and define all other variables for which data were sought (e.g., participant and intervention characteristics, funding sources). Describe any assumptions made about any missing or unclear information. | Page 15 |  |
| Study risk of bias assessment | 11 | Specify the methods used to assess risk of bias in the included studies, including details of the tool(s) used, how many reviewers assessed each study and whether they worked independently, and if applicable, details of automation tools used in the process. | Page 16 |  |
| Effect measures | 12 | Specify for each outcome the effect measure(s) (e.g., risk ratio, mean difference) used in the synthesis or presentation of results. | Page 16 |  |
| Synthesis methods | 13a | Describe the processes used to decide which studies were eligible for each synthesis (e.g., tabulating the study intervention characteristics and comparing against the planned groups for each synthesis (item #5)). | Page 15 |  |
|  | 13b | Describe any methods required to prepare the data for presentation or synthesis, such as handling of missing summary statistics, or data conversions. | Page 15 |  |
|  | 13c | Describe any methods used to tabulate or visually display results of individual studies and syntheses. | Page 15 |  |
|  | 13d | Describe any methods used to synthesize results and provide a rationale for the choice(s). If meta-analysis was performed, describe the model(s), method(s) to identify the presence and extent of statistical heterogeneity, and software package(s) used. | Page 16-17 |  |
|  | 13e | Describe any methods used to explore possible causes of heterogeneity among study results (e.g. subgroup analysis, meta-regression). | - |  |
|  | 13f | Describe any sensitivity analyses conducted to assess robustness of the synthesized results. | Page 16 |  |
| Reporting bias assessment | 14 | Describe any methods used to assess risk of bias due to missing results in a synthesis (arising from reporting biases). | - |  |
| Certainty assessment | 15 | Describe any methods used to assess certainty (or confidence) in the body of evidence for an outcome. | Page 16 |  |
| **Results** | | | | |
| Study selection | 16a | Describe the results of the search and selection process, from the number of records identified in the search to the number of studies included in the review, ideally using a flow diagram (see fig 1). | Page 6, Figure 1 |  |
|  | 16b | Cite studies that might appear to meet the inclusion criteria, but which were excluded, and explain why they were excluded. | - |  |
| Study characteristics | 17 | Cite each included study and present its characteristics. | Supplementary Material References, Supplementary Table 2 |  |
| Risk of bias in studies | 18 | Present assessments of risk of bias for each included study. | Page 8, Supplementary Table 8-9 |  |
| Results of individual studies | 19 | For all outcomes, present, for each study: (a) summary statistics for each group (where appropriate) and (b) an effect estimate and its precision (e.g. confidence/credible interval), ideally using structured tables or plots. | Figure 2-5, Supplementary Figure 1-105 |  |
| Results of syntheses | 20a | For each synthesis, briefly summarize the characteristics and risk of bias among contributing studies. | Page 9 |  |
|  | 20b | Present results of all statistical syntheses conducted. If meta-analysis was done, present for each the summary estimate and its precision (e.g. confidence/credible interval) and measures of statistical heterogeneity. If comparing groups, describe the direction of the effect. | Figure 2-5, Supplementary Figure 1-105 |  |
|  | 20c | Present results of all investigations of possible causes of heterogeneity among study results. | Page 9 |  |
|  | 20d | Present results of all sensitivity analyses conducted to assess the robustness of the synthesized results. | - |  |
| Reporting biases | 21 | Present assessments of risk of bias due to missing results (arising from reporting biases) for each synthesis assessed. | - |  |
| Certainty of evidence | 22 | Present assessments of certainty (or confidence) in the body of evidence for each outcome assessed. | Supplementary Figures 52-105 |  |
| **Discussion** | | | | |
| Discussion | 23a | Provide a general interpretation of the results in the context of other evidence. | Page 10-13 |  |
|  | 23b | Discuss any limitations of the evidence included in the review. | Page 13 |  |
|  | 23c | Discuss any limitations of the review processes used. | - |  |
|  | 23d | Discuss implications of the results for practice, policy, and future research. | Page 18 |  |
| **Other information** | | | | |
| Registration and protocol | 24a | Provide registration information for the review, including register name and registration number, or state that the review was not registered. | Page 14 |  |
|  | 24b | Indicate where the review protocol can be accessed, or state that a protocol was not prepared. | Page 14 |  |
|  | 24c | Describe and explain any amendments to information provided at registration or in the protocol. | Page 14 |  |
| Support | 25 | Describe sources of financial or non-financial support for the review, and the role of the funders or sponsors in the review. | Page 21 |  |
| Competing interests | 26 | Declare any competing interests of review authors. | Page 21 |  |
| Availability of data, code, and other materials | 27 | Report which of the following are publicly available and where they can be found: template data collection forms; data extracted from included studies; data used for all analyses; analytic code; any other materials used in the review. | - |  |

**Supplementary Table 2.** Basic characteristics of the included articles

| **Author (year)** | **Study site** | **Study type** | **Population** | **Participants (patients**±**controls)** | **Age (year) ‡** | **Intervention** | **Control** | **DFI measurement** |
| --- | --- | --- | --- | --- | --- | --- | --- | --- |
| Abad (2013) ^1^ | Spain | R | Asthenozoospermic | 20 | ND | Antioxidant | - | SCD |
| Abbasi (2021)^2^ | Iran | R | Infertile | 22+25 | 34±ND | Antioxidant | Infertile – placebo | SCSA |
| Abdelaziz (2015) ^3^ | Egypt | P | Infertile with varicocele | 54 | 27.7±7.2 (21-38) | Varicocelectomy | - | TUNEL |
| Abdelbaki (2017) ^4^ | Egypt | P | Infertile with varicocele | 55+20 | 31 (23-49) | Varicocelectomy | Normozoospermic – no treatment | SCSA |
| Afsin (2018)^5^ | Turkey | R | Varicocele patients | 40 | ND (15-30) | Varicocelectomy | - | TUNEL |
| Alahmar (2020)^6^ | Iraq | R | Infertile | 65+40 | 30±10.5 | Antioxidant | Fertile – no treatment | SCD |
| Alahmar (2021)^7^ | Iraq | R | Infertile | 50+50 | 32±13 | Antioxidant | Fertile – no treatment | SCD |
| Alahmar (2022)^8^ | India | P | Idiopathic oligoasthenozoospermic | 65+58 | 35±14.5 | Antioxidant | Fertile – no treatment | SCD |
| Alhathal (2016)^9^ | Canada | P | Infertile | 29+6 | ND | Varicocelectomy | Healthy control – no treatment | SCSA |
| Arafa (2020)^10^ | Qatar | P | Infertile | 112 | 35.9±0.5 | Antioxidant | - | SCD |
| Baker (2013)^11^ | USA | R | Varicocele patients | 22 | 33.4±ND | Varicocelectomy | - | TUNEL |
| Boeri (2022)^12^ | Italy | P | Infertile | 30+30 | 36 (32-39) | Antioxidant | Antioxidant (no SOD) | SCSA |
| Cannarella (2021)^13^ | Italy | R | Chronic autoimmine thyroiditis patients | 20 | 32.2±7.1 | Antioxidant | - | TUNEL |
| Capece (2017)^14^ | Italy | RCT | Infertile | 28+28 | 28.6±6 | Antioxidant | Placebo | TUNEL |
| Colacurci (2018)^15^ | Italy | P | Infertile | 103 | 36.09±4.67 | FSH | - | TUNEL |
| Colacurci (2012)^16^ | Italy | RCT | Idiopathic OATs patients | 65+64 | 32.6±3.3 | FSH | No treatment | TUNEL |
| Condorelli (2021)^17^ | Italy | R | Infertile | 24+24 | 34.3±5.6 | FSH | Different FSH dose | TUNEL |
| D’argent (2021)^18^ | France | RCT | Infertile | 53+51 | 36.8±6.4 | Antioxidant | Placebo | TUNEL |
| Dadfar (2021)^19^ | Iran | R | Infertile with varicocele | 100 | 33.6±6.41 | Varicocelectomy | - | SCD |
| Dadgar (2022)^20^ | Iran | RCT | Infertile | 31+22 | ND (25-43) | Antioxidant | Placebo | SDFA kit |
| Dhawan (2018)^21^ | India | P | Partners of women with recurrent pregnancy loss | 30 | 33.4±4.7 | Lifestyle | - | SCSA |
| Elsedfy (2018)^22^ | Egypt | R | Beta thalassaemia major zoospermic patients | 10 | 25±3.68 | Antioxidant | - | SCSA |
| Esfahani (2010)^23^ | Iran | R | Infertile with varicocele | 31+52 | ND | Varicocelectomy | Fertile – no treatment | SCD |
| Fathi (2021)^24^ | Egypt | R | Subfertile | 45+40 | 33.4±5.9 | Varicocelectomy | No treatment | SCD |
| Fornaro (2009)^25^ | Italy | R | Infertile | 39 | ND (26-41) | FSH | - | TUNEL |
| Gabriel (2012)^26^ | Canada | P | Infertile | 14 | ND | Varicocelectomy | - | SCSA |
| Gallegos (2008)^27^ | Mexico | P | Men with genitourinary infections | 95+50 | 34.9±5.4 | Antibiotics+anti-inflammatory | Fertile – no treatment | SCD |
| Garolla (2017)^28^ | Italy | R | Infertile | 84+82 | 33.9±5.8 | FSH | Infertile – no treatment | Acridine orange |
| Ghazi (2011)^29^ | Saudi Arabia | P | Infertile | 82 | 35.6±7.2 | Varicocelectomy | - | TUNEL |
| Greco (2005)^30^ | Italy | RCT | Infertile | 32+32 | ND | Antioxidant | Placebo | TUNEL |
| Gual-Frau (2015)^31^ | Spain | R | Infertile | 20 | ND | Antioxidant | - | SCD |
| Habibi (2022)^32^ | Iran | RCT | Infertile | 34+29 | ND | Antioxidant | Placebo | SCSA |
| Hajizadeh Maleki (2020)^33^ | Germany | RCT | Asthenozoospermic infertile | 41+44 | 33±6.6 | Lifestyle | Infertile – no exercise | TUNEL |
| Huang (2020)^34^ | China | RCT | Infertile | 185+185 | 32.3±3.7 | Antioxidant | Placebo | TUNEL |
| Humaidan (2021)^35^ | Denmark | P | Infertile with SDF>15% | 31+10 | 35 [24-55] | Lifestyle | - | SCSA |
| Jannatifar (2020)^36^ | Iran | RCT | Infertile | 50 | ND (25-40) | Antioxidant | - | TUNEL |
| Jannatifar (2021)^37^ | Iran | RCT | Infertile | 50 | ND (25-40) | Antioxidant | - | TUNEL |
| Kavoussi (2021)^38^ | USA | R | Infertile | 141 | 34.3±6.5 | Varicocelectomy | - | SCD |
| Kavoussi (2022)^39^ | USA | R | Infertile | 22 | ND | Varicocelectomy | - | SCD |
| Kooshesh (2020)^40^ | Iran | P | OATs patients | 20 | 34.9±ND | Letrozole | - | TUNEL |
| Kumar (2011)^41^ | India | RCT | Infertile | 21+23 | 31±4 | Antioxidant | Placebo | SCSA |
| La Vignera (2012)^42^ | Italy | R | Infertile with grade 3 varicocele | 30+30 | 26.5±3.2 | Varicocelectomy | Fertile- no treatment | TUNEL |
| La Vignera (2022)^43^ | Italy | R | High risk HPV patients | 25+25 | 32±9.5 | Varicocelectomy | Placebo | TUNEL |
| Lara-Cerrillo (2020)^44^ | Spain | R | Infertile | 20+12 | ND (17-44) | Varicocelectomy | Fertile – no treatment | Alkaline Comet |
| Li (2012)^45^ | Japan | R | Infertile | 19+19 | 33.1±ND | Varicocelectomy | Fertile | SCSA |
| Lipovac (2021)^46^ | Austria | R | Subfertile | 162+177 | ND (18-65) | Lifestyle, antioxidant | Lifestyle only | SCD |
| Maghsoumi-Norouzabad (2021)^47^ | Iran | RCT | Infertile | 43+43 | 34.7±5.3 | Antioxidant | Placebo | SCD |
| Martínez-Soto (2016)^48^ | Spain | RCT | Infertile | 32+25 | 35.3±0.9 | Antioxidant | Placebo | TUNEL |
| Ménézo (2007)^49^ | France | R | Infertile | 57 | ND | Antioxidant | - | SCSA |
| Micic (2019)^50^ | Serbia | RCT | Infertile | 125+50 | 31.5±ND | Antioxidant | Placebo | SCD |
| Miñambres (2022)^51^ | Spain | R | General population | 12 | 45±4.87 | Bariatric surgery | - | SCD |
| Mir (2018)^52^ | India | R | Infertile men with BMI>25 kg/m² | 105 | 32.5±7.5 | Lifestyle | - | SCD |
| Mohammed (2015)^53^ | Egypt | P | Infertile | 75+45 | 31±ND | Varicocelectomy | - | Acridine orange, flow cytometry |
| Moskovtsev (2010)^54^ | Canada | P | Infertile with SDF>30% | 12 | ND | Antioxidant | - | TUNEL |
| Moskovtsev (2009)^55^ | Canada | R | Infertile with SDF>30% | 24 | ND | Antioxidant | - | SCSA |
| Nazari (2020)^56^ | Iran | R | Infertile | 60 | 32.1±5.24 | Antioxidant | - | TUNEL |
| Negri (2017)^57^ | Italy | R | Infertile | 48+15 | 39.4±5.4 | Antioxidant | Infertile – no treatment | SCD |
| Ni (2014)^58^ | China | P | Infertile with varicocele | 42+10 | 30.5±3.6 | Varicocelectomy | Fertile – no treatment | SCSA |
| Ni (2016)^59^ | China | R | Infertile with varicocele | 51+25 | 30±3 | Varicocelectomy | Fertile – no treatment | SCSA |
| Ozer (2020)^60^ | Turkey | R | Infertile with SDF>30% | 163 | 35.31+5.32 | Antioxidant | - | TUNEL |
| Özgök Kangal (2021)^61^ | Turkey | R | Infertile after varicocelectomy | 6 | 35.4±6.9 | Hyperbaric oxygen | - | TUNEL |
| Palomba (2011)^62^ | Italy | P | Infertile | 36 | 34±7.3 | FSH | - | SCD |
| Pelliccione (2011)^63^ | Italy | P | General population | 7 | 33±9 | Lifestyle | - | TUNEL |
| Pini (2020)^64^ | USA | R | Infertile with motility or concentration < WHO lower limit | 35 | 39.51±6.68 | Antioxidant | - | TUNEL |
| Sakamoto (2008)^65^ | Japan | R | Infertile with varicocele | 30 | 32.4±5.6 | Varicocelectomy | - | TUNEL |
| Salas-Huetos (2018)^66^ | Spain | RCT | General population | 49+49 | 24.5±4.6 | Lifestyle | General population – no lifestyle change | TUNEL |
| Salehi (2019)^67^ | Iran | R | Infertile | 485 | ND (20-40) | Antioxidant | - | SCSA |
| Samavat (2018)^68^ | Italy | P | General population with morbid obesity | 23+8 | 39±8 | Bariatric surgery | General population – no surgery | TUNEL |
| Scaruffi (2021)^69^ | Italy | P | Subfertile | 14 | 38.6±4.8 | Antioxidant | - | SCD |
| Simoni (2016)^70^ | Italy | P | Infertile | 28 | 37 (26-48) | FSH | - | TUNEL |
| Smit (2013)^71^ | The Netherlands | P | Infertile | 49 | 34+6.9 | Varicocelectomy | - | SCSA |
| Steiner (2020)^72^ | USA | RCT | Infertile | 73+74 | 34 [30-38] | Antioxidant | Placebo | SCSA |
| Stenqvist (2018)^73^ | Sweden | RCT | Infertile | 37+40 | 37.8±5.1 | Antioxidant | Placebo | SCSA |
| Sun (2018)^74^ | China | RCT | Infertile | 179+179 | 32±5.8 | Varicocelectomy | Infertile – unilateral varicocelectomy | SCSA |
| Telli (2015)^75^ | Turkey | P | Infertile | 72 | 29.3±5.7 | Varicocelectomy | - | Acridine orange |
| Vahidi (2021)^76^ | Iran | P | Infertile | 64 | 34.7±4.66 | Antioxidant | - | TUNEL |
| Verdi (2020)^77^ | Iran | R | Infertile | 50 | ND (25-45) | FSH | - | SCD |
| Wald (2021)^78^ | USA | R | Subfertile | 17 | 35 [27-42] | Varicocelectomy | - | TUNEL |
| Wang (2012)^79^ | China | R | Infertile | 56+30 | 30.2±5.6 | Varicocelectomy | Normozoospemic – no treatment | SCD |
| Werthman (2008)^80^ | USA | R | Infertile | 11 | ND | Varicocelectomy | - | SCSA |
| Yakovlev (2022)^81^ | Russia | RCT | Infertile | 45+45 | ND (25-45) | Antioxidant | Placebo | TUNEL |
| Yuan (2021)^82^ | China | R | Infertile | 22 | ND (28-35) | Antioxidant | - | SCSA |
| Zaazaa (2018)^83^ | Egypt | RCT | Infertile with varicocele | 40 | 27.2±4.2 | Varicocelectomy, mast cell stabilizer | - | SCD |
| Zhang (2022)^84^ | China | R | Infertile | 310+170 | ND | Antioxidant | Placebo | SCSA |
| Zhao (2019)^85^ | China | RCT | Infertile | 30+32 | 32.2±2.8 | hCG | Placebo | TUNEL |
| Zini (2011)^86^ | Canada | P | Infertile | 19 | ND | Varicocelectomy | - | SCSA |

‡ parameters represented as mean±standard deviation, or median with range (minimum and maximum), or median with interquartile range [lower-upper quartile]

SCSA: sperm chromatin structure assay, TUNEL: terminal deoxynucleotidyl transferase (dUTP) nick end labeling, SCD: sperm chromatin dispersion test, SDFA: sperm DNA fragmentation assay, hCG: human choriogonadotropin, FSH: follicle stimulating hormone, P: prospective, R: retrospective, RCT: randomized controlled trial, OAT: oligoasthenoteratozoospermic, BMI: body mass index, WHO: World Health Organization, SOD: superoxide dismutase

ND: not defined

**Supplementary Table 3.** Eligibility criteria in each included article

| **Author (year)** | **Inclusion criteria (“verbatim”)** | **Exclusion criteria (“verbatim”)** |
| --- | --- | --- |
| Abad (2013) ^1^ | At the time the study began, none of the patients had been pre-treated with antioxidants or medicated for anything other condition. | Patients with genitourinary inflammation, leukocytospermia, history of autoimmune disease or altered hormonal profiles were not included in this study. Patients who required treatment during the study for other reasons (mainly influenza) were also excluded. |
| Abbasi (2021)^2^ | According to the World Health Organization’s designated cut-offs, men with sperm concentration < 15 (10˄6/mL) and/or normal morphology < 4 (%) and/or total motility < 40 (%) were considered eligible to participate in the study (solely addressed as oligozoospermia, teratozoospermia, and asthenozoospermia, respectively). | As idiopathic male infertility is a diagnosis of exclusion, men with the following conditions were excluded from the study: cryptorchidism; varicocele; chromosome abnormalities; leukocytospermia; epididymal-orchitis; genitourinary traumas; prostatitis; testicular torsion; history of inguinal/genital surgery; history of hormone therapy; endocrinopathies; history or ongoing use of cytotoxic drugs as well as immunosuppressants, anticonvulsants, and androgens; and recent history of sexually transmitted infections. |
| Abdelaziz (2015) ^3^ | Participating patients did not receive any medical treatment for infertility during the 6 months before enrollment in the study and during the follow‑up period. | Patients with evidence of azoospermia, urogenital infections, hypogonadism, systemic or endocrine disease or a history of smoking, alcohol or drug abuse, hormonal treatment were excluded. |
| Abdelbaki (2017) ^4^ | 60 infertile men complaining of inability to conceive for >12 months of unprotected intercourse and abnormal seminal variables (reduced sperm concentration, motility and morphology on two or more semen samples) associated with unilateral or bilateral clinical varicocele (Grade 1–3). A control group, comprised of 20 normozoospermic healthy fertile men with normal standard semen variables according to WHO criteria | Patients with subclinical varicocele, azoospermia, systemic or endocrine disease, male accessory gland infection, cryptorchidism, testicular atrophy, cigarette smoking, alcohol or drug abuse, or recent hormonal treatment for fertility were excluded from the study. Other exclusion criteria were patients with all other factors that could affect sperm DNA fragmentation (SDF) such as obesity, patients with leucocytospermia or exposed to gonadotoxins, radiochemotherapy, and patients with cancer. |
| Afsin (2018)^5^ | Inclusion of 40 varicocele patients aged between 15 and 30 years. Complaints of patients were infertility, inguinal pain, testicular swelling and prolapse | - |
| Alahmar (2020)^6^ | Patients with infertility of 12 months or more despite regular unprotected intercourse were included in the study. Patients were considered to have OA when their semen analysis showed abnormal sperm concentration (<15 million/mL), progressive motility (<32%), and total motility (<40%) as defined by the WHO 2010 criteria for semen analysis. | Patients with azoospermia, anatomical abnormalities of men genital tract, varicocele, genital infection, scrotal surgery, systemic diseases, smoking, women factor, and recent (within the last six months) antioxidant and selective serotonin reuptake inhibitors intake were excluded from the study. |
| Alahmar (2021)^7^ | - | Men with varicocele; genital infection; azoospermia; anatomical abnormalities; testicular injury or surgery; endocrine, renal, hepatic, or other systemic illnesses; smoking; alcohol intake; and recent antioxidant intake were excluded, as were those taking relevant medications and male partners in couples affected by female factor infertility. |
| Alahmar (2022)^8^ | - | Patients were excluded if they had genital infections, azoospermia, cryptorchidism, varicocele, testicular trauma or surgery, an endocrine disorder, systemic disease, relevant medications, smoking, alcohol, recent administration of antioxidants, and female factor infertility. |
| Alhathal (2016)^9^ | Men presenting to our clinic with one year or more of infertility, a clinically palpable varicocoele and abnormal semen parameters (reduced sperm concentration or motility on two or more semen samples) were deemed to be candidates for varicocoele repair | Men with azoospermia, severe oligozoospermia (<5 million spermatozoa/mL), complete asthenozoospermia, or evidence of genital tract infection were excluded. |
| Arafa (2020)^10^ | Infertile men (between 20 and 50 years of age) with unknown etiology and female infertility factor ruled out were included in the study. | Exclusion criteria included patients with azoospermia or with a sperm concentration <1 million per mL and those with leukocytospermia. In addition, patients having any identifiable cause for infertility such as clinical varicocele (grade 2 and higher), orchitis, epididymitis, cryptorchidism, genetic cause for infertility, or irradiation, as well as subjects who received chemotherapy treatment, clinically meaningful endocrinopathy defined as an endocrinopathy (which requires endocrine medications, for example, diabetes, thyroid disease, pituitary diseases, adrenal diseases, and so on), or abnormal hormonal profile (testosterone <10.4 nmol/L, luteinizing hormone (LH) <1 or > 9 IU/L, and/or follicle stimulating hormone (FSH) <1 or >19 IU/mL, elevated prolactin >407 mIU/L, elevated thyroid-stimulating hormone (TSH) >4.5 U/mL, elevated estrogen >275 pmol/L) were also excluded from the study. Patients receiving antioxidants in the past six months were excluded. Also, patients with dietary or social habits (subjects following any special diet including, but not limited to liquid, high or low protein, raw food, vegetarian, or vegan, among others, as well as consumption of more than one unit of alcohol daily, and history or current use of illegal or “recreational” drugs), as well as medical conditions (such as known HIV infection, malignancy, and renal or hepatic failure) that may impact oxidative stress, were also excluded. |
| Baker (2013)^11^ | - | Exclusion criteria were as follows: patients younger than 18 years, pain as the sole indication for surgery, surgery for recurrent varicoceles, and patients taking concurrent medication for infertility. |
| Boeri (2022)^12^ | Patients were included if they were ≥18 and ≤50 years old and had pure male factor infertility, defined after a comprehensive diagnostic evaluation of all the female partners. SDF ≥ 30% before treatment. | Exclusion criteria were: symptoms suggestive of genitourinary infections; a history of vasectomy, undescended testicle, hypospadias or infertility treatment in the preceding year; and partial or incomplete data concerning one or more of the semen parameters considered. |
| Cannarella (2021)^13^ | Normal BMI | Patients with endocrine (pituitary, adrenal, dysfunction, hypovitaminosis D), metabolic (overweight, obesity, diabetes mellitus), and/or andrological (hypogonadism, varicocele, leukocytospermia, anti-sperm antibodies, male accessory gland infection/inflammation, history of cryptorchidism or urogenital surgery) diseases were ruled out. |
| Capece (2017)^14^ | - | Exclusion criteria were acute and chronic prostatitis, endocrine disorders, systemic disorders, previous penile or testicular infection, microorchidism and cryptorchidism, previous or current varicocele, previous or current hormonal treatment, smokers or ex-smokers. |
| Colacurci (2018)^15^ | Male partner presented the following inclusion criteria, were considered: (i) age between 18 and 45 years, (ii) total sperm number of >10 million/ejaculate, (iii) total sperm motility of >5% and ≤25%, and (iv) FSH value at baseline >1 and <8 IU/L. Thus, patients with both unexplained infertility and idiopathic infertility were included. Considering exclusion criteria, smokers were included in the study, only when the attitude was less than 20 cigarettes per day. | Obvious female infertility causes have been excluded through the anamnesis. Moreover, patients with known genetic causes of male infertility (karyotype abnormalities, and CFTR gene mutations, etc.) have been excluded. Smoking behavior represented a criterion for exclusion only when a current attitude to 20 or more cigarettes/day was discovered or a former attitude of 41 or more cigarettes/day with discontinuation within 6 months before the screening visit. |
| Colacurci (2012)^16^ | Normal hormonal levels. | Each subject underwent a careful anamnesis to exclude systemic diseases, alcohol assumption, and smoking; a careful physical examination and measurement of testicular size with a Prader orchidometer to exclude abnormalities of the external genitalia and cryptorchidism; ultrasonographic examination to exclude varicocele; measurement of serum hormonal levels to exclude abnormal values of FSH (,1, .7 IU/L), luteinizing hormone (LH; ,1, .8 IU/L), and testosterone (T; ,3, .10 ng/mL); microbiological examination and spermioculture to exclude infections; immunobead binding test to exclude the presence of anti-sperm antibodies; karyotype to exclude any chromosomal abnormalities; and genetic examination to exclude Y chromosome microdeletions and cystic fibrosis gene mutations. Only men with sperm count less than 10x10 6 spermatozoa/ mL and forward motility, 25% were included in the study. |
| Condorelli (2021)^17^ | Caucasian men older than 18 years. The following inclusion criteria were also established: sperm concentration > 5 mil/mL, mean testicular volume ranging from 8 to 12 mL, and TT > 350 ng dL−1. | The following exclusion criteria were established for this study: azoospermia, head injury, endocrine disorders (hypogonadism, hyperprolactinemia, Cushing syndrome, acromegaly, hypopituitarism), low or high (>8.0 IU mL−1) serum FSH levels, systemic diseases (kidney, liver, diabetes mellitus, etc.), genetic disorders, male accessory gland infection, and varicocele of high grade (>II). |
| D’argent (2021)^18^ | Only men with at least one abnormal spermatic criterion according to the 2010 reference values of the World Health Organization (WHO) [14] were included in the study. Abnormal spermatic parameters were a sperm concentration <15 million spermatozoa per mL, motility <50% within 60 min of liquefication, and vitality <60% of live spermatozoa. Spermatozoa morphology was considered abnormal when <4% (or <5th centile) of the observed spermatozoa had normal morphology. Only couples with French National Health Insurance, who could speak and write French, with a female partner aged 18 to 38 years old, and without a female factor of infertility were included. None of the men included in the study were on other hormonal or vitamin treatments having a potential impact on fertility. | The exclusion criteria were the presence of a chronic viral disease (hepatitis B, hepatitis C, human immunodeficiency viruses) in either the male or female partner, a medical history of epilepsy, a poorly controlled chronic disease (for example, heart disease, diabetes, hypertension, cancer), prior cancer treatment, permanent obstructive disease of the deferent canal in the male partner, the need for testicular sperm or frozen sperm, supplementation with folic acid before the study, or medical intolerance to folic acid. Couples with a female partner presenting a known etiology of female infertility or ovarian failure defined as a blood follicle stimulating hormone (FSH) level >9 IU/L on day 3 of the menstrual cycle and/or antral follicle count (AFC) < 8 were also excluded from the study. |
| Dadfar (2021)^19^ | - | Exclusion criteria included: previous surgical history, pregnancy history, history of abortion, history of chemotherapy, trauma, and diabetes, radiation and radiography, and other underlying diseases. |
| Dadgar (2022)^20^ | Men aged 25–43 years with idiopathic infertility and no use of contraceptives were the criteria for our study. Any medication that might be affecting the spermatogenesis courses should be discontinued in 3 months to the study. Also, inclusion criteria were a history of infertility for at least 1 year; normal levels of reproductive hormones; and total testicular volume C12 ml. | Men were deprived of the study if they had a history of chemotherapy, androgens, anti-androgens, renal insufficiency, presence of anti-sperm antibodies, karyotypic abnormalities, leukocytospermia, tobacco use, a history of diseases such as testicular torsion, varicocele, orchitis, cryptorchidism, relevant genitourinary infection, sexually transmitted disease, alcohol or substance abuse, and hyper-prolactinemia. |
| Dhawan (2018)^21^ | Only cytogenetically normal cases were included in the study. | Those with a recent history of any febrile episode, infections or any drug intake in the past three months and also deranged ovarian function, biochemical and hormonal profile in the female partners, were excluded. |
| Elsedfy (2018)^22^ | Twenty fully pubertal BTM patients were included consecutively | - |
| Esfahani (2010)^23^ | - | Individuals included in this study did not have a history of systemic illnesses, cryptorchidism, orchitis, epididymitis, urethritis or testicular atrophy. |
| Fathi (2021)^24^ | Eligibility criteria for the study were that patients with at least a 1-year history of male factor subfertility, a clinically palpable varicocele associated with normal standard semen parameters according to the 2010 WHO criteria [10], and a high DFI% of >25%. | Patients with subclinical varicoceles, associated female factor fertility, recurrent varicoceles, cryptorchidism, genital infection, pyospermia, obesity, cigarette smoking habit, alcohol or drug abuse, patients exposed to gonadotoxins, or patients with cancer were excluded from the study. Those with abnormal semen analyses were excluded from the study. |
| Fornaro (2009)^25^ | Inclusion criteria for male partners: sperm count between 1-10x10˄6/ml, normal baseline serum FSH, LH, prolactin, testosterone, and Inhibin B levels; negative history of cryptorchidism, varicocele, urogenital tract infections, chronic systemic diseases, antisperm antibodies, Y-chromosome microdeletions, karyotype anomalies, CFTR gene mutations. Inclusion criteria for female partners: age <37 years, no other fertility factors apart from tubal anomalies. | - |
| Gabriel (2012)^26^ | Men presenting to our clinic with one year or more history of infertility, a clinically palpable varicocele and poor motility (<25 % rapid progressive and <50 % progressive) were recruited. | Men with azoospermia, severe oligozoospermia (<5 million sperm per ml), complete asthenozoospermia or evidence of genital tract infection were excluded. Couples in whom the wife had tubal obstruction or ovulatory failure were not included. |
| Gallegos (2008)^27^ | - | - |
| Garolla (2017)^28^ | The inclusion criteria for cases and controls were: age 25–45 years, history of infertility for at least 2 years, sperm count <20 × 10˄6 on at least three separate occasions according to World Health Organization guidelines and any kind of infertility cause with exclusion of seminal tract infections and antisperm antibodies, normal plasma concentrations of FSH (1–8 IU/l), LH (2–8 IU/l), testosterone (10–25 nmol/l), partner’s age 25–35 years with no female factors such as ovulatory disorders, tubal factor, endouterine disease, endocrine abnormalities evaluated by hormone assessment, pelvic ultrasound examination, and hysterosalpingography. | Subjects with history of heavy smoking, drug consumption, or alcohol abuse were excluded from the study. |
| Ghazi (2011)^29^ | All patients had a clinically palpable varicocele and subnormal semen parameters, according to WHO criteria, in two or more semen analyses (excluding azoospermia). | Patients with evidence of urogenital infections, leucocytospermia, hypogonadism or a history of smoking, alcohol intake, or recreational drug use were excluded. Patients with serum FSH levels higher than 8 mIU/ml or lower than 1 mIU/ml or with numerical or structural abnormalities in peripheral blood karyotyping were also excluded. Participating patients did not receive any medical treatment for infertility during the 6 months before enrollment in the study. |
| Greco (2005)^30^ | - | Patients with varicocele, genitourinary inflammation, or infection, and smokers were excluded. |
| Gual-Frau (2015)^31^ | None of the selected patients had been pre-treated with antioxidants or other medication before the start of the study. | Patients with genitourinary inflammation, leukocytospermia, history of autoimmune disease, or altered hormonal profiles were excluded from the study. Patients who required treatment during the study for other reasons (mainly influenza) were also excluded. In addition, the following exclusion criteria were applied for women: the absence of hormonal imbalance, chromosomal alterations and tubal obstruction, and the absence of bacterial or viral infection. |
| Habibi (2022)^32^ | Men referred to the Royan Institute clinic- with or without impaired semen analysis with high sperm DNA damage were eligible for our study. For all the couples, the extent of sperm DNA damage was defined with either sperm chromatin structure assay (SCSA) or terminal deoxynucleotidyl transferase dUTP nick end labeling (TUNEL) method, and individuals with values higher than the threshold of 30% and 15% were considered eligible, respectively. | We excluded the subjects with recent/ongoing history of varicocele, leukocytospermia, chemo-radiation, cytotoxic medication, and malignancies. Couples with female-related infertility, including polycystic ovary syndrome, endometriosis, and tubal factor, were excluded from the study. |
| Hajizadeh Maleki (2020)^33^ | Sedentary married men aged between 25 and 40 years old. The patients attending the infertility clinic with a history of infertility over a year, did not demonstrate any hormonal, infective, or physical causes. They neither had any of chronic ailment, serious systemic and urogenital illnesses, or infections nor did they encounter such relevant preceding surgery as either reversal of vasectomy or removal of varicocele. Established via a typical physical examination and routine laboratory tests during the past year, the patients indicating no history of eating disorders as well as depressive illness and having normal physical and sexual development were in good health. Three months prior to the study, no patient was relying upon smoking, alcohol, or antioxidants as supplements (e.g., vitamins and medications such as anabolic steroids) that could possess the potential to modify the hypothalamic-pituitary-gonadal (HPG) axis. In this study, only men with a sedentary lifestyle as well as without any sport or systematic exercise program were included for the experiment. Furthermore, ≥12 weeks prior to the study commencement, all patients were obliged to have ceased all medical therapies. | The patients being exposed to workplaces in which the activity might affect reproductive status were excluded. |
| Huang (2020)^34^ | Infertility for at least 1 year; no medical treatment in the previous 6 months; no presence of varicocele; no smoking; no obesity; no infection of the accessory sex glands; no identifiable cytogenetic abnormalities. | - |
| Humaidan (2021)^35^ | Only patients who had at least two failed IVF/ICSI trials for no apparent reasons, resulting in either embryo developmental arrest, implantation failure, biochemical pregnancy, or miscarriage were invited to participate. Participants were non-smokers, including no intake of cannabis, and were not taking medication with known gonadotoxic effects when entering the IVF program. Moreover, at the first consultation, all patients were advised by the treating physician to reduce exposure to pesticides, irradiation, and excess heat in daily life and work. Controls: healthy male conscript volunteers non-smokers and presumably not suffering from any known disease served as controls. None of the controls had children or fathered previous pregnancies. | Furthermore, patients with sperm concentrations below 5 million/mL in raw semen had a testicular ultrasound to exclude malignancies. Patients with varicocele, malignancies, genetic causes of infertility, and those using any medication were excluded from the study. |
| Jannatifar (2020)^36^ | Inclusion criteria were infertile men with no history of varicocele, obstruction, cancer and chemotherapy as well as abnormal testes, leukospermia, cigarette smoking and alcohol consumption. | - |
| Jannatifar (2021)^37^ | - | Infertile patients with well-known pathologic features such as varicocele, leukospermia, hormonal abnormalities, and/or obstruction, the presence of cryptorchidism, vasectomy, cigarette smoking, alcohol consumption, anatomical disorders, Klinefelter's syndrome, fever in the 90 days prior to sperm analysis, seminal sperm antibodies, were excluded from the study. |
| Kavoussi (2021)^38^ | - | Excluded were men who underwent bilateral varicocele repair, with bilateral testicular atrophy, who had testicular atrophy and a varicocele with a history of cryptorchidism, who had undergone varicocele repair for hypogonadism for orchialgia and not for fertility, and who did not obtain a 3-month postoperative semen analysis because they achieved a pregnancy before then or who did not follow up. |
| Kavoussi (2022)^39^ | The clinical indication for varicocele repair included infertility with baseline abnormal semen parameters, elevated SDF index, or both. Other inclusion criteria included men of 18 years of age and older who had not fathered a child within the previous 12 months, and with sexual abstinence periods of between 2-5 days prior to collection of the semen samples. | Men were excluded from participation if they could not complete all aspects of the study, if they were enrolled in any other clinical studies at the time of screening, if they had a history of prostate cancer, had surgery within 90 days of enrolling in the study, if they took additional daily antioxidant supplements including vitamin c, beta-carnitine, vitamin E, Zinc, N-acetylcysteine, or selenium (daily multivitamin did not require exclusion), or if the man was taking medications for chronic gastrointestinal conditions including irritable bowel syndrome, colitis, or similar metabolic dietary conditions. |
| Kooshesh (2020)^40^ | Patients with iOAT and a serum T:E2 ratio of ≤10, referred to male infertility clinics affiliated with Shiraz University of Medical Sciences, were considered as candidates for inclusion in our study. | Individuals with leukocytospermia (more than 1 million/ml), tobacco or alcohol abuse, ongoing medical treatment (gonadotropins, anabolic steroids, non-steroids anti-inflammatory drugs and cancer chemotherapy), previous cancer radiotherapy or chemotherapy, varicocele and Klinefelter syndrome were excluded in this study. Also, the candidates that their female partners had a history of gynecological problems were excluded from the research. |
| Kumar (2011)^41^ | The inclusion criteria for the study were: 1. Infertility (failure to conceive in 1 year) 2. Semen analysis on two occasions: Normal volume (>2 mL) with sperm counts > 5 mill/mL, and at least one of the following: a. Sperm counts < 20 mill/mL b. Motility < 50% (A+B) or 25% (A) c. Morphology < 50% normal forms. | The following were the exclusion criteria for the study: 1. Identifiable cause for OATs a. Varicocele b. Cryptorchidism (present or treated) c. Orchitis (present or treated) d. Radiation or chemotherapy. Scrotal or pelvic surgery f. Clinical hormonal abnormality 2. Recent febrile illness/medication (within last 3 months) |
| La Vignera (2012)^42^ | Men with grade 3 left varicocele were selected. | To exclude subjects with the concomitant presence of an andrological disease known to alter conventional and nonconventional semen parameters, a complete medical history was collected from each patient. Men (patients and controls) with systemic or endocrine disease; male accessory gland infection; past or present cryptorchidism, microrchidism, cigarette smoking habit, alcohol, or drug abuse; or recent hormonal treatment were excluded. Moreover, patients with less than 50% of viable spermatozoa were excluded from the study. |
| La Vignera (2022)^43^ | - | Exclusion criteria were as follows: the presence of non-HPV-related male accessory gland infection (MAGI), severe oligozoospermia (sperm concentration < 5 mil/mL), any endocrine disease including any type of diabetes mellitus, cigarette smoking, being overweight/obese, mean testicular volume < 12mL (measured using the Prader’s orchidometer), and any drug use. In particular, to exclude the presence of bacterial MAGI, symptomatic patients and those with certain signs at the physical examination (tenderness of the epididymis or vas deferens, or abnormal rectal exploration) underwent sperm and urethral swab cultures. Patients with positive cultures or with signs/symptoms strongly suggestive of bacterial infection were excluded. |
| Lara-Cerrillo (2020)^44^ | Fertile donors from a semen bank as a control group. These men did not present varicocele or other fertility impairments and had normal seminal parameters. 20 patients (aged 17–44) were recruited at Parc Tauli Hospital (Sabadell, Spain) when they presented with clinical unilateral or bilateral grade II or higher varicocele detected by ultrasonography with a venous dilatation greater than 3mm and presence of blood flow reflux, had a diagnosis of infertility and were candidates for MV. | Exclusion criteria included: (i) evidence of urogenital infections; (ii) absence of varicocele after physical examination and ultrasound; (iii) regular consumers of alcohol, drugs and tobacco; (iv) recurrent varicocele after surgery; or (v) presenting with late complications after surgery. |
| Li (2012)^45^ | Those selected as controls had normal seminal parameters, no evidence of endocrine or systemic disease, and no past history of cryptorchidism, varicocele, or orchitis. | Men with azoospermia and evidence of genital tract infection were excluded. |
| Lipovac (2021)^46^ | All subjects with abnormal testing’s were offered the active treatment, in addition to lifestyle changes (i.e. healthy diet, exercise, and quit toxic habits such as alcohol, cigarette, illicit drugs, etc.). Those who decided for the active therapy must also have proven compliance for the intake of the standardized micronutrient compound. | Exclusion criteria was azoospermia, aspermia, varicocele, and recent urogenital infections. |
| Maghsoumi-Norouzabad (2021)^47^ | Inability to have a child after at least 1 year of marriage without the use of preventive measures, normal fertile woman partner, no medical history that could cause infertility, no drug or alcohol abuse, no job and environmental exposure to toxins that can cause infertility, a body mass index (BMI) less than 30 kg/m2, and no medical therapy≤3 months prior to research initiation. | Exclusion criteria included any acute illness, admission of less than 90% of the supplement, leaving the study based on the personal desire of the participants, attendance at another research study, immigration, and unavailability for follow-up. |
| Martínez-Soto (2016)^48^ | In the study, men over 18 years old, who were undergoing evaluation for infertility during the period 2009–2010 were included. | Oncological patients, those suffering from metabolic disease, chromosomal, or genetic alterations, and patients with anticoagulant treatment were excluded. |
| Ménézo (2007)^49^ | - | - |
| Micic (2019)^50^ | All participants met the inclusion criteria of one semen analysis that demonstrated either total sperm number ≤15 million per ml; progressive motility <32%; normal viscosity and normal leucocytes number (<1 × 10^6^/ml); total ejaculate volume 1.0 ml; sperm vitality ≤58% live; normal sperm morphology <4% (according to WHO, 2010). | Exclusion criteria for the male participants were as follows: motility <5%; sperm concentration <1 × 106/ml; history of undescended testes; subjects with known hypersensitivity to ingredients in Proxeed Plus; endocrine disorders affecting the hypothalamic–pituitary axis; history of post‐pubertal mumps; presence of anti‐sperm antibodies; history of endocrine disease; autoimmune disease, cystic fibrosis, or testicular cancer; leucocytospermia, leucocyte count >1 × 106/ml; use of antioxidant agents or vitamins within the 8 weeks prior to inclusion in the study (for subjects using vitamin supplementation, an 8‐week wash‐out period was required prior to inclusion in the study); use of vitamin or natural treatment for infertility at any time; history of taking any therapy for infertility within the last 2 months including over‐the‐counter treatment and vitamin supplementation; history of excessive consumption of alcohol 90 days prior to the start of the trial; subjects involved in other clinical trials. |
| Miñambres (2022)^51^ | All patients included were studied before (baseline) and at 6, 12, and 18 months after bariatric surgery. | We excluded patients above 55 years old and patients with kidney, liver, gonadal, or pituitary disease or concomitant treatments that could disturb/interfere with sexual hormone concentrations or semen parameters. |
| Mir (2018)^52^ | Men with BMI over 25 kg/m² | We excluded two men diagnosed with Klinefelter’s syndrome, three with Y-chromosome microdeletion, two with retractile testis, seven with varicocele and seven men with azoospermia were excluded because of inadequate sperm count to perform the test. |
| Mohammed (2015)^53^ | Men with at least 1-year history of infertility, a palpable varicocele, oligo, atheno, or teratozoospermia were selected from our andrology clinic. | - |
| Moskovtsev (2010)^54^ | Men who had persistently high DNA damage (>30%) | Patients who were receiving concurrent antibiotic therapy or varicocelectomy were excluded from the study. |
| Moskovtsev (2009)^55^ | Eighty-four infertile patients with initial sperm DNA damage greater than 30% as measured by DFI, who completed a course of treatment followed by successive DNA assessments, were included in this retrospective study. | - |
| Nazari (2020)^56^ | Males and females were analyzed and were normal chromosomal karyotypes. Couples suffering recurrent pregnancy loss. | Male partners having normal semen parameters. |
| Negri (2017)^57^ | Inclusion criteria were oligo-normozoospermia, according to the 2010 World Health Organization criteria for the Evaluation of Human Semen (5th Edition); Sperm DNA fragmentation > 15%; no current seminal infections according to sperm culture and/or seminal leucocyte (< 106 x ml); no sperm antibodies (Sperm Mar IgG; Ferti Pro, N.V., Origio, Florence, Italy); no varicocele. These patients had i) mildly or severely high levels of DNA fragmentation at baseline examination and ii) SCD control after 2 months. | Indeed, we only excluded patients with varicocele and seminal infections, as diseases associated with SDF, but susceptible to effective specific treatment (antibiotics and surgery). Patients with antisperm antibodies were excluded. |
| Ni (2014)^58^ | Males with oligozoospermia (less than 15x10^6⁄ml) and/or asthenozoospermia (less than 32% progressive motility) and left clinical varicocele were included in this study. | Exclude aspects that might be related to male primary infertility, such as genital infection, hypogonadism, ductal obstruction, etc. |
| Ni (2016)^59^ | An infertility history of longer than 12 months despite regular unprotected intercourse | No other infertility related diseases, such as genital infection, hypogonadism, etc, no occupational agents and factors deteriorating male fertility, such as pesticides, solvents, heat, radiation, etc , no other factors affecting seminal ROS level such as leukocytospermia, age>40 years, antioxidant therapy (Vitamin C, Vitamin E, Coenzyme Q, etc), and no obvious causes of infertility in the female partner. |
| Ozer (2020)^60^ | Patients with DNA damage of 30% or more were included study. | Presence of varicocele, leukocytospermia, known genetic abnormality, history of chemotherapy and/or radiotherapy, history of malignancy, history of orchiectomy and/or orchiopexy and patients receiving hormonal therapy were accepted as exclusion criteria. |
| Özgök Kangal (2021)^61^ | The inclusion criteria were (i) patients who had a history of infertility for at least one year, (ii) patients who did not have any urogenital tract abnormalities, (iii) patients whose spouses did not have any problems in terms of infertility evaluation, (iv) patients for whom live birth has never been achieved spontaneously or with ARTs at the first admission, (v) patients older than 18 years, and (vi) patients who had attended to HBOT 3 months before the ART processes’ beginning. All patients with varicocele (100%) had varicocelectomy before HBOT. | - |
| Palomba (2011)^62^ | At least one of the following abnormalities in sperm concentration (<20 million sperm/ml), motility (>30%/<50% motile sperm) and morphology (>30%/<50% normal morphology) was needed to meet the inclusion criteria. All participants had a normal level of physical activity and were non-smokers; none was known to abuse alcohol. | As specific exclusion criteria were considered the presence of abnormal plasma FSH (reference value: 0.70-11.0 U/l), LH (reference value: 0.8-8.1 U/l), PRL (reference value: 1.7-18 μg/l), TSH (reference value: 0.3-5.0 U/l), and testosterone (T) (reference value: 3.0-10.0 ng/ml) levels, gonad abnormalities or varicocele, history of cryptorchidism, orchitis, testicular torsion or trauma, infections as evaluated by sperm culture after sperm collection and/or urethral sampling as required, seminal antisperm antibodies, Y chromosome microdeletions, and cytological testicular pattern of hypospermatogenesis with maturation disturbances. Also excluded were current or previous (within the last six months) use of hormonal, anti-diabetic, anti-obesity or antioxidant drugs; intention to adopt a diet and/or a specific physical activity program. |
| Pelliccione (2011)^63^ | - | - |
| Pini (2020)^64^ | - | Patients were excluded from the study if surgical sperm retrieval was required, the ejaculate did not contain enough spermatozoa to perform ICSI (<5 × 106 spermatozoa/mL), or there was a history of vasectomy reversal. |
| Sakamoto (2008)^65^ | All those who had grade II or grade III clinical varicocele had a microsurgical subinguinal varicocelectomy. | Patients with positive leukocytospermia or with subclinical varicocele (grade I) were excluded from the study. |
| Salas-Huetos (2018)^66^ | The study participants were healthy men (18–35 y old) who reported routinely eating a Western-style diet [according to the 15-item dietary screener modified from Martínez-González et al. | Participants were excluded if they had one of the following exclusion criteria: frequent consumption of nuts or a known history of allergy; use of plant sterol or fish oil supplements and multivitamins, vitamin E, or other antioxidant supplements; history of reproductive disorders or vasectomy; current smokers; use of medications for chronic illness; or use of illegal drugs. |
| Salehi (2019)^67^ | - | Those that had a history of varicocele, surgery, and inflammation were excluded from our study. |
| Samavat (2018)^68^ | - | - |
| Scaruffi (2021)^69^ | We recruited 86 couples who had a history of a homolog ICSI attempt using fresh oocytes and ejaculated sperm with unexplained <60% fertilization rate, inseminated oocytes >6, and female partner <38 years old. Male inclusion criteria: sperm count > 1 × 106/mL and no concomitant consumption of drugs. | Patients with azoospermia or severe oligozoospermia (sperm count less than 1 × 10^6^/mL) or with an identifiable cause of infertility (leukocytospermia and/or positive sperm culture, epididymoorchitis, prostatitis, inguinoscrotal surgery, cryptorchidism, varicocele, etc.) were excluded. Female partners with diminished ovarian reserve (less than six retrieved oocytes in a prior IVF cycle or elevated early follicular phase FSH (follicle-stimulating hormone) or less than 1 ng/mL Anti-Müllerian Hormone concentration in serum) were excluded from the study. We excluded any type of endocrine, metabolic, autoimmune, and neoplastic diseases in both female and male partners. |
| Simoni (2016)^70^ | Inclusion criteria were: age 20–50 years, idiopathic male factor infertility for at least 1 year, homozygous FSHR p.N680S N or S genotype, sperm DFI. 15%, normal serum FSH levels (≤8 IU/l), normal serum LH, testosterone, prolactin and estradiol levels, as measured at the recruiting centers and considering the reference range of the method used in each center. A normal ovulatory female partner was required. | Exclusion criteria were azoospermia, all known etiologies of male infertility, such as endocrine disorders, varicocele, cryptorchidism, infections, immunological infertility and obstructive infertility, and genetic disorders causing infertility, such as chromosome abnormalities, congenital bilateral absence of the vas deferens and microdeletions within the AZF regions of the Y chromosome. Moreover, all known etiologies of female infertility in the partner (such as tubal occlusion, endometriosis, endocrine abnormalities including anovulation and polycystic ovaries, anatomical abnormalities, infections), heterozygous FSHRp.N680S genotype, drug abuse, major systemic diseases and primary testicular failure were reasons for exclusion. |
| Smit (2013)^71^ | Men with at least a 1-year history of infertility, a palpable varicocele, oligospermia and normal or correctable female fertility were eligible. | - |
| Steiner (2020)^72^ | Couples:  12 or more months of infertility (primary or secondary)  Heterosexual  Cohabitating and able to have regular intercourse  Males:  ≥ 18 years of age  At least one abnormal semen parameter on a semen analysis within the past 6 months:  Sperm concentration ≤15 Million/ml  Total motility ≤40%  Normal morphology (Kruger) ≤4%  DNA fragmentation (SCSA, DNA fragmentation index) >25%  Females:  ≥18 years of age and ≤40 years of age  For women ≥ 35 years of age, evidence of normal ovarian reserve as assessed by menstrual cycle day 3 (+/-2 days) FSH ≤10 IU/L with estradiol ≤ 70 pg/mL, AMH ≥ 1.0 ng/mL, OR antral follicle count >10 within one year prior to study initiation.  Evidence of at least one patent fallopian tube as determined by an hysterosalpingogram or laparoscopy showing at least one patent fallopian tube or a saline infusion sonogram showing spillage of contrast material  Regular cycles defined as ≥25 days and ≤35 days in duration  Evidence of ovulation including biphasic basal body temperatures, positive ovulation predictor kits, or progesterone level ≥3 ng/ml. | Couples:  Previous sterilization procedures (vasectomy, tubal ligation)  Planning in vitro fertilization in the next 6 months  Males:  Sperm concentration < 5 million/mL on screening semen analysis  Current use of a medication or drug that would affect reproductive function or metabolism  Current multivitamin or herb use (requires 1 month wash-out)  Current serious medical illnesses, such as cancer, heart disease, or cirrhosis  Current use of anticoagulants  Untreated hypothyroidism  Uncontrolled diabetes mellitus  Females:  History of surgically or medically confirmed moderate or severe endometriosis  Body mass index >35 kg/m2  Currently pregnant  History of polycystic ovarian syndrome  Current serious medical illnesses, such as cancer, heart disease, or cirrhosis  History of systemic chemotherapy or pelvic radiation  Current use of a medication or drug that would affect reproductive function or metabolism |
| Stenqvist (2018)^73^ | Previously performed semen analysis showed DFI ≥25%. The inclusion criteria were as follows:  1. Age: 18–50 years,  2. Non-smoking,  3. Not being treated with antihypertensive drugs, hormones, statins,  psychotropic drugs or oral cortisone for the last six  months,  4. No history of anabolic steroids use,  5. Not taking antioxidant supplementation for the last six  months. | Excluded if:  1. Body mass index (BMI) ≥30,  2. FSH outside the normal range of 2–8 IU/L, (g)  3. LH outside the normal range of 2–10 IU/L,  4. T < 10 nmol/L,  5. DFI <25% in a repeated semen sample. |
| Sun (2018)^74^ | - | The exclusion criteria were: normal semen parameters, azoospermia, other causes of male infertility that has been medical proven, advanced female partner age ≥ 38 years, detected female causes infertility with medical proven evidence. |
| Telli (2015)^75^ | Men with at least 1-year history of infertility, a palpable varicocele and oligospermia. | All patients with urinary tract infections, fever occurring ~90 days before semen analysis, leukocytospermia, systemic diseases such as cancer and endrocrinopathies that would lead to testicular alterations and a history of smoking and excessive alcohol or drug use were excluded from study. |
| Vahidi (2021)^76^ | The primary inclusion criteria were (i) couples with recurrent early abortion (more than two abortions in the first trimester) and (ii) recurrent failed ART (more than three intrauterine inseminations (IUI) or more than two in vitro fertilisation (IVF) sessions and more than two intracytoplasmic sperm injection (ICSI) sessions). Men with disturbed DNA integrity, as assessed using terminal deoxynucleotidyl transferase-mediated dUTP nick end labelling (TUNEL) test, and/or increased level of chromomycin A3 (CMA3) staining (TUNEL score > 20% and/or CMA3 score > 30) were selected and treated with antioxidant medical therapy. | Exclusion criteria were (i) a history of smoking, (ii) opium addiction, (iii) multiple sexual partners, (iv) known hormonal abnormality, (v) clinically detected varicoceles, or (vi) cryptorchidism. The same andrologist performed a complete physical examination, evaluated the past medical history and recorded the results. |
| Verdi (2020)^77^ | The inclusion criteria were the low level of FSH (<1.7 mIU/mL), aged 25–45 years, history of infertility for at least 2 years, sperm concentration <15×10^6 (oligozoospermia) according to 2010 World Health Organization criteria. | - |
| Wald (2021)^78^ | All patients with clinical diagnosis of varicoceles who subsequently underwent varicocelectomy for any indication were included. | Men were excluded if data were missing regarding surgical technique. Patients with exogenous testosterone supplementation pre-operatively or post-operatively were excluded from analysis. |
| Wang (2012)^79^ | Varicocele patients, aged 20-50 years, asthenozoospermic, negative antisperm antibodies. | Retrograde ejaculation or anejaculation. Those taking anti-epileptic, anti-tumor and other drugs that hinder spermatogenesis and sperm motility. Congenital malformations, insemination pathway obstruction, testicular atrophy (including epididymis), chromosome abnormality. Abnormal sex hormone secretion. Complicated with serious primary diseases such as cardiovascular, liver, kidney and hematopoietic system, mentally ill. Those who do not meet the inclusion criteria and the data are incomplete. The patient does not agree to participate. |
| Werthman (2008)^80^ | All patients had a DNA fragmentation index over 27% to 30% (fair to poor sperm DNA integrity) and had no other potential obvious reasons for high levels of sperm DNA fragmentation and infertility except for the presence of a varicocele(s) | - |
| Yakovlev (2022)^81^ | Included were men, who gave informed consent, were aged between 25 to 45 years, with sperm DNA fragmentation over 15%, but no more than 50%, with confirmed infertility: no pregnancy achieved within at least a year with regular unprotected sexual intercourse. | - |
| Yuan (2021)^82^ | Infertile men aged 28–35 years old who had normal standard parameters of semen but a higher sperm DFI (above 30%) were enrolled in the present study. Their partners had apparently normal parameters such as patent Fallopian tubes, regular menstrual periods, and normal anti-Müllerian hormone (AMH) and ovarian follicle counts. | - |
| Zaazaa (2018)^83^ | Inclusion criteria for the patients were stable relationship, Vx grades II & III, and normal female factor. | Exclusion criteria were leucocytospermia, azoospermia, smoking, diabetics, hypertension, and patients with sperm DNA fragmentation index (DFI) <30%. |
| Zhang (2022)^84^ | The inclusion criteria are two or more times semen analysis according to the 2010 World Health Organization (WHO) criteria for classifying sperm. The antisperm antibody test was negative. The inclusion criteria for healthy men were as follows: reproductive history in the past year and no abnormality in the current sex hormone levels, semen analysis, or other indices. | Diagnostic exclusion criteria for patients with idiopathic male infertility included abnormal sexual life and ejaculatory disorders such as nonejaculation or retrograde ejaculation, chromosomal abnormalities, congenital malformations, cryptorchidism, testicular dysplasia, testicular atrophy, injury, varicocele, hypogonadism, leukospermia, vas deferens obstruction, sex chromosomal aneuploidy/mosaicism, Yq microdeletion, and reproductive system infection (including male accessory gland infection). Those taking anti-epilepsy, antitumor, antirheumatic, and anti-rheumatoid drugs that hinder spermatogenesis and sperm motility, as well as those who had used antioxidants in the past, were excluded. Patients with serious primary diseases, metabolic diseases, and mental disorders (such as cardiovascular, liver, kidney, and hematopoietic system diseases), smoking (more than 10 cigarettes a day for more than half a year), and alcoholism (50 ml pure alcohol daily for more than half a year) were excluded, as well as those who did not agree to participate in the clinical study and did not sign a clinical research agreement. The exclusion criteria for healthy men were the same as those for patients with idiopathic male infertility, those with abnormal semen analysis and physical examination, and subjects or family members who refused to provide consent to participate in the study. |
| Zhao (2019)^85^ | Oligozoospermia was demonstrated in at least three semen analyses performed within a period of 6 months. The men met the following inclusion criteria: infertility for at least 1 year; no medical treatment in the previous 6 months; no presence of varicocele; no smoking; no infection of the accessory sex glands; and no identifiable cytogenetic abnormalities. All of the wives (mean age 29.6 ± 3.1 years, range 23–35 years) received a complete infertility workup to rule out female factors. | - |
| Zini (2011)^86^ | Men presenting to our clinic with one year or more of infertility, a clinically palpable varicocele and abnormal semen parameters (reduced sperm concentration, motility, or morphology on two or more semen samples) were deemed to be candidates for varicocele repair. | Men with azoospermia, severe oligozoospermia (<5 million sperm ⁄ mL), complete asthenozoospermia or evidence of genital tract infection were excluded. |

**Supplementary Table 4.** Intervention and population definitions in each included article

| **Author (year)** | **Intervention** | **Intervention definition** | **Population or fertility status definition** |
| --- | --- | --- | --- |
| Abad (2013) ^1^ | Antioxidant | oral antioxidant treatment (1500 mg of L-Carnitine; 60 mg of vitamin C; 20 mg of coenzyme Q10; 10 mg of vitamin E; 10 mg of zinc; 200 lg of vitamin B9; 50 lg of selenium; 1 lg of vitamin B12) during a time period of 3 months. 3 months of antioxidant treatment with commercial multi-vitamins (Androferti, Q Pharma Laboratories; Alicante, Spain) containing L-Carnitine (1500 mg), vitamin C (60 mg), coenzyme Q10 (20 mg), vitamin E (10 mg), vitamin B9 (200 lg), vitamin B12 (1 lg), Zinc (10 mg) and selenium (50 lg). | Infertile: at least 2 previous semen analyses showing asthenoteratozoospermia |
| Abbasi (2021)^2^ | Antioxidant | While the subjects in the treatment group were designated to receive a single capsule (500 mg) of FamiLact on a daily basis for 80 consecutive days, the controls took the identical placebo (Figure 1) (11). FamiLact is formulated by Zist Takhmir Pharmaceutical Company, under the permission of the Food and Drug Department of Iran’s Ministry of Health and Medical Education (reference no.: 0347756442342525). Each capsule of FamiLact contains bacterial strains of Lactobacillus rhamnosus, Lactobacillus casei, Lactobacillus bulgaricus, Lactobacillus acidophilus, Bifidobacterium breve, Bifidobacterium longum, Streptococcus thermophilus (10˄9 CFU), and  fructooligosaccharides as prebiotic. | Infertility: failure to establish a clinical pregnancy following 12 months of regular, unprotected sexual intercourse |
| Abdelaziz (2015) ^3^ | Varicocelectomy | Extensive laparoscopic varicocelectomy: Both external and ISVs were ligated and divided, the testicular artery and lymphatic vessels were spared. | Infertility: at least 1‑year |
| Abdelbaki (2017) ^4^ | Varicocelectomy | Testicular artery- and lymphatic sparing inguinal varicocelectomy using 3 loupe magnification | Infertile: more than 12 months. Control: WHO - normozoospermic healthy men |
| Afsin (2018)^5^ | Varicocelectomy | - | - |
| Alahmar (2020)^6^ | Antioxidant | Each patient received CoQ10 200 mg/d orally (one a day) for three months. The seminal plasma concentrations of CoQ10 were measured by high-performance liquid chromatography (HPLC) method using a UV detector at 275 nm | Infertile (more than 12 months) patients with idiopathic OA, fertile: healthy men |
| Alahmar (2021)^7^ | Antioxidant | 200 mg/day CoQ10 for 3 months | Infertile: for minimun 1 year, 50 fertile (fathered a child in last 24 months+normal semen analysis) |
| Alahmar (2022)^8^ | Antioxidant | The first group received a daily dose of 200 mg of CoQ10 (in the form of ubiquinol) (AMS, Woodinville, WA, USA) as a single oral dose for 3 months. The second group received Centrum multivitamins (Pfizer, New York, NY, USA) as 1 tablet per day orally containing 26 vitamins and minerals for 3 months. | Fertile controls had a history of fathering a child in the last 2 years, with normal seminal fluid analysis findings and normal female fertility assessment. The patients included in the study had a history of infertility of 1 year or more without the use of contraception. OA was defined according to the World Health Organization (WHO) 2010 criteria |
| Alhathal (2016)^9^ | Varicocelectomy | Microsurgical sub-inguinal varicocelectomy | Clinically palpable varicocele and abnormal semen parameters, healthy controls with normal semen parameters |
| Arafa (2020)^10^ | Antioxidant | Antioxidant supplement ‘FH PRO for Men’ for a period of three months: provides the following amounts of these nutrients per day: vitamin A (as beta-carotene): 5000 IU, vitamin C: 120 mg, vitamin D3: 1200 IU, vitamin E (as mixed tocopherols): 200 IU, vitamin K: 80 µg, thiamin: 3 mg, riboflavin: 3.4 mg, niacin: 20 mg, vitamin B6: 25 mg, folate: 800 µg, vitamin B12: 1000 µg, biotin: 600 µg, pantothenic acid: 20 mg, iodine: 150 µg, zinc: 30 mg, selenium: 140 µg, copper: 1 mg, manganese: 2 mg, chromium: 120 µg, molybdenum: 75 µg, l-carnitine tartrate: 2000 mg, l-arginine: 350 mg, CoQ10: 200 mg, N-acetyl l-cysteine: 200 mg, grapeseed extract: 20 mg, lycopene: 10 mg, and benfotiamine: 1 mg. | A total of 148 infertile men of unknown etiology were divided into idiopathic (n = 119) and unexplained male infertility (UMI; n = 29). On the basis of semen analysis, subjects enrolled were categorized into idiopathic and UMI. The idiopathic infertility group included men with abnormal semen analysis defined as having at least one sperm parameter (sperm concentration >1 and ≤15 million per mL, total sperm motility ≤40%, or sperm morphology as evaluated by strict criteria with normal forms ≤4.0%). In the UMI group, infertile men with normal semen parameters (sperm concentration >15 million per mL, total sperm motility >40%, or sperm morphology as evaluated by strict criteria with normal forms >4.0%) were included. |
| Baker (2013)^11^ | Varicocelectomy | Subinguinal microsurgical varicocelectomy | Patients with varicocele |
| Boeri (2022)^12^ | Antioxidant | SOD (superoxide dismutase) + (SOD-based antioxidant plus hydroxytyrosol and carnosol; FertiPlus® SOD) or SOD− (any other antioxidants without SOD, hydroxytyrosol and carnosol) for no less than 3 months. Of note, SOD− compounds included a combination of vitamin C, vitamins B, Coenzyme Q10, myo-inositol, selenium and zinc. The choice of the treatment was decided based on patient and physician preference. | Infertile white European men. According to the WHO criteria, infertility is defined as not conceiving a pregnancy after at least 12 months of unprotected intercourse regardless of whether or not a pregnancy ultimately occurs. |
| Cannarella (2021)^13^ | Antioxidant | Oral selenium supplementation at the dose of 83 µg once daily (Syrel®, IBSA) for six months | Patients were those of reproductive age, with normal thyroid function, and with at least one abnormal conventional sperm parameter (sperm concentration, total sperm count, motility, or morphology) |
| Capece (2017)^14^ | Antioxidant | Group A received myo-inositol 1000mg, Tribulus Terrestris 300 mg, Alga Ecklonia Bicyclis 200 mg and Biovis one tablet a day for 90 days, and group B (placebo group) received one placebo tablet a day | OATs patients |
| Colacurci (2018)^15^ | FSH | All enrolled patients received the recombinant FSH (Gonal-f RPen) treatment, subcutaneously administered, at a 150 IU dosage every other day for a total period of 3 months. | Infertile couple: no pregnancies after at least 12 months of unprotected intercourse |
| Colacurci (2012)^16^ | FSH | The patients were randomized into 2 groups: 65 men were treated on alternate days for 90 days with injections of 150 IU rFSH (Gonal-F, Serono, Rome; study group A), and 64 subjects received non-antioxidant vitamin supplements (control group B). | Idiopathic OATs patients |
| Condorelli (2021)^17^ | FSH | The first group (n = 24) was prescribed highly purified FSH (hpFSH) 75 IU/daily (Group A), and the second group (n = 24) was prescribed hpFSH 150 IU three times a week (Group B) for three months. All patients were treated for 3 months. | Infertile: referred for male infertility |
| D’argent (2021)^18^ | Antioxidant | The men were then randomized in a 1:1 ratio to receive daily supplements either containing 15 mg (3 tablets of 5 mg each) of folic acid or a placebo (3 tablets a day) for 3 months as from Day 0. The study tablets were manufactured to match in appearance, size, taste and weight (Bailly-Creat Laboratory). | Male partners of infertile couples wishing to conceive, aged 18 to 60 years old, and requiring IVF-ICSI treatment for a male factor. |
| Dadfar (2021)^19^ | Varicocelectomy | Men were selected and studied with primary infertility and varicocele that were candidates for varicocelectomy surgery and were selected from hospitals in Ahvaz to compare the changes in DNA fracture index. Patients in terms of body mass index were divided into three groups with BMI less than 25 as normal weight, 25 to 30 as overweight, and 30 above as obese. | Patients underwent microscopic varicocelectomy subinguinal surgery by the single surgical team, their postoperative status was determined and after ensuring the absence of active varicocele (recurrence). |
| Dadgar (2022)^20^ | Antioxidant | Pentoxifylline group received 400 mg pentoxifylline twice daily, zinc group, 15 mg zinc once daily, pentoxifylline + zinc group, 400 mg pentoxifylline (twice daily), and 15 mg zinc (once daily), and placebo group, with placebo tablets twice daily for 3 months. The prescribed doses of pentoxifylline and zinc were selected based on pilot studies and urologist diagnosis. | Idiopathic infertile men (including oligo-asthenozoospermia, asthenozoospermia, astheno-teratozoospermia, oligoastheno-teratozoospermia) |
| Dhawan (2018)^21^ | Lifestyle | The sessions were conducted for an average of two hours per day under the direct supervision of registered, specialized yoga instructor. This integrative health strategy program included a series of physical postures (asanas), breathing exercises, meditation and each session ended with relaxation through Shavasana. The typical session of YBLI involved an interactive session with the patients | Male partners of couples experiencing idiopathic RPL. |
| Elsedfy (2018)^22^ | Antioxidant | Participants received L-carnitine: 2 g/ day and N-acetyl cysteine: 600 mg/day for 6 months with a monthly review to check drug compliance and to provide treatment. | Beta thalassaemia major patients - 10 azoospermic, 10 zoospermic. All participants had a DNA FI of less than 30% before treatment. |
| Esfahani (2010)^23^ | Varicocelectomy | - | Infertile with varicocele: grade 2 or 3, fertile control: no varicocele. Fertile controls' wives were pregnant at the time of study. |
| Fathi (2021)^24^ | Varicocelectomy | Microsurgical subinguinal varicocelectomy using a Karl Zeiss operating microscope with a magnification of ×10–20, using the standard surgical technique. | Subfertile: at least a 1-year history of male factor subfertility |
| Fornaro (2009)^25^ | FSH | 150 IU recombinant FSH every other day for at least 3 months. | Infertile: idiopathic oligoasthenoteratozoospermic men attending infertility center. |
| Gabriel (2012)^26^ | Varicocelectomy | Microsurgical sub-inguinal varicocelectomy. | Infertile: one year or more history of infertility. Fertile: sperm donors with normozoospermia. |
| Gallegos (2008)^27^ | Antibiotics+anti-inflammatory | Then antibiotic therapy was chosen considering the antibiogram of Mycoplasma culture and the tolerance of the patient, including a macrolide, a tetracycline, or a quinolone, during a period depending on the evolution of symptoms and laboratory follow-up. | Semen samples were collected from 143 male members of couples attending the andrology infertility clinic with diagnosed genitourinary infection from Chlamydia trachomatis and Mycoplasma. All patients (n ¼ 143) infected by Chlamydia trachomatis were also positive for different concentrations of Mycoplasma. Fertile: not defined. |
| Garolla (2017)^28^ | FSH | Each treated patient received 150 IU highly purified urofollitropin (uFSH, Fostimon, IBSA, Lugano, Switzerland) three times a week for 3 months. | Infertile: oligozoospermic infertile patients from couples scheduled for ARTs. |
| Ghazi (2011)^29^ | Varicocelectomy | Microsurgical varicocelectomy | Infertile: infertility for at least 1 year |
| Greco (2005)^30^ | Antioxidant | Members of one group were given a 2-month oral treatment with 2 antioxidants, vitamin C and vitamin E, both at a daily dose of 1 g (500 mg twice a day). Members of the other group received a placebo during the same period. | Infertile: consulting for infertility |
| Gual-Frau (2015)^31^ | Antioxidant | Three months of antioxidant treatment with commercial multivitamins (Androferti, Q Pharma Laboratories; Alicante, Spain) containing L-Carnitine (1500 mg), vitamin C (60 mg), coenzyme Q10 (20 mg), vitamin E (10 mg), vitamin B9 (200 μ g), vitamin B12 (1 μ g), zinc (10 mg), and selenium (50 μ g) per day. | Infertile men with asthenoteratozoospermia and clinical grade I varicocele. |
| Habibi (2022)^32^ | Antioxidant | Patients in the case group received a cumulative daily dose of 600 mg of alpha-lipoic acid (ALA) (Raha company, Isfahan, Iran), while controls were given the placebo (600 mg, made of starch) with identical appearance and taste, both daily for 80 consecutive days (18). There are no reports of adverse drug reactions for the oral intake of ALA in the literature and it is considered safe to consume | Infertile men with sperm DNA damage over the threshold (30 or 15% threshold for impaired and non-impaired semen analysis) |
| Hajizadeh Maleki (2020)^33^ | Lifestyle | All the patients in the EX group underwent a supervised treadmill HIIT, three times a week during 24 weeks, between 5 and 7 pm. During the initial 12 weeks of the study, 10 sets of higher workloads (75–85% of VO2max) were interspersed every minute with lower workloads (50–55% of VO2max). During the final 12 weeks of training protocol, 15 sets of higher workloads (85–95% of VO2max) were interspersed every minute with lower workloads (50–55% of VO2max). In each training session, the patients performed a 10–15 min warm-up (60–65% VO2max) as well as a five-minute cool-down (50% VO2max) | Infertile: infertility for over a year |
| Huang (2020)^34^ | Antioxidant | Patients in folic acid treatment group received folic acid at the dose of 0.8 mg/day for 3 months, and the patients serving as the placebo group received starchfilled capsules for 3 months. | Infertile men: suffering from oligozoospermia with sperm counts consistently below 15 million per mL. Different groups based on MTHFR genotypes. |
| Humaidan (2021)^35^ | Lifestyle | All males with a DFI >15% were asked to embark on a three-month lifestyle intervention program based on a questionnaire consisting of lifestyle parameters. The patients received individualized recommendations as to lifestyle intervention according to their answers to a structured questionnaire including a total 68 of questions, covering among others smoking, alcohol consumption, caffein intake, diet, exercise, body weight, stress, and work-related exposure. The proposed lifestyle interventions included primarily general health recommendations for adults from the Danish Health Authority. In brief, at least 30 minutes of exercise daily was recommended. Regarding nutrition and food consumption, a reduction in meat intake (maximum of 500g/week) and, in particular, caution about red meat, more intake of fruit and vegetables, and reducing high sugar-containing drinks, including soda and energy drinks were recommended. Moderate alcohol intake <6 units per week was also recommended. All patients embarking on our IVF program were already non-smokers by recommendation. We considered any potential work-related exposures and recommended, for example, facemasks to reduce airborne exposure when relevant. In case of a subjective feeling of stress, we recommended mindfulness. Finally, we advised against having the cellphone in the trouser pocket and the laptop on the lap. Three-month lifestyle intervention period. | Infertile: men with idiopathic or unexplained infertility undergoing MAR treatment |
| Jannatifar (2020)^36^ | Antioxidant | NAC (600 mg daily, for three months) | Infertile: idiopathic asthenoteratozoospermia |
| Jannatifar (2021)^37^ | Antioxidant | NAC (600 mg/day) orally for three months | Infertile: astheno-terato-azoospermic (ATZ) individuals |
| Kavoussi (2021)^38^ | Varicocelectomy | All varicoceles were repaired by a subinguinal microsurgical technique. | Infertile post varicocele repair. Testicular atrophy (TA) and no testicular atrophy groups (NTA). |
| Kavoussi (2022)^39^ | Varicocelectomy | Microsurgical subinguinal varicocele repair | Infertile: male patients evaluated for infertility by a reproductive urologist and diagnosed with a clinically palpable varicocele |
| Kooshesh (2020)^40^ | Letrozole | All the participants were orally treated with 2.5 mg letrozole (Soha Pharma Company, Iran) per day for 3 months. | Idiopathic OATs: men with low serum testosterone (T) to estradiol (E2) ratio (T:E2 ratio ≤ 10). |
| Kumar (2011)^41^ | Antioxidant | The drug (herbomineral supplement, Addyzoa®) and placebo were prescribed in a dose of two capsules twice a day for 3 months. Drug contains: Powders (Purnachandrodaya rasa, Suvarnavang, Muktashukti bhasma, Suvarnamakshik bhasma, Shilajit shuddha, Abhrak bhasma, Makardhwaj rasa, Rasa sindur), Extracts (Gokshur (Tribulus terrestris), Ashtavarga, Shwet musli (Chlorophytum arundinaceum), Kapikachchhu shuddha (Purifi ed Mucuna pruriens), Guduchi (Tinospora cordifolia), Ashwagandha (Withania somnifera), Amalaki (Emblica offi cinalis), Balamool (Sida cordifolia), Vridhadharuk (Argyreia speciosa), Shatavari (Asparagus racemosus), Varahikand (Tacca aspera), Chopchini (Smilax china), Vidarikand (Ipomoea digitata), Munjatak (Eulophia campestris)) | Infertility was defined as the inability to conceive after at least 1 year of regular, unprotected intercourse with the partner. (idiopathic OATs) |
| La Vignera (2012)^42^ | Varicocelectomy | Subinguinal microsurgical varicocelectomy (SMV) | Men with grade 3 left varicocele were selected. The control group comprised healthy, normozoospermic (same exclusion criteria) and presumptively fertile men (fatherhood in the 12 months previous to the study) without varicocele and with similar age and body mass index (25.2 6 4.4 years old, range 20–30 years; body mass index range: 19.0–24.0 kg/m2). |
| La Vignera (2022)^43^ | Varicocelectomy | Two groups according to the clinician’s decision at the time of their visits on whether to administer ellagic acid (100 mg) and annona muricata (100 mg) or not. The two molecules were given in a single tablet formulation for three months (Group A; 25 patients), or the patient was re-evaluated for HPV DNA presence after three months of active surveillance only (protected sexual intercourse) (Group B; 25 patients). | High-risk HPV patients |
| Lara-Cerrillo (2020)^44^ | Varicocelectomy | Microsurgical varicocelectomy: subinguinal incision, the dissection of the spermatic cord and the dissection of the dilated veins under x20–30 microscopic magnification (Zeiss OPMI vario s88, Carl Zeiss Meditec AG, Germany) avoiding damage to the cremasteric and deferential arteries. Nine months after MV patients were evaluated for possible late complications. | Fertile: sperm donor. Infertile: varicocele patients |
| Li (2012)^45^ | Varicocelectomy | Microsurgical subinguinal varicocelectomy | Fertile: normozoospermic |
| Lipovac (2021)^46^ | Lifestyle, antioxidant | Males of the study group were instructed to take two daily oral capsules of PROfertilVR (Lenus Pharma GmbH, Vienna, Austria). The two capsules contain: L-carnitine (440 mg), L-arginine (250 mg), zinc (40 mg), vitamin E (120 mg), glutathione (80 mg), selenium (60 lg), coenzyme Q10 (15 mg), and folic acid (800 lg). The control group consisted of those patients who did not receive the active treatment (n=177), yet were instructed to engage in a healthy lifestyle, including a modification of their regular diet. | Subfertile: male partners of couples who had consulted the clinic. |
| Maghsoumi-Norouzabad (2021)^47^ | Antioxidant | Group A, who received daily doses of 4000 IU VD3 (cholecalciferol), and group B, who received identical placebo for 3 months. | Infertile: asthenozoospermic men (the motility of sperm <40% and rapid progressive sperm motility<32%) with vitamin D levels less than 30 ng/ml in the infertility clinic. |
| Martínez-Soto (2016)^48^ | Antioxidant | The placebo group participants received 1,500 mg/day of sunflower oil. The sunflower oil placebo contained the 18-carbon fatty acids, oleic acid and linoleic acid, but no fatty acids longer than 20 carbons and was selected as it minimally alters the fatty acid composition of the typical diet. The docosahexaenoic acid (DHA) group received 1,500 mg/day of DHA-enriched oil. This group of patients ingested 990 mg of DHA and 135 mg of eicosapentaenoic acid (EPA) per day. The DHA source is a fish derived triacylglyceride oil that contains a high level of DHA provided in gelatine capsules, each containing 330 mg of DHA (fatty acid composition of study capsules is shown in Table 4). Treatment duration was 10 weeks. | Infertile: undergoing evaluation for infertility. |
| Ménézo (2007)^49^ | Antioxidant | Daily oral antioxidant treatment consisting of vitamins C and E (400 mg each), β-carotene (18 mg), zinc (500 μmol) and selenium (1 μmol): this is the classic treatment given by andrologists (see Agarwal et al., 2004). | Patients who had at least two previous failures of IVF or ICSI were enrolled in the study. |
| Micic (2019)^50^ | Antioxidant | Proxeed Plus: 1,000 g LC, 0.5 g ALC, 0.725 g fumarate, l g fructose, 50 mg citric acid, 10 mg zinc, 20 mg coenzyme Q10, 50 µg selenium, 90 mg vitamin C, 200 µg folic acid and 1.5 µg vitamin B12. The placebo was made with the excipients (sucrose, silica [anti‐caking], lemon flavour, acesulfame K [E950, sweetener]) of the supplementation without the active substances. After 2 months of “wash‐out” (period without any therapy), treatment occurred for 6 months with Proxeed Plus (125 patients) or placebo (50 patients), followed by control semen analyses 3 and 6 months after therapy initiation (T3, T6). Patients received either test formulation, Proxeed Plus, or placebo (two times per day) in a randomised, double‐blind fashion. | Idiopathic oligoasthenozoospermia who failed to impregnate their partners (12 months). |
| Miñambres (2022)^51^ | Bariatric surgery | Seven patients (58.3%) underwent gastric bypass and five (41.7%) underwent sleeve gastrectomy. | Bariatric surgery candidates. |
| Mir (2018)^52^ | Lifestyle | The weight loss programme, based on a healthy diet and daily exercise, lasted approximately 12 weeks. | Men with BMI over 25 kg/m² who attended our infertility department for diagnostic purpose and weight loss centres interested in weight loss programme. |
| Mohammed (2015)^53^ | Varicocelectomy | Subinguinal varicocelectomy with loop magnification | Men with at least 1-year history of infertility, a palpable varicocele, oligo, astheno, or teratozoospermia were selected from our andrology clinic. Forty healthy fertile volunteers (control group) were also included in this prospective study. |
| Moskovtsev (2010)^54^ | Antioxidant | 3 months. The course of treatment included either Fertile One (Coast Reproductive, Inc., San Diego, CA), which is a combination of vitamins (C, E, B6,B12, and folic acid), herbs (ginseng root and garlic), and supplements (ferulic acid, coenzyme Q10, L-carnitine, zinc, and selenium) or daily vitamins (C, 500 mg; E, 100 IU; and folic acid, 1 mg) supplemented with 20 mg of zinc and 200 mg of selenium. | Men who had persistently high DNA damage (>30%) |
| Moskovtsev (2009)^55^ | Antioxidant | All patients were instructed to take a three month course of oral antioxidant treatment, either Fertile Ones, a combination of vitamins (C, E, B6, B12, folic acid), herbs (ginseng root, garlic) and supplements (ferulic acid, coenzyme Q10, L-carnitine, zinc, selenium) or daily vitamins (C 500 mg, E 100 IUS, folic acid 1 mg) supplemented with 20mg of zinc and 200 mcgs selenium. Antibiotic treatment included either a two-week course of Ciprofloxacin (500mg twice a day or extended-release (XL) 1000mg daily) or Amoxicillin 500mg three times a day. In case of ureaplasma infection, Zithromax (Azithromycin, Z-pack) 250mg for 5 days was prescribed. | Group I: idiopathic infertility (n=24), group II: clinically significant varicocele. Bacteriospermia were present in 14 patients (16%), including 7 patients with varicocele, who received oral antioxidants and antibiotics according to the results of semen culture (Group III). |
| Nazari (2020)^56^ | Antioxidant | In this step all patients were given 400 IU synthetic vitamin E (α‑tocopherol) daily in combination with Zinc (10 mg) for 90 days and after this for the second time semen samples were collected and analyzed. | Partners of women suffering from recurrent pregnancy losses. |
| Negri (2017)^57^ | Antioxidant | Of these men, 15 had received no medical or surgical treatment and were used as a control group. The remaining 103 had received some oral antioxidant supplementation (12 different combinations of both hydrophilic and lipophilic antioxidants), whose composition is shown in Table 2. Fifty-five were treated with SOD-based antioxidant FertiPlus® SOD, whose formulation contains ORISOD®, Extramel®, α-lipoic acid, glutathione, folic acid, zinc, and vitamins B2, B3, B6, B12. FertiPlus® SOD is a balanced combination of enzymatic and non-enzymatic antioxidants (SOD micro encapsulated [Extramel®]), alpha lipoic acid, glutathione (low dose), zinc, B vitamins, a micronutrient complex (ORISOD®) containing substances of plant origin, hydroxytyrosol, and carnosol, identified as substances able to activate the antioxidant system and detoxify intracellular endogenous NRF-2 (nuclear transcription factor-erythroid 2) | Male partners of infertile couples |
| Ni (2014)^58^ | Varicocelectomy | Surgery was performed by retroperitoneal approach ligation of the dilated internal spermatic veins. Briefly, with the patient under local or general anesthesia an approximately 6 cm transverse skin incision was made above the internal inguinal ring. After entering the retroperitoneum the dilated internal spermatic veins were dissected, doubly ligated with 4-zero silk ligatures and transected under an operating microscope at x5 to x10 magnification. Papaverine solution was used to irrigate as needed to prevent vessel spasm. The incision was then closed in layers. | Subfertile males with oligozoospermia (less than 15 106 ⁄ml) and/or asthenozoospermia (less than 32% progressive motility). |
| Ni (2016)^59^ | Varicocelectomy | Surgery was performed by retroperitoneal approach ligation of the dilated internal spermatic veins. Briefly, with the patient under anesthesia, an approximately 6-cm transverse skin incision was made above the internal inguinal ring. After entering the retroperitoneum, the dilated internal spermatic veins were dissected, doubly ligated with 4-zero silk ligatures, and transected under an operating microscope at 910 magnification. Papaverine solution was used to irrigate as needed to prevent vessel spasm. The incision was then closed in layers. | Infertile male patients with varicocele consulting in the outpatient department of Andrology in Renji Hospital for infertility. Normozoospermic healthy donors with proven fertility (n = 25) were recruited as controls. |
| Ozer (2020)^60^ | Antioxidant | Group A received 500 mg daily vitamin C (EsterC plus, Solgar, USA), 400 IU daily vitamin E (Evicap fort, Kocak Farma, Turkey) and 600 mg daily N-acetylcysteine (NAC) (Assist plus, Bilim, Turkey). Group B received 100 mcg daily selenium (Selenium, Solgar, USA) and 100 mg daily coenzyme Q10 (coQ10) (Coenzyme Q-10, Solgar, USA) in addition to vitamin C, vitamin E and NAC. Group C received commercial multiantioxidant supplement (NeoFortil M, Tani Pharma, Turkey) in addition to vitamin C, vitamin E and NAC. Group D received another commercial multiantioxidant supplement (Promotil men, Centax Pharma, Turkey) in addition to vitamin C, vitamin E and NAC. The multiantioxidant supplement used in Group C (NeoFortil M) contains a daily dose of 600 mg L-Carnitine, 250 mg L-Arginine, 120 mg vitamin C, 72 mg vitamin E, 15 mg coQ10, 60 mcg selenium, 40 mg zinc sulfate and 800 mcg folic acid and the other one used in Group D (Promotil men) contains a daily dose of 2000 mg L-carnitine, 500 mg L-arginine, 200 mg vitamin C, 120 mg vitamin E, 100 mg coQ10, 60 mcg selenium, 60 mg magnesium, 15 mg zinc sulfate, 400 mcg folic acid, 2 mg vitamin B6, 6 mcg vitamin B12, 10 mcg vitamin D, 1 mg vitamin A and 2 mg beta-carotene. | - |
| Özgök Kangal (2021)^61^ | Hyperbaric oxygen | The routine HBOT protocol is 100% oxygen at a pressure of 2.4 atmospheric absolute (ATA) for 120 minutes. Patients were also given routine antioxidant medical therapy (ZincTab 100 mg, Selenium 100 micrograms, CoEnzyme Q10 100 mg) for six months. Microsurgical varicocelectomy was performed on patients diagnosed with varicocele. | Infertile: history of infertility for at least one year |
| Palomba (2011)^62^ | FSH | All subjects received hpFSH (IBSA Institut Biochimique, Italy). In particular, a dose of 150 IU hpFSH (2 vials) were administered im on alternate days for 3 months. | Idiopathic male factor infertility was diagnosed at study entry by subnormal semen parameter findings according to the World Health Organization (WHO) criteria (14, 15) on at least 3 separate occasions. |
| Pelliccione (2011)^63^ | Lifestyle | Upon reaching Kathmandu, participants underwent 13 days of an acclimatization trekking to reach Manaslu base camp at 5,000 m and subsequently spent 22 days at HA (between 5,900 and 6,400 m) without oxygen supplementation, during which they were engaged in a regular trekking activity. Participants returned to Kathmandu in 8 days trekking and were immediately submitted to blood sampling (intermediate point) before flying to Italy. | Trained and experienced mountaineers |
| Pini (2020)^64^ | Antioxidant | 1800 mg freeze-dried acai pulp per day. Patients were treated for a minimum of 74 days to ensure that the treatment period captured at least one new generation of spermatozoa, given that spermatogenesis takes approximately 74 days in humans. Chemical analysis of the lot by the manufacturer reported a total polyphenol content of 6618 mg Gallic acid equivalent (GAE)/100 g, oxygen radical absorbance capacity (ORAC) of 208,628 µmol Trolox equivalent (TE)/100 g and negligible microbial contamination. | Infertile: patients presenting for infertility treatment. |
| Sakamoto (2008)^65^ | Varicocelectomy | All men with varicocele had a unilateral or bilateral microsurgical subinguinal varicocelectomy. | Infertile: seeking infertility treatment. |
| Salas-Huetos (2018)^66^ | Lifestyle | one group was fed the usual Western-style diet enriched with 60 g of a mixture of nuts/d (30 g of walnuts, 15 g of almonds, and 15 g of hazelnuts) (nut group), and the other was fed the usual Western-style diet avoiding nuts (control group). | General population: healthy men |
| Salehi (2019)^67^ | Antioxidant | The antioxidant supplementation contained the following active ingredients: 50 mg vitamin E (d-α-tocopherol acetate, Daro pakhsh, Iran); 500 mg vitamin C (ascorbic acid, Daro pakhsh, Iran); and 100 mg Q 10 (Daro pakhsh, Iran), which was used daily for three months. | Infertile: patients of andrology centre. |
| Samavat (2018)^68^ | Bariatric surgery | All operated patients underwent Laparoscopic Roux-en-Y astric Bypass (LRYGBP) as bariatric surgery technique; non-operated patients experienced only a modest change in lifestyle and diet regimen while in the waiting list for bariatric surgery. | Morbidly obese patients (BMI = 46.2 ± 6.7) |
| Scaruffi (2021)^69^ | Antioxidant | Two Gametogen® tablets contained myo-inositol (1000 mg), alpha-lipoic acid (800 mg), folic acid (400 mg), coenzyme Q10 (200 mg), zinc (15 mg), selenium (83 µg), and vitamins B2 (2.8 mg), B6 (2.8 mg), and B12 (5 µg). | History of a homolog ICSI attempt using fresh oocytes and ejaculated sperm with unexplained <60% fertilization rate |
| Simoni (2016)^70^ | FSH | All subjects included in the study underwent a treatment phase of 12 weeks, receiving recombinant FSH therapy (follitropin alpha: Gonal-f) 150 IU s.c. every other day. The follow-up phase continued for a further 12 weeks after the end of the treatment. | Infertile: minimum 1 year. |
| Smit (2013)^71^ | Varicocelectomy | Left high inguinal spermatic vein ligation was performed in 36 men and microsurgical varicocelectomy was done in 8. All bilateral varicoceles were treated with bilateral high inguinal ligation. | Infertile: at least a 1-year history of infertility |
| Steiner (2020)^72^ | Antioxidant | Men received a placebo or an antioxidant formulation containing 500 mg of vitamin C (ascorbic acid), 400 mg of vitamin E (d-a tocopheryl), 0.20 mg of selenium (L-selenomethionine), 1,000 mg of L-carnitine, 20 mg of zinc, 1,000 mg of folic acid, 10 mg of lycopene, and 2,000 IU of vitamin D daily (IND #125753) for at least 3 months and up to 6 months. | Infertile: at least 12 months of infertility. |
| Stenqvist (2018)^73^ | Antioxidant | Antioxidant treatment with a commercial fertility supplement containing vitamins (vitamin C 30 mg, vitamin E 5 mg and vitamin B12 0.5 lg), antioxidants (l-carnitine 750 mg, coenzyme Q10 10 mg and folic acid 100 lg) and oligoelements (zinc 5 mg and selenium 25 lg) with maltodextrin, calcium carbonate, citric acid, steviol glycoside, flavours, beta-carotene and silicon dioxide or placebo (maltodextrin, calcium carbonate, citric acid, steviol glycoside, flavours, beta-carotene and silicon dioxide) by a simple, unrestricted procedure. Both were administered orally twice per day. | Infertile: at least one year of unsuccessful attempt to achieve pregnancy. |
| Sun (2018)^74^ | Varicocelectomy | Microsurgical subinguinal varicocelectomy: a 3–4 cm incision was made inferior to the external inguinal ring. The spermatic cord was isolated, and the veins were ligated, while the arteries, lymphatics and the vas deferens were isolated under a surgical microscope (Carl-Zeiss, Jena, Germany). The skin was closed using 4-0 vicryl sutures after then. | Infertility duration > 1 year with unprotected sexual intercourse and impaired semen quality (sperm concentration < 20 million/ml, progressively motile sperm < 50% or sperm morphology < 4%, alone or in combination. |
| Telli (2015)^75^ | Varicocelectomy | All varicoceles were treated with inguinal spermatic vein ligation. | Infertile: at least 1-year history of infertility. |
| Vahidi (2021)^76^ | Antioxidant | 3 months (folic acid 1 mg/day, once daily selenium plus-EuRho® Vital Selen Plus capsule, Euro OTC Pharma GmbH, and once daily /250 mg L-carnitine ). | Infertility: not defined |
| Verdi (2020)^77^ | FSH | Patients received subcutaneous recombinant FSH (Gonal-f) treatment, 75 IU every other day, starting from Visit 1, for three months, three times a week. | Infertile: referred to highly specialized infertility treatment center due to at least 2 years of infertility. (oligozoospermic) |
| Wald (2021)^78^ | Varicocelectomy | Both surgeons performed all varicocelectomies using a surgical microscope via a subinguinal incision. Dissection was completed down to the spermatic cord which was delivered and secured with a penrose drain. Microsurgical intraoperative Doppler was used for assistance in isolating arterial blood supply within the spermatic cord and the vas deferens was protected. Lymphatics were spared when possible to reduce hydrocele formation. One surgeon (method 1) ligated any dilated vasal veins > 2.5 mm, while normal vasal veins were preserved. This surgeon (method 1) also performed testicular delivery with division of external spermatic cord and gubernacular veins. The other surgeon (method 2) only ligated vasal veins in the case of a recurrence and did not perform testicular delivery. He did divide subinguinal external spermatic cord veins when found. | Men were defined as subfertile if unable to conceive and/or having a semen parameter abnormality according to the WHO5 manual semen analysis. |
| Wang (2012)^79^ | Varicocelectomy | Local anesthesia, horizontal transverse incision at the midpoint connecting the left anterior superior iliac and the pubic symphysis. Cut the skin, bluntly separate subcutaneous tissue to the external oblique aponeurosis, cut external oblique aponeurosis along the fiber direction, bluntly separate the internal oblique muscle, transverse abdominal muscle and fascia, enter the extraperitoneal space, push the extraperitoneal fat medially to find and expose spermatic vessels on the peritoneal surface. Open the spermatic sheath, peel off the fat, dissociate and separate the internal spermatic artery and vein, double ligate the internal spermatic vein bundle, cut off the distal testis end of the vein bundle, squeeze the left scrotum and inguinal canal, and drain the vein. After the residual blood in the proximal section of the bundle is drained, the cut end is double ligated, and then the two cut ends are ligated to connect them together to maintain the continuity of the venous bundle. The vas deferense and spermatic artery were checked for damage and the incision was closed after no active bleeding. | Infertile: asthenozoospermic. |
| Werthman (2008)^80^ | Varicocelectomy | Mini-incision microsurgical inguinal varicocele repair in the outpatient setting performed by a single surgeon. | Infertile: more than a year of infertility and had at least one abnormal parameter on their semen analysis. |
| Yakovlev (2022)^81^ | Antioxidant | Patients of the first group received a course of Prostatilen® AC, containing prostate extract of 0.03g + rectal zinc arginyl-glycinate 0.18g once a day for 10 day. The control group received a rectal placebo (liquid paraffin 0.06g, solid Vitepsol fat) once a day for 10 days. After 21 days , both groups' patients received a 10-day course of Prostatilen® AC, | Infertile: at least 1-year history of infertility. |
| Yuan (2021)^82^ | Antioxidant | In total, 15 g of Peijingsu was administered to the patients twice daily for 15 days. | Infertility: not defined |
| Zaazaa (2018)^83^ | Varicocelectomy, mast cell stabilizer | These patients were randomized into three equal treatment arms; those that underwent microsurgical subinguinal varicocelectomy, patients on 1 mg oral ketotifen twice daily for 3 months, and patients that underwent varicocelectomy and post-operative oral MCs stabilizer for 3 months. | Infertility: not defined |
| Zhang (2022)^84^ | Antioxidant | The treatment was administered as follows: the idiopathic male infertility group was treated with vitamin C (0.1 g three times a day), vitamin E (0.1 g three times a day), and Co Q10 (10 mg three times a day) for 3 months and followed up for 6 months. | Infertile: his spouse did not conceive without taking any contraceptive measures after more than 1 year of cohabitation. Controls: healthy. |
| Zhao (2019)^85^ | hCG | Patients (n = 158) in treatment group received intramuscular injections of 2000 IU hCG twice a week in combination with 150 IU hMG three times a week for 3 months, while the remaining patients (n = 158) in placebo group received intramuscular injections of physiological saline solution for 3 months. | Infertile: minimum 1 year of infertility. |
| Zini (2011)^86^ | Varicocelectomy | Microsurgical varicocelectomy | Infertile: one year or more of infertility. Couples in whom the wife had tubal obstruction or ovulatory failure were not included. |

**Supplementary Table 5.** Risk of bias assessment using the ROBINS-I tool

| **Author (year)** | **Confounding** | **Selection** | **Classification of intervention** | **Deviation from intended intervention** | **Missing data** | **Measurement of outcome** | **Selection of reported results** | **Overall** |
| --- | --- | --- | --- | --- | --- | --- | --- | --- |
| Abbasi (2021)^2^ | Serious | Low | Low | Low | Moderate | Low | Low | Serious |
| Alhathal (2016)^9^ | Serious | Low | Low | Low | Low | Low | Low | Serious |
| Boeri (2022)^12^ | Serious | Serious | Moderate | Low | Critical | Low | Low | Critical |
| Condorelli (2021)^17^ | Moderate | Serious | Low | Low | Moderate | Low | Low | Serious |
| Esfahani (2010)^23^ | Serious | Low | Low | Low | Moderate | Low | Low | Serious |
| Fathi (2021)^24^ | Serious | Low | Low | Low | Low | Low | Low | Serious |
| Gallegos (2008)^27^ | Serious | Low | Low | Moderate/serious | Low | Serious | Low | Serious |
| Garolla (2017)^28^ | Serious | Low | Low | Low | Low | Low | Low | Serious |
| Humaidan (2021)^35^ | Serious | Low | Low | Moderate/serious | Low | Low | Low | Critical |
| Lara-Cerrillo (2020)^44^ | Serious | Low | Low | Low | Low | Low | Low | Serious |
| Li (2012)^45^ | Moderate | Low | Low | Low | Low | Low | Low | Moderate |
| Lipovac (2021)^46^ | Serious | Low | Low | Low | Low | Low | Low | Serious |
| Mohammed (2015)^53^ | Serious | Serious | Low | Low | Low | Low | Low | Serious |
| Negri (2017)^57^ | Low | Low | Low | Low | Low | Moderate | Low | Moderate |
| Ni (2014)^58^ | Low | Low | Low | Low | Low | Low | Low | Low |
| Ni (2016)^59^ | Low | Low | Low | Low | Low | Low | Low | Low |
| Samavat (2018)^68^ | Moderate | Low | Low | Low | Low | Low | Low | Moderate |
| Wang (2012)^79^ | Serious | Low | Low | Low | Low | Low | Low | Serious |
| Zhang (2022)^84^ | Moderate | Low | Low | Low | Low | Low | Low | Moderate |

**Supplementary Table 6.** Risk of bias assessment using the MINORS tool

| **Author (year)** | **A clearly stated aim** | **Inclusion of consecutive patients** | **Prospective collection of data** | **Endpoints appropriate to the aim of the study** | **Unbiased assessment of the study endpoint** | **Follow-up period appropriate to the aim of the study** | **Loss to follow up less than 5%** | **Prospective calculation of the study size** |
| --- | --- | --- | --- | --- | --- | --- | --- | --- |
| Abad (2013)^1^ | 2 | 0 | 0 | 2 | 1 | 2 | 0 | 0 |
| Abdelaziz (2015)^3^ | 2 | 2 | 2 | 2 | 0 | 2 | 2 | 0 |
| Abdelbaki (2017)^4^ | 2 | 2 | 2 | 2 | 1 | 2 | 2 | 0 |
| Afsin (2018)^5^ | 2 | 0 | 0 | 2 | 1 | 2 | 0 | 0 |
| Alahmar (2020)^6^ | 2 | 0 | 0 | 2 | 1 | 2 | 0 | 0 |
| Alahmar (2021)^7^ | 2 | 1 | 2 | 2 | 1 | 2 | 2 | 0 |
| Alahmar (2022)^8^ | 2 | 2 | 2 | 2 | 1 | 2 | 1 | 2 |
| Arafa (2020)^10^ | 2 | 2 | 2 | 2 | 1 | 2 | 1 | 0 |
| Baker (2013)^11^ | 2 | 0 | 0 | 2 | 1 | 1 | 0 | 0 |
| Cannarella (2021)^13^ | 2 | 0 | 0 | 2 | 1 | 2 | 0 | 0 |
| Colacurci (2018)^15^ | 2 | 2 | 2 | 2 | 1 | 2 | 2 | 2 |
| Dadfar (2021)^19^ | 2 | 0 | 0 | 2 | 1 | 2 | 0 | 2 |
| Dhawan (2018)^21^ | 2 | 1 | 2 | 1 | 1 | 0 | 0 | 0 |
| Elsedfy (2018)^22^ | 2 | 0 | 0 | 2 | 1 | 2 | 0 | 0 |
| Fornaro (2009)^25^ | 2 | 0 | 0 | 2 | 1 | 2 | 0 | 0 |
| Gabriel (2012)^26^ | 2 | 2 | 2 | 2 | 1 | 2 | 1 | 0 |
| Gallegos (2008)^27^ | 2 | 0 | 2 | 2 | 1 | 1 | 2 | 0 |
| Ghazi (2011)^29^ | 2 | 2 | 2 | 2 | 1 | 2 | 2 | 0 |
| Gual-Frau (2015)^31^ | 2 | 0 | 0 | 2 | 1 | 2 | 0 | 0 |
| Humaidan (2021)^35^ | 2 | 2 | 2 | 2 | 2 | 2 | 2 | 0 |
| Kavoussi (2021)^38^ | 2 | 0 | 0 | 2 | 2 | 2 | 0 | 0 |
| Kavoussi (2022)^39^ | 2 | 0 | 0 | 2 | 1 | 2 | 0 | 0 |
| Kooshesh (2020)^40^ | 2 | 2 | 2 | 2 | 1 | 2 | 2 | 0 |
| La Vignera (2012)^42^ | 2 | 0 | 0 | 2 | 1 | 2 | 0 | 0 |
| La Vignera (2022)^43^ | 2 | 0 | 0 | 2 | 1 | 2 | 0 | 0 |
| Ménézo (2007)^49^ | 2 | 0 | 0 | 2 | 1 | 2 | 0 | 0 |
| Miñambres (2022)^51^ | 2 | 0 | 0 | 2 | 1 | 2 | 0 | 2 |
| Mir (2018)^52^ | 2 | 0 | 0 | 2 | 1 | 2 | 0 | 2 |
| Moskovtsev (2010)^54^ | 2 | 1 | 2 | 2 | 1 | 2 | 1 | 0 |
| Moskovtsev (2009)^55^ | 2 | 0 | 0 | 2 | 1 | 2 | 0 | 0 |
| Nazari (2020)^56^ | 2 | 0 | 0 | 2 | 1 | 2 | 0 | 0 |
| Ozer (2020)^60^ | 2 | 0 | 0 | 2 | 1 | 2 | 0 | 1 |
| Özgök Kangal (2021)^61^ | 2 | 0 | 0 | 2 | 1 | 2 | 0 | 0 |
| Palomba (2011)^62^ | 2 | 1 | 2 | 2 | 1 | 2 | 1 | 0 |
| Pelliccione (2011)^63^ | 2 | 2 | 2 | 1 | 1 | 0 | 2 | 0 |
| Pini (2020)^64^ | 2 | 0 | 0 | 1 |  | 1 | 0 | 0 |
| Sakamoto (2008)^65^ | 2 | 0 | 0 | 2 | 1 | 2 | 0 | 0 |
| Salehi (2019)^67^ | 2 | 1 | 1 | 2 | 1 | 2 | 2 | 0 |
| Scaruffi (2021)^69^ | 2 | 1 | 2 | 2 | 1 | 2 | 0 | 0 |
| Simoni (2016)^70^ | 2 | 1 | 2 | 2 | 1 | 2 | 2 | 0 |
| Smit (2013)^71^ | 2 | 1 | 2 | 2 | 1 | 2 | 2 | 0 |
| Telli (2015)^75^ | 2 | 1 | 2 | 1 | 1 | 2 | 1 | 0 |
| Vahidi (2021)^76^ | 2 | 2 | 2 | 2 | 1 | 2 | 2 | 2 |
| Verdi (2020)^77^ | 2 | 0 | 0 | 2 | 0 | 2 | 0 | 0 |
| Wald (2021)^78^ | 2 | 0 | 0 | 2 | 1 | 2 | 0 | 0 |
| Werthman (2008)^80^ | 2 | 0 | 0 | 2 | 1 | 2 | 0 | 0 |
| Yuan (2021)^82^ | 2 | 0 | 0 | 1 | 1 | 0 | 0 | 0 |
| Zini (2011)^86^ | 2 | 2 | 2 | 2 | 1 | 2 | 2 | 2 |

**Supplementary Table 7.** Risk of bias assessment using the RoB2 tool

| Domains | 1 | 2 | 3 | 4 | 5 | Overall |
| --- | --- | --- | --- | --- | --- | --- |
| Capece (2017)^14^ | Low | High | Low | Low | Low | High |
| Colacurci (2012)^16^ | High | Some concern | Low | Low | Low | High |
| D'argent (2021)^18^ | Low | Low | Low | Low | Low | Low |
| Dadgar (2022)^20^ | Low | Low | Low | High | Low | High |
| Greco (2005)^30^ | Low | Some concern | Low | High | Low | High |
| Habibi (2022)^32^ | Low | Low | Low | Low | Low | Low |
| Hajizadeh Maleki (2020)^33^ | Low | Low | Low | Low | Low | Low |
| Huang (2020)^34^ | Low | Low | Low | Low | Low | Low |
| Jannatifar (2020)^36^ | Some concern | Some concern | Low | Low | Low | Some concern |
| Jannatifar (2021)^37^ | Some concern | Low | Low | Low | Low | Some concern |
| Kumar (2011)^41^ | Low | Low | Low | Low | Low | Low |
| Maghsoumi-Norouzabad (2021)^47^ | Low | Low | Low | Low | Low | Low |
| Martínez-Soto (2016)^48^ | Low | Low | Low | Low | Low | Low |
| Micic (2019)^50^ | Low | Low | Low | Low | Low | Low |
| Salas-Huetos (2018)^66^ | Low | Low | Low | Low | Low | Low |
| Steiner (2020)^72^ | Low | Low | Low | Low | Low | Low |
| Stenqvist (2018)^73^ | Low | Low | Low | Low | Low | Low |
| Sun (2018)^74^ | Low | Low | Low | Low | Low | Low |
| Yakovlev (2022)^81^ | Low | Low | Low | Low | Low | Low |
| Zaazaa (2018)^83^ | Low | Low | Low | Low | Low | Low |
| Zhao (2019)^85^ | Low | Low | Low | Low | Low | Low |

**Supplementary Table 8.** Grade assessment using grade-pro

| **Certainty assessment** | | | | | | | **№ of patients** | | **Effect** | | **Certainty** | **Importance** |
| --- | --- | --- | --- | --- | --- | --- | --- | --- | --- | --- | --- | --- |
| **№ of studies** | **Study design** | **Risk of bias** | **Inconsistency** | **Indirectness** | **Imprecision** | **Other considerations** | **[intervention]** | **[comparison]** | **Relative (95% CI)** | **Absolute (95% CI)** |  |  |
| **Sperm DNA fragmentation - antioxidant (follow-up: mean 3 months; assessed with: TUNEL, SCD, SCSA)** | | | | | | | | | | | | |
| 22 | observational studies | not serious | serious | serious | not serious | none | 1987 | 1987 | - | MD **4.51 % lower** (6.81 lower to 2.2 lower) | ⨁◯◯◯ Very low | IMPORTANT |
| **Sperm DNA fragmentation-varicocelectomy (follow-up: mean 3 months; assessed with: TUNEL, SCD, SCSA)** | | | | | | | | | | | | |
| 11 | observational studies | not serious | serious | not serious | not serious | dose response gradient | 607 | 607 | - | MD **100 % higher** (9.4 lower to 4.08 lower) | ⨁⨁◯◯ Low | IMPORTANT |
| **Sperm DNA fragmentation - FSH (follow-up: mean 3 months; assessed with: TUNEL, SCD, SCSA)** | | | | | | | | | | | | |
| 7 | observational studies | not serious | serious | serious | not serious | none | 417 | 417 | - | **0**  (0 to 0 ) | ⨁◯◯◯ Very low | IMPORTANT |
| **Sperm DNA fragmentation-lifestyle (follow-up: mean 3 months; assessed with: TUNEL, SCD, SCSA)** | | | | | | | | | | | | |
| 4 | observational studies | not serious | serious | serious | not serious | none | 519 | 519 | - | **0**  (0 to 0 ) | ⨁◯◯◯ Very low | NOT IMPORTANT |

**CI:** confidence interval; **MD:** mean difference

**Varicocelectomy:**


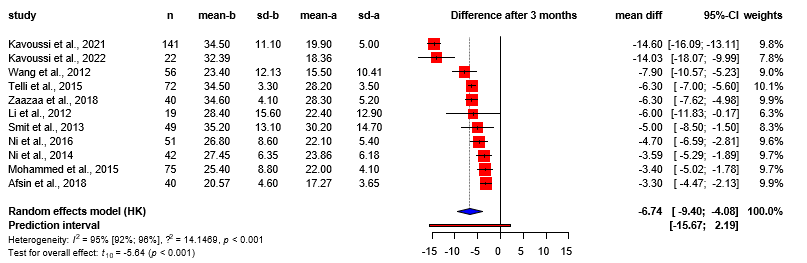


**
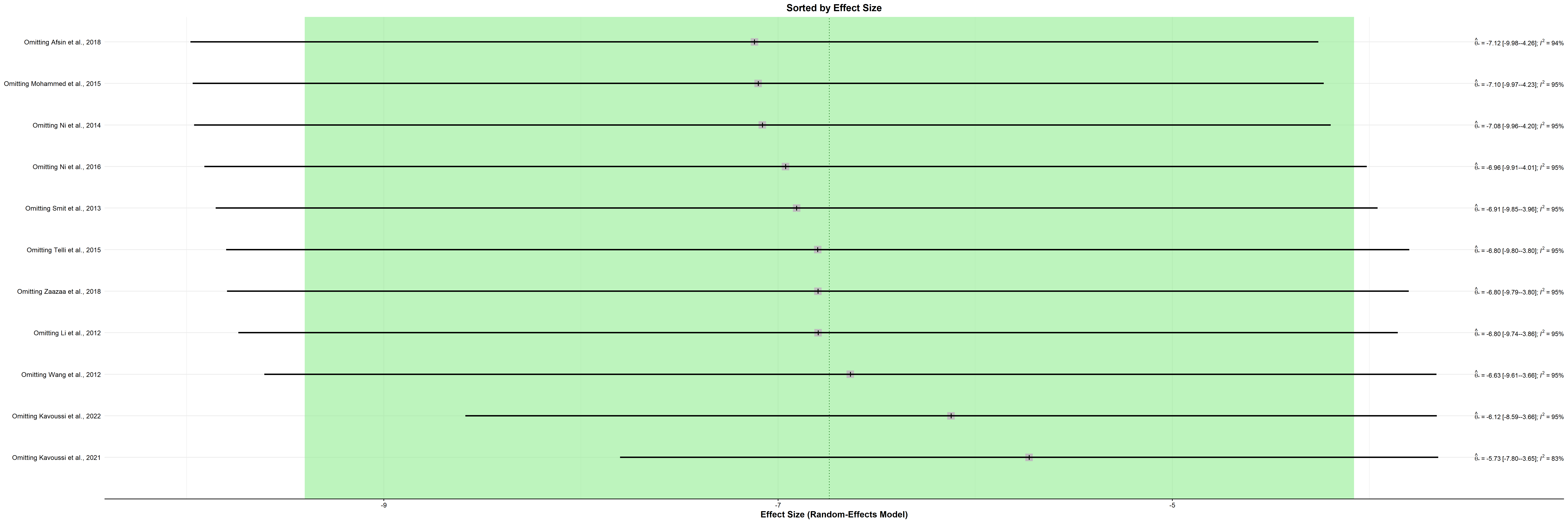
**

**
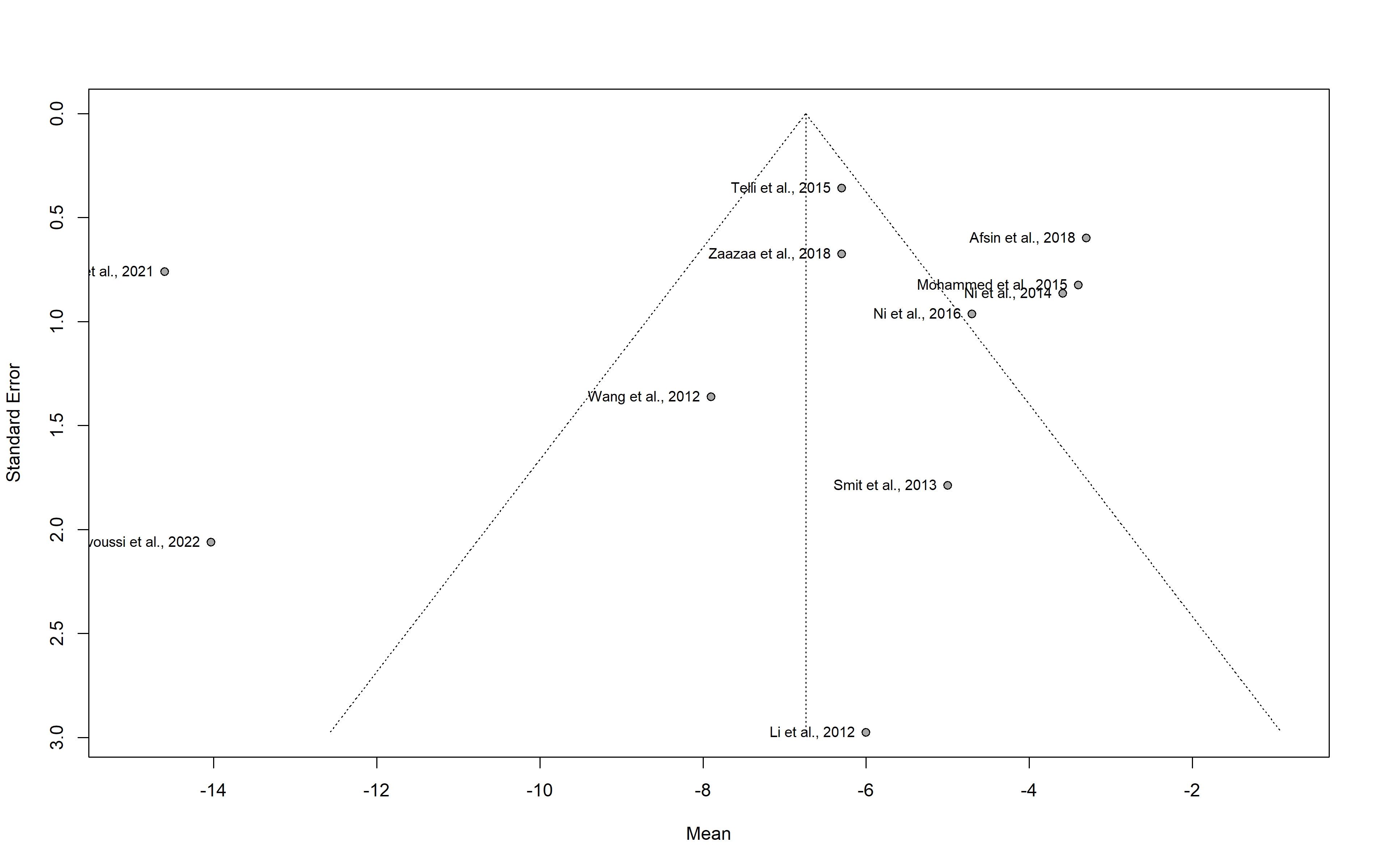
**

**Supplementary Figure 1/A:** Comparison of patients’ mean sperm DNA fragmentation values with a 95% confidence interval before and 3 months after varicocelectomy with an input correlation of 0.6 **B:** Corresponding leave-one-out analysis **C:** Corresponding funnel plot

**
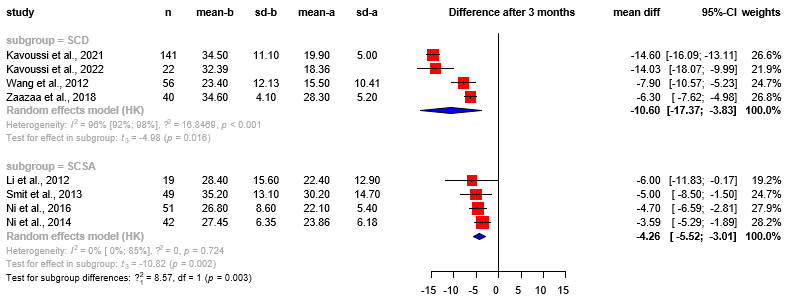
 Supplementary Figure 2:** Comparison of patients’ mean sperm DNA fragmentation values with a 95% confidence interval based on assay types before and 3 months after varicocelectomy with an input correlation of 0.6

**
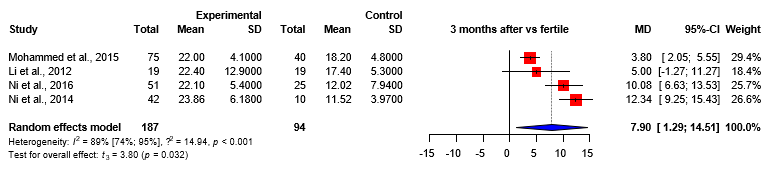
 Supplementary Figure 3:** Comparison of patients’ and fertile controls’ mean sperm DNA fragmentation values with a 95% confidence interval 3 months following patients’ varicocelectomies


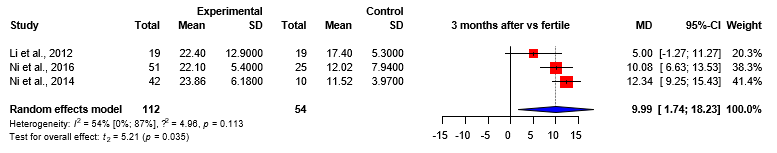
 **Supplementary Figure 4:** Comparison of patients’ and fertile controls’ mean sperm DNA fragmentation values with a 95% confidence interval via SCSA 3 months following patients’ varicocelectomies

**
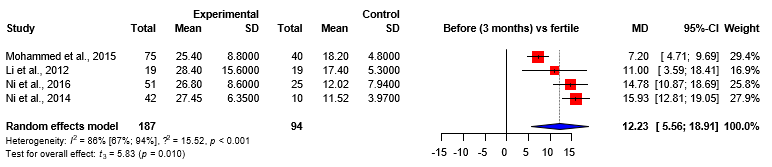
 Supplementary Figure 5:** Comparison of patients’ and fertile controls’ mean sperm DNA fragmentation values with a 95% confidence interval prior to patients’ varicocelectomies

**
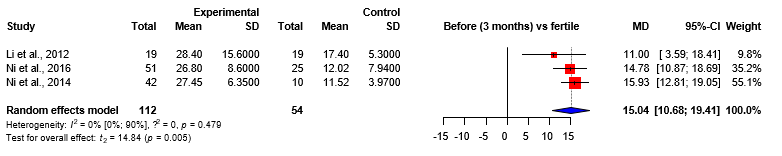
 Supplementary Figure 6:** Comparison of patients’ and fertile controls’ mean sperm DNA fragmentation values with a 95% confidence interval via SCSA prior to patients’ varicocelectomies


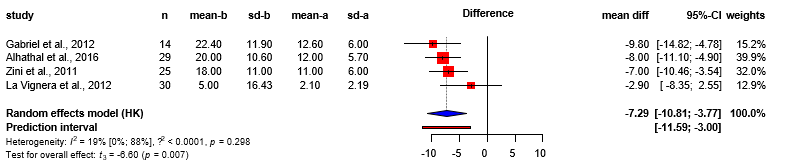
 **Supplementary Figure 7:** Comparison of patients’ mean sperm DNA fragmentation values with a 95% confidence interval before and 4 months after varicocelectomy with an input correlation of 0.6


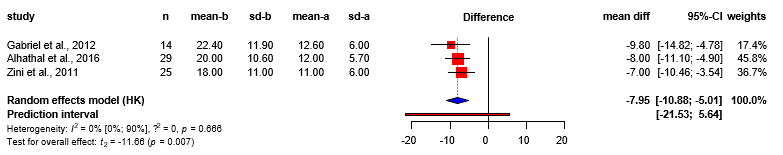
 **Supplementary Figure 8.:** Comparison of patients’ mean sperm DNA fragmentation values with a 95% confidence interval via SCSA before and 4 months after varicocelectomy with an input correlation of 0.6

**
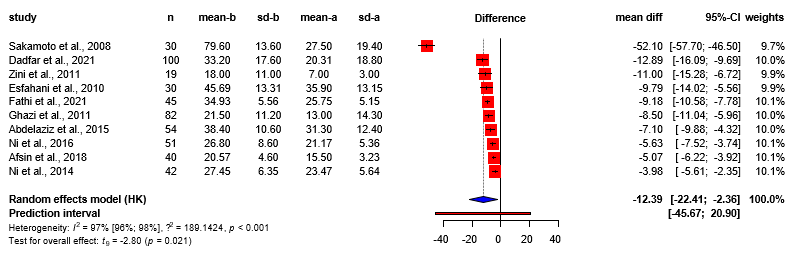
**

**
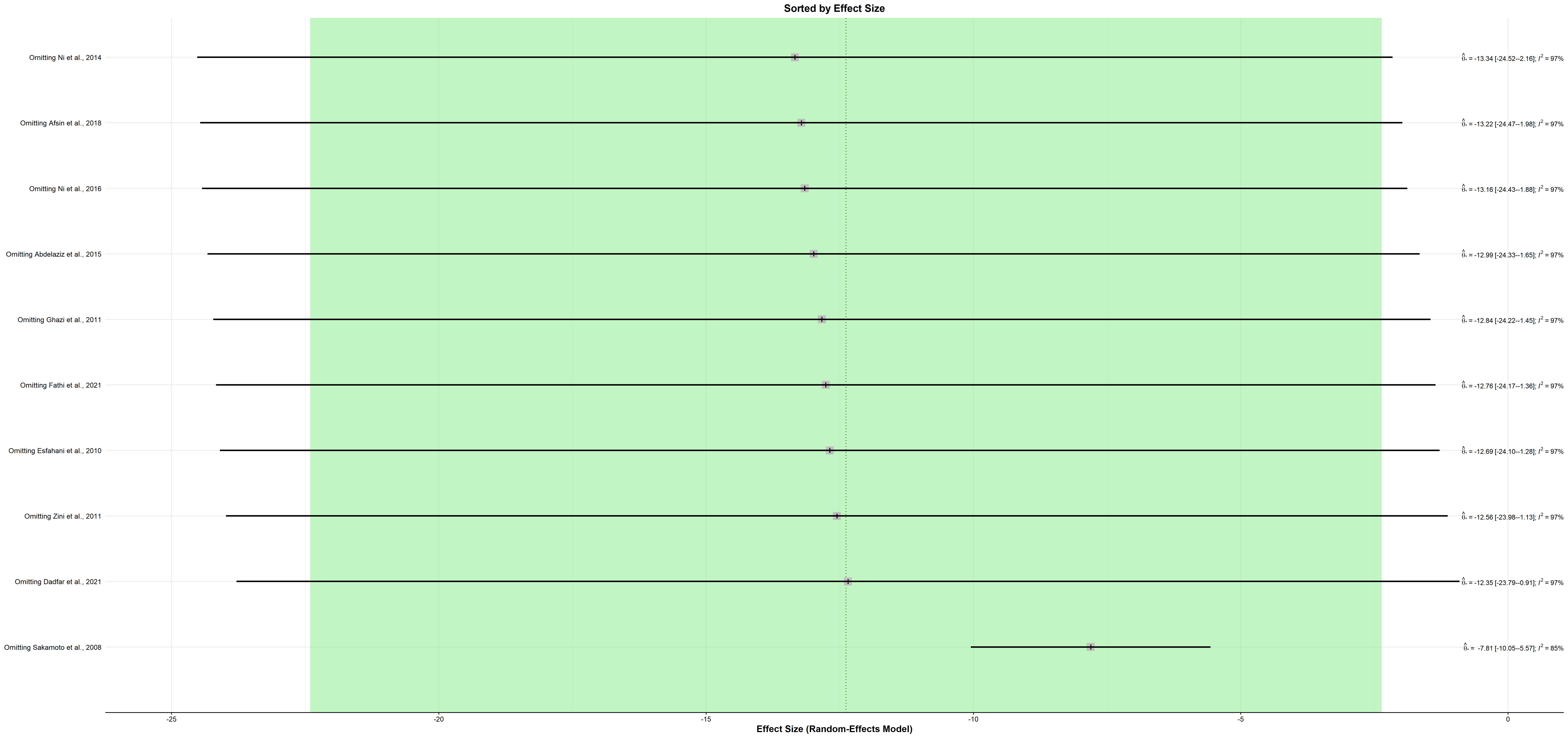
**

**
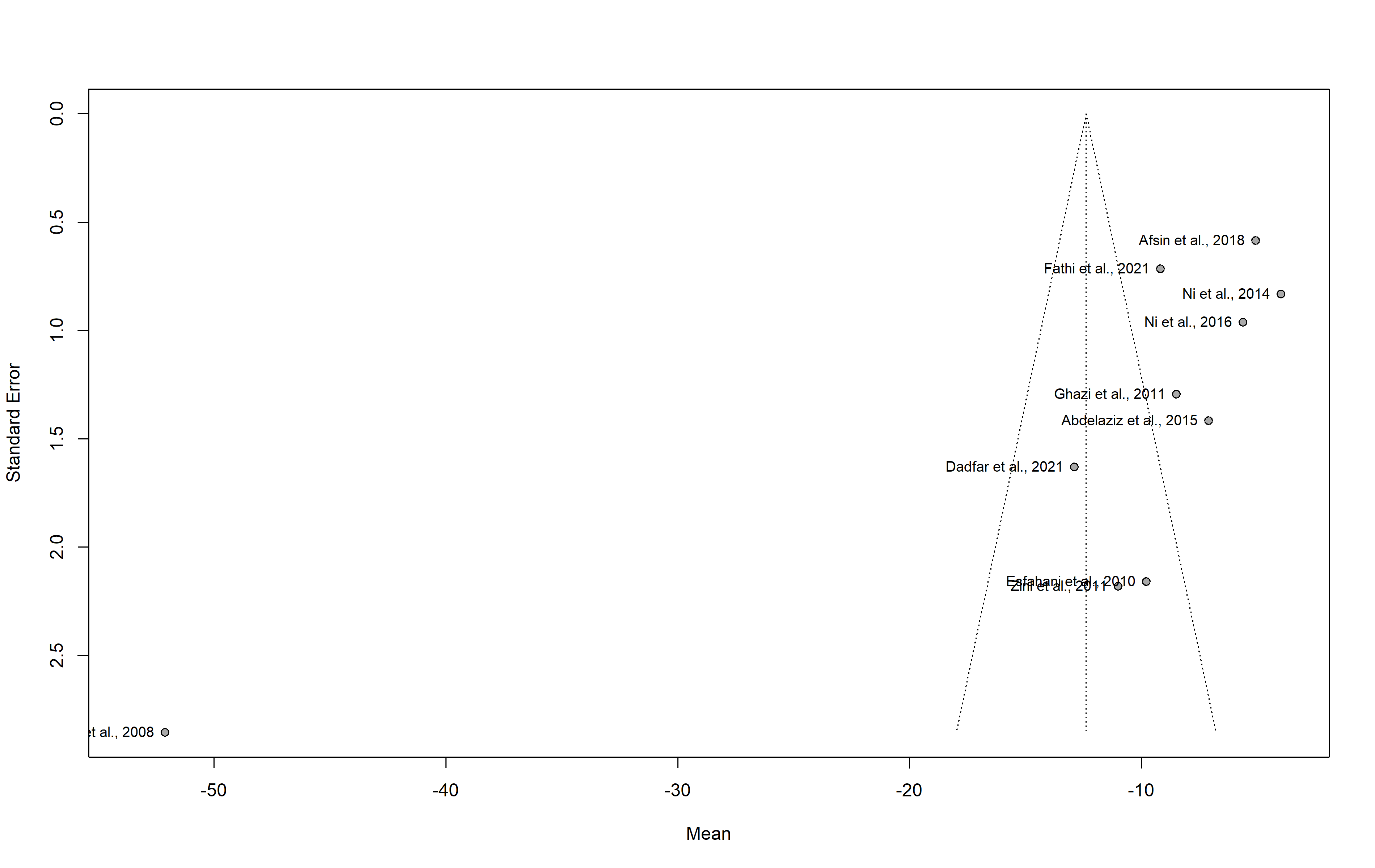
**

**Supplementary Figure 9:** Comparison of patients’ mean sperm DNA fragmentation values with a 95% confidence interval before and 6 months after varicocelectomy with an input correlation of 0.6 **B:** Corresponding leave-one-out analysis **C:** Corresponding funnel plot

**
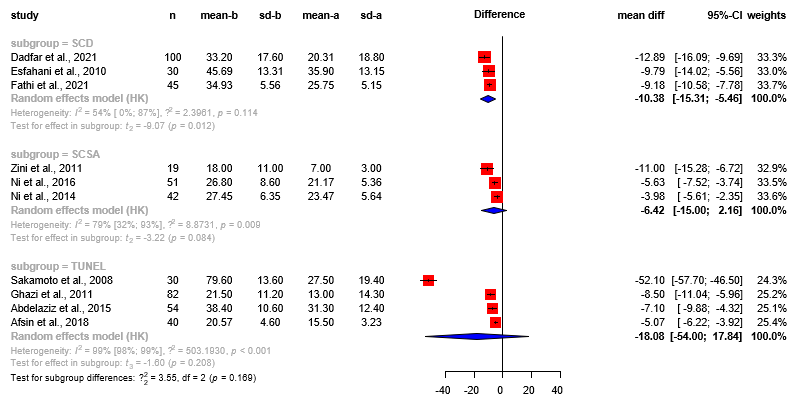
 Supplementary Figure 10:** Comparison of patients’ mean sperm DNA fragmentation values with a 95% confidence interval based on assay types before and 6 months after varicocelectomy with an input correlation of 0.6


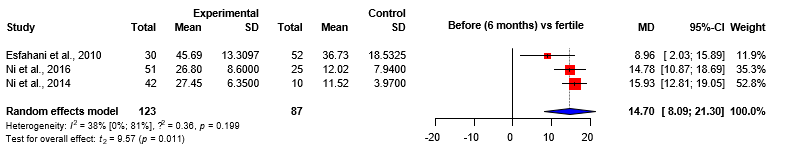
 **Supplementary Figure 11:** Comparison of patients’ and fertile controls’ mean sperm DNA fragmentation values with a 95% confidence interval prior to patients’ varicocelectomies


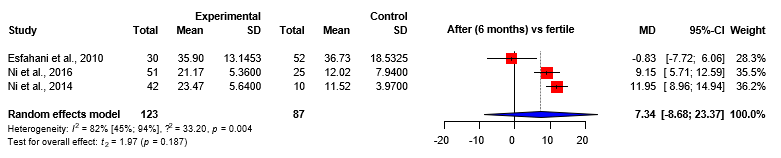
 **Supplementary Figure 12:** Comparison of patients’ and fertile controls’ mean sperm DNA fragmentation values with a 95% confidence interval 6 months following patients’ varicocelectomies

**
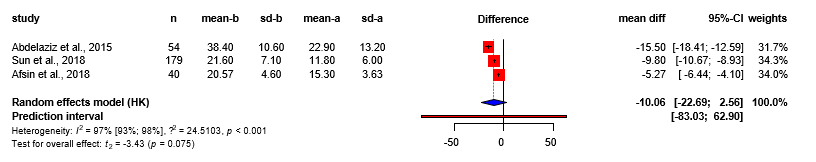
 Supplementary Figure 13:** Comparison of patients’ mean sperm DNA fragmentation values with a 95% confidence interval before and 12 months after varicocelectomy with an input correlation of 0.6


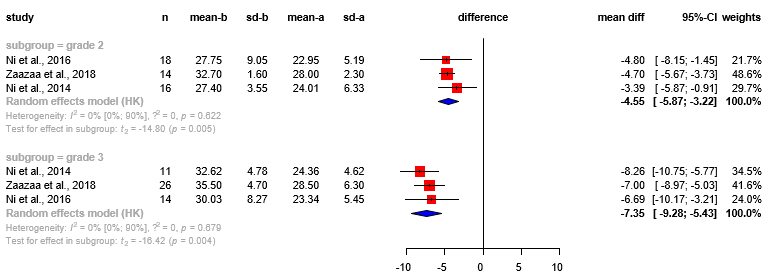
 **Supplementary Figure 14:** Comparison of patients’ mean sperm DNA fragmentation values with a 95% confidence interval based on varicocele grade with an input correlation of 0.6

**Antioxidant supplementation:**


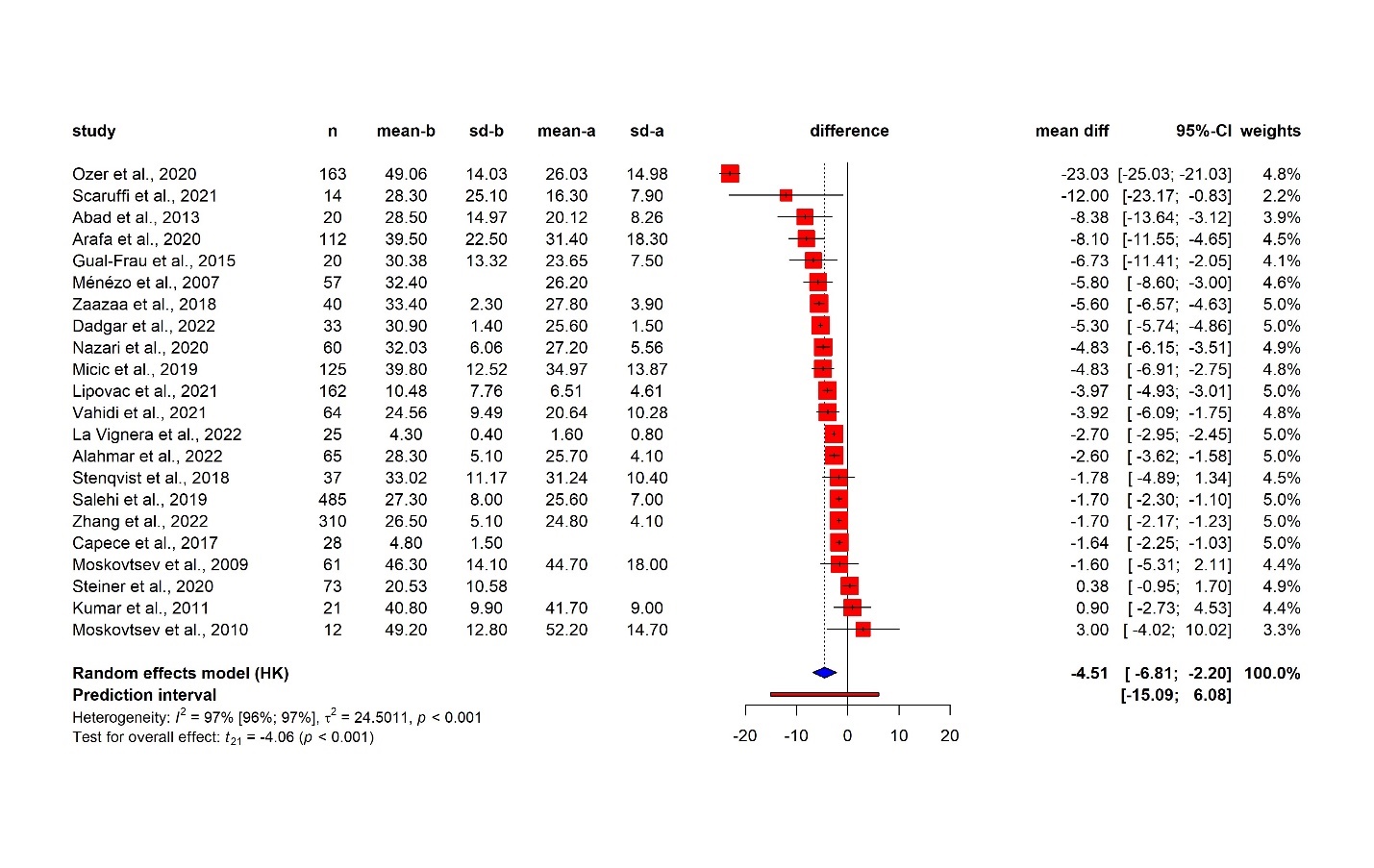


**
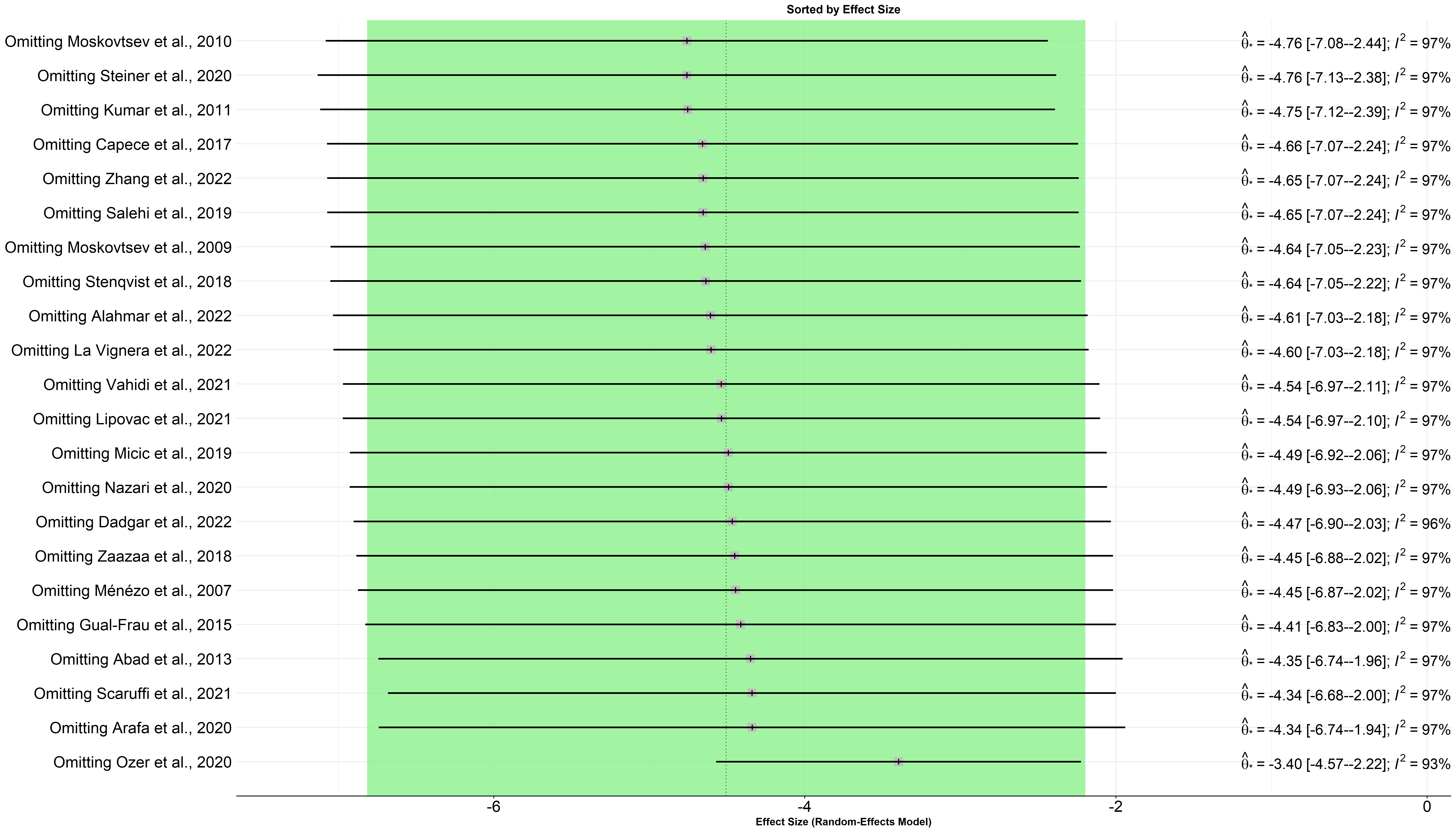
**

**
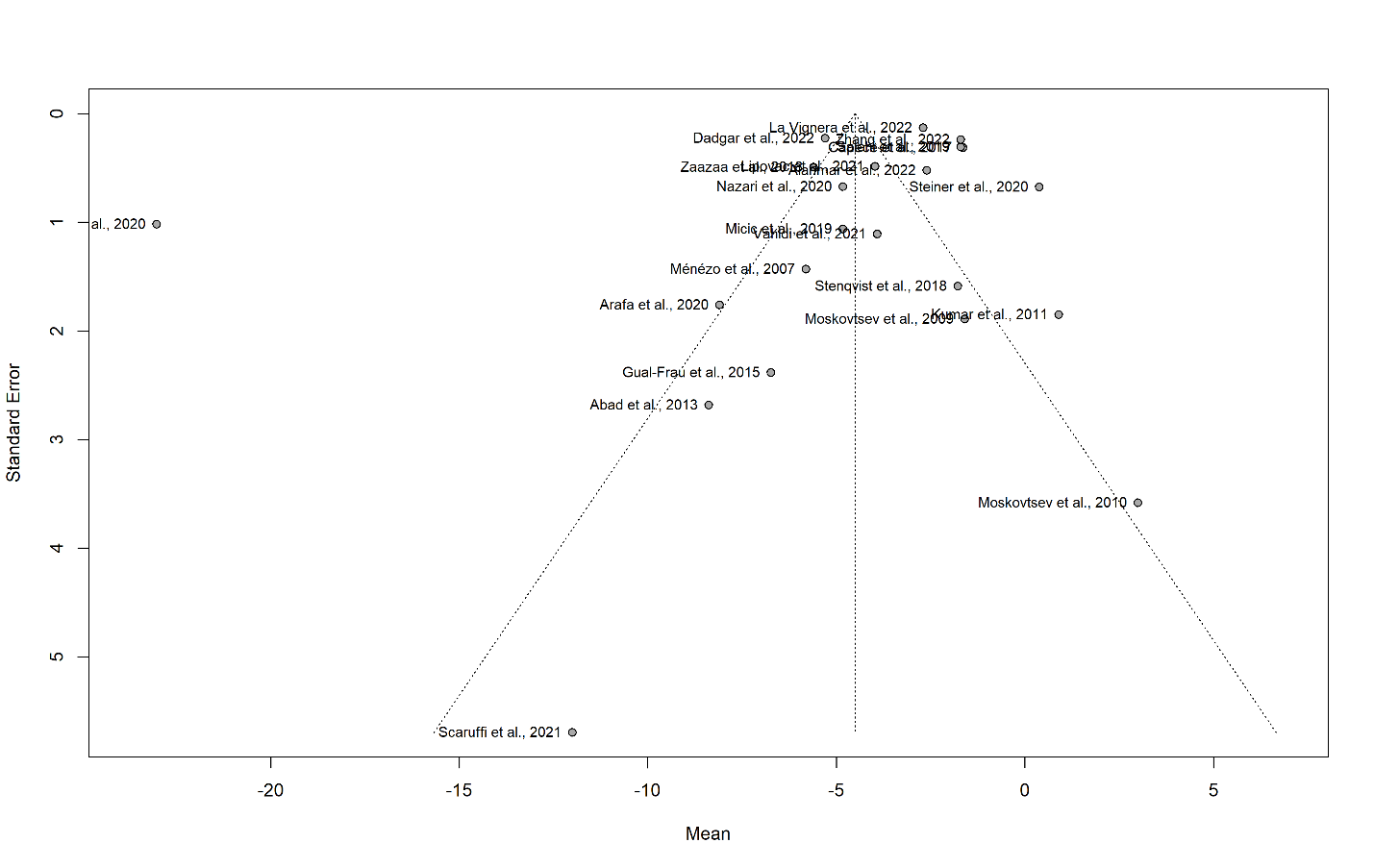
**

**Supplementary Figure 15:** Comparison of patients’ mean sperm DNA fragmentation values with a 95% confidence interval before and 3 months after combined antioxidant supplementation with an input correlation of 0.6 **B:** Corresponding leave-one-out analysis **C:** Corresponding funnel plot


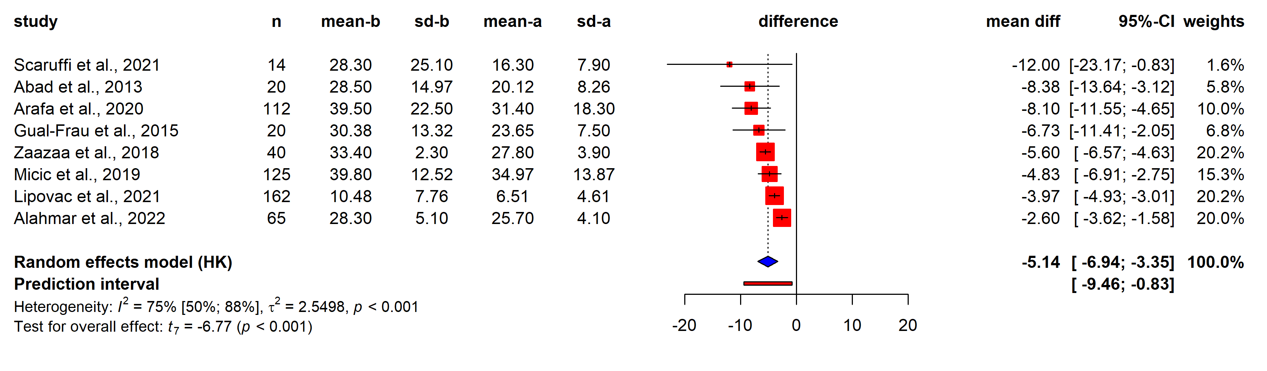
 **Supplementary Figure 16:** Comparison of patients’ mean sperm DNA fragmentation values with a 95% confidence interval measured via SCD before and 3 months after combined antioxidant supplementation with an input correlation of 0.6


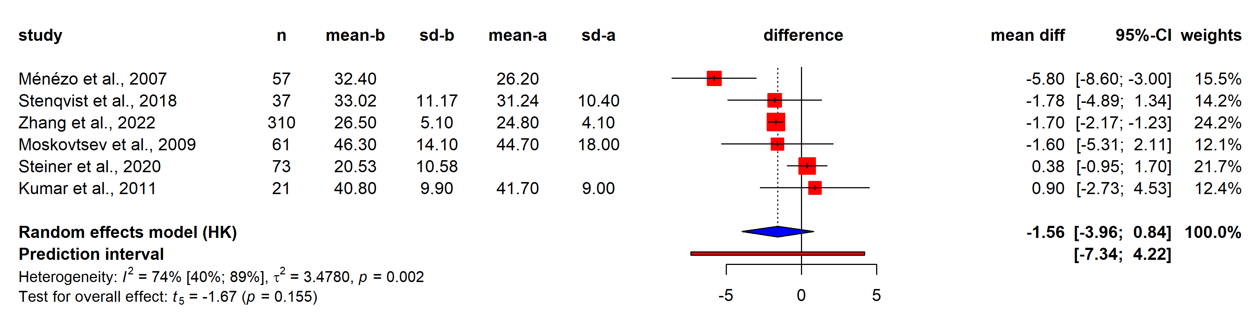
 **Supplementary Figure 17:** Comparison of patients’ mean sperm DNA fragmentation values with a 95% confidence interval measured via SCSA before and 3 months after combined antioxidant supplementation with an input correlation of 0.6


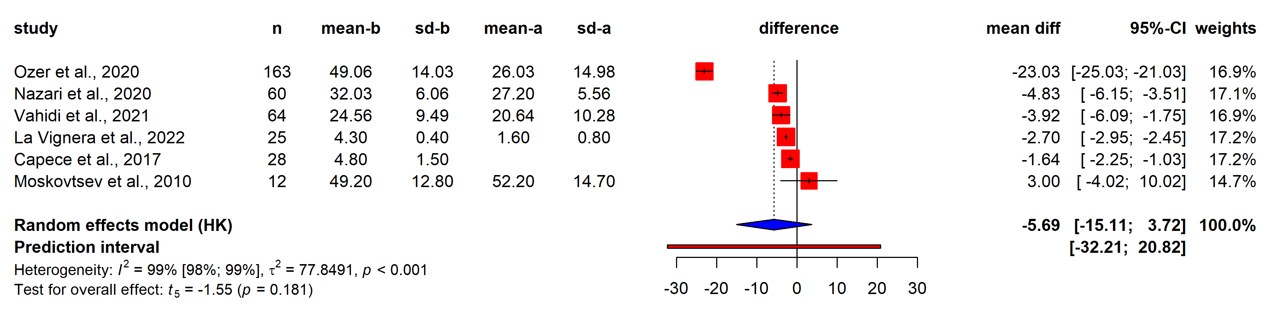
 **Supplementary Figure 18:** Comparison of patients’ mean sperm DNA fragmentation values with a 95% confidence interval measured via TUNEL assay before and 3 months after combined antioxidant supplementation with an input correlation of 0.6


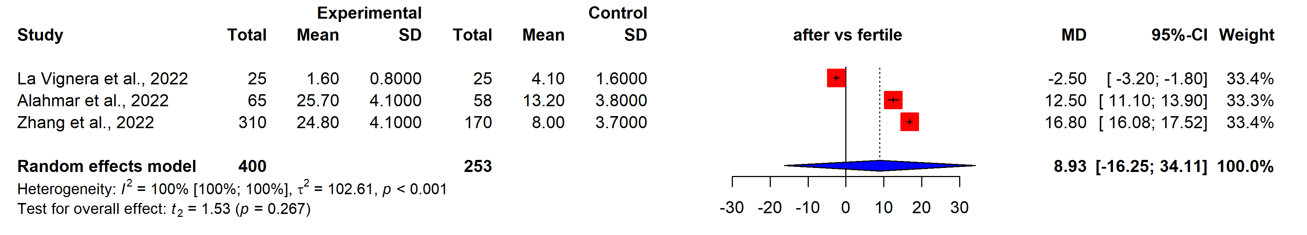
 **Supplementary Figure 19:** Comparison of patients’ and fertile controls’ mean sperm DNA fragmentation values with a 95% confidence interval 3 months following patients’ combined antioxidant supplementations

**
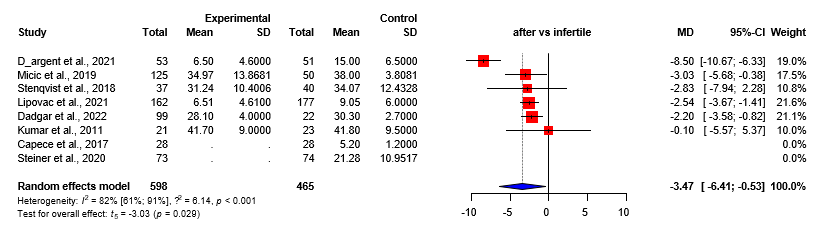
 Supplementary Figure 20:** Comparison of patients’ and infertile controls’ mean sperm DNA fragmentation values with a 95% confidence interval 3 months following patients’ combined antioxidant supplementations

**
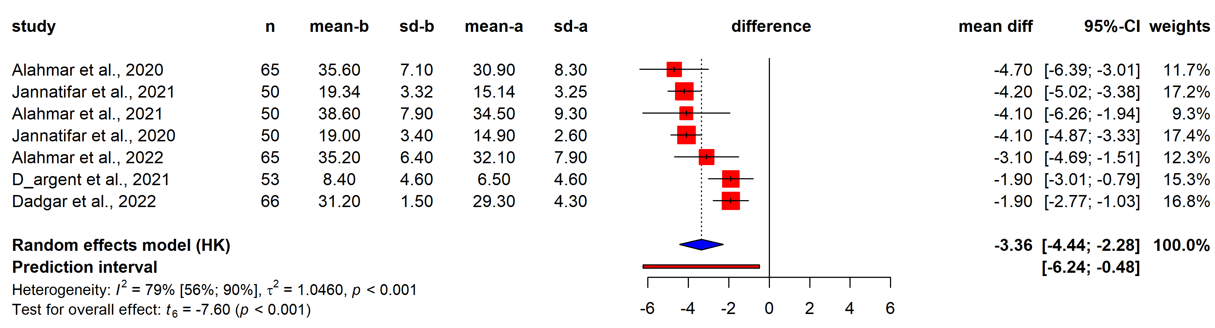
 Supplementary Figure 21:** Comparison of patients’ mean sperm DNA fragmentation values with a 95% confidence interval before and 3 months after a single type of antioxidant supplementation with an input correlation of 0.6


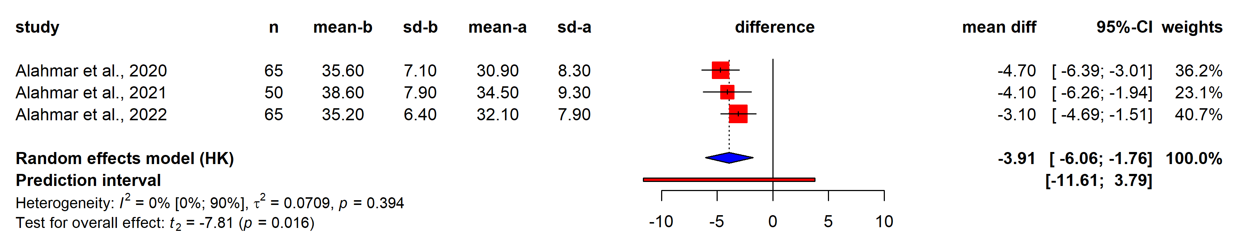
 **Supplementary Figure 22:** Comparison of patients’ mean sperm DNA fragmentation values with a 95% confidence interval measured via SCD before and 3 months after a single type of antioxidant supplementation with an input correlation of 0.6


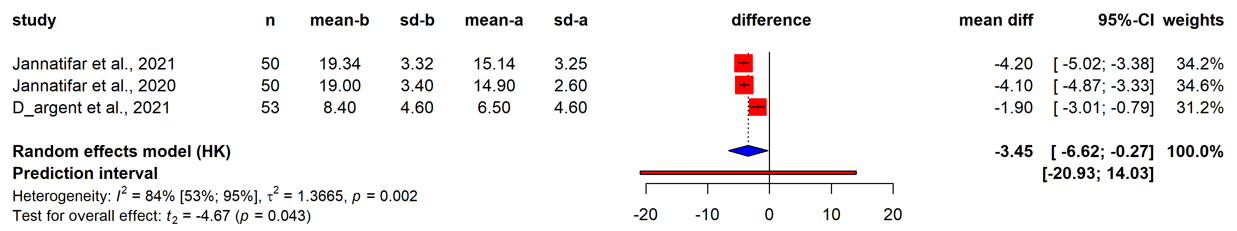
 **Supplementary Figure 23:** Comparison of patients’ mean sperm DNA fragmentation values with a 95% confidence interval measured via TUNEL assay before and 3 months after a single type of antioxidant supplementation with an input correlation of 0.6


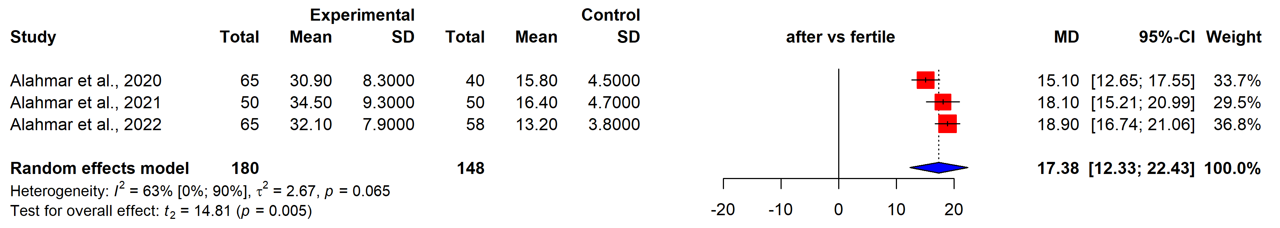
 **Supplementary Figure 24:** Comparison of patients’ and fertile controls’ mean sperm DNA fragmentation values with a 95% confidence interval measured via SCD 3 months following patients’ single type of antioxidant supplementation

**
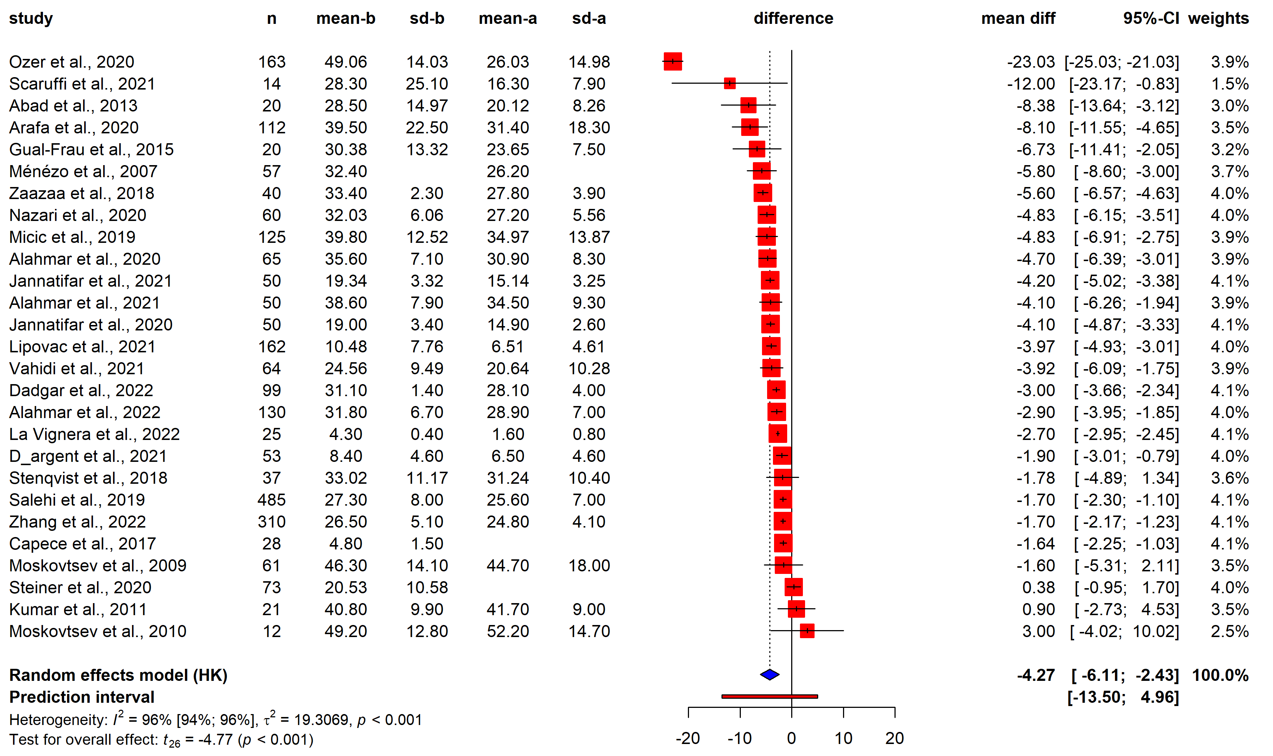
**

**
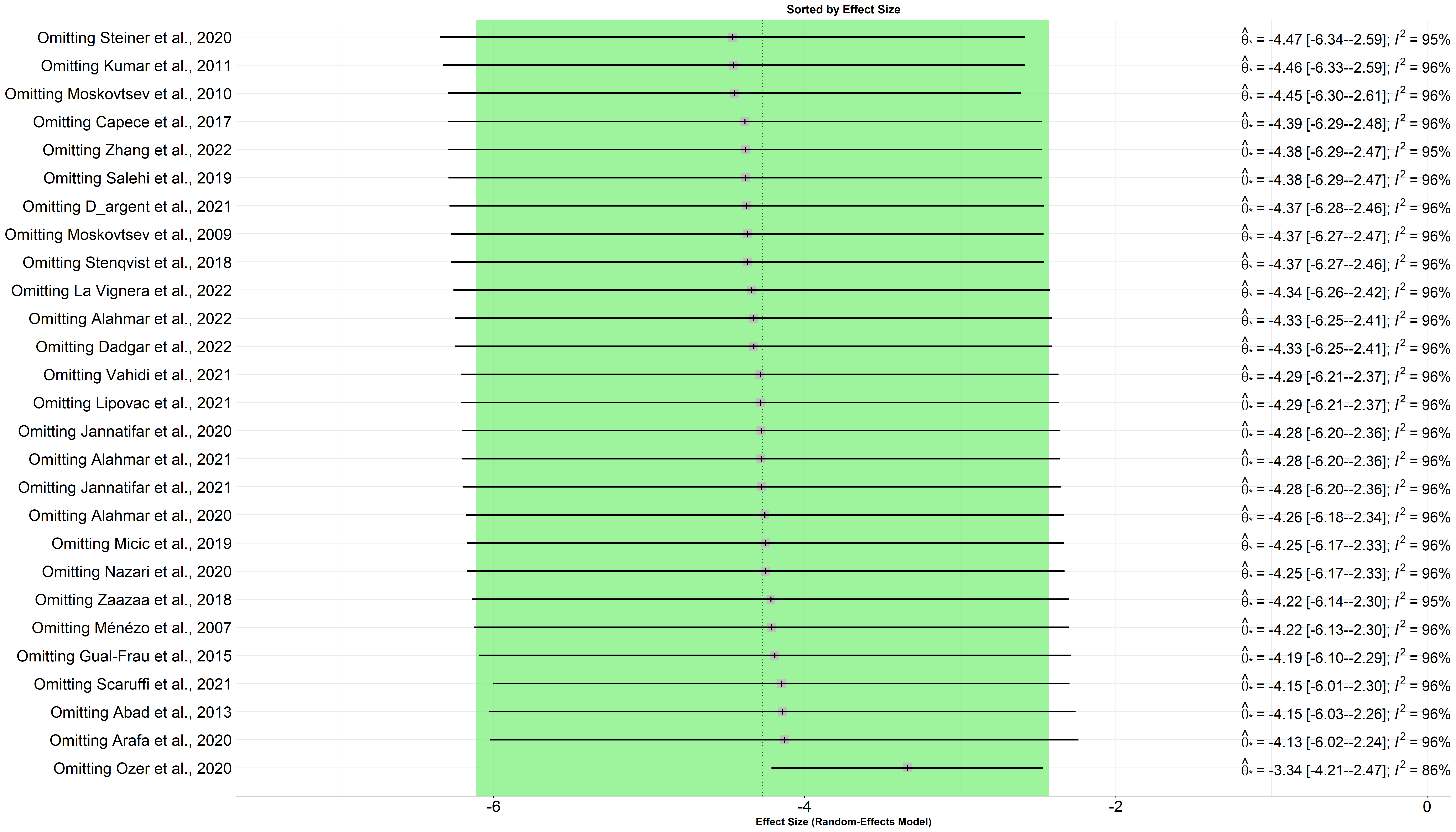
**

**
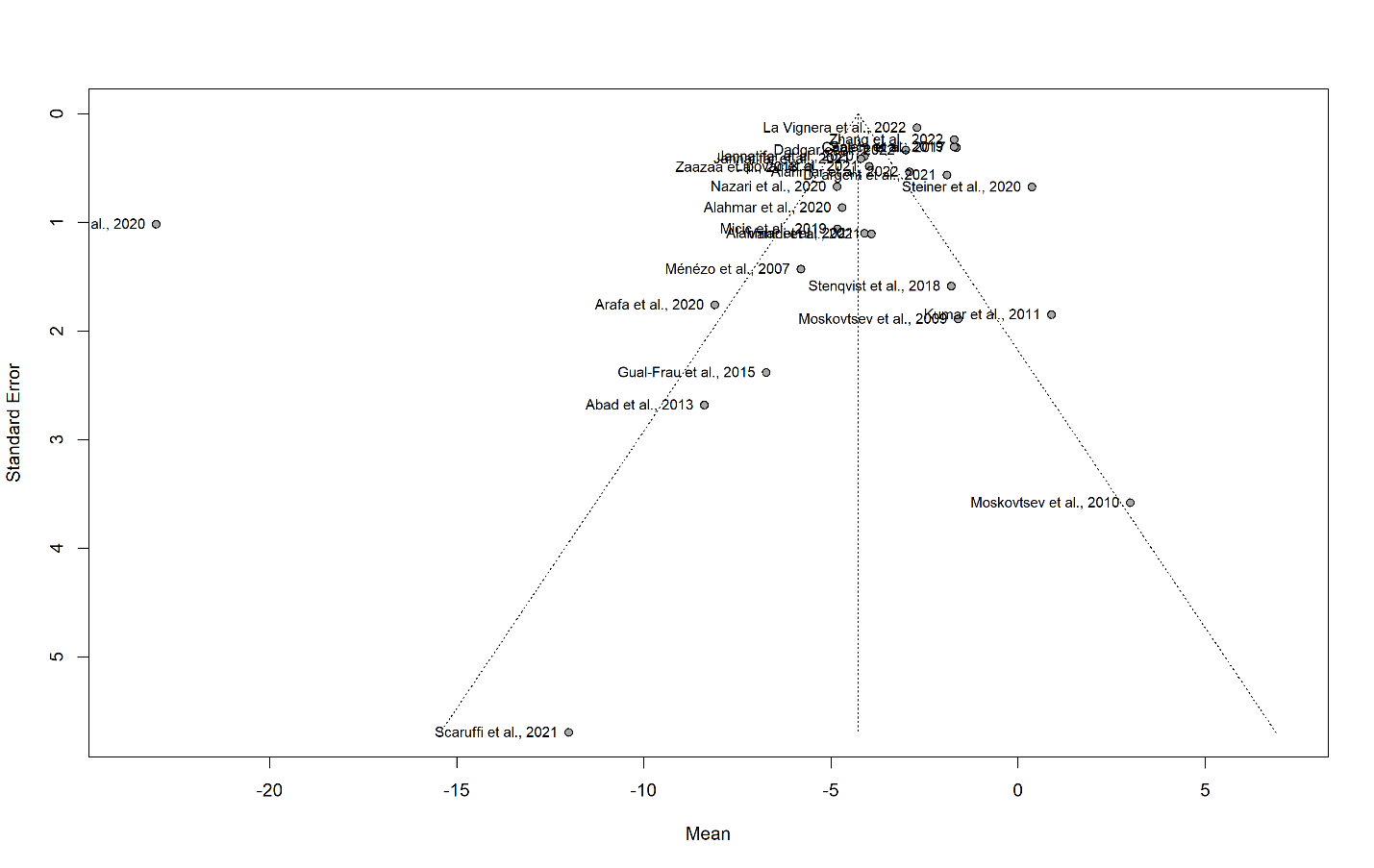
Supplementary Figure 25:** Comparison of patients’ mean sperm DNA fragmentation values with a 95% confidence interval before and 3 months after antioxidant supplementation with an input correlation of 0.6 **B:** Corresponding leave-one-out analysis **C:** Corresponding funnel plot

**
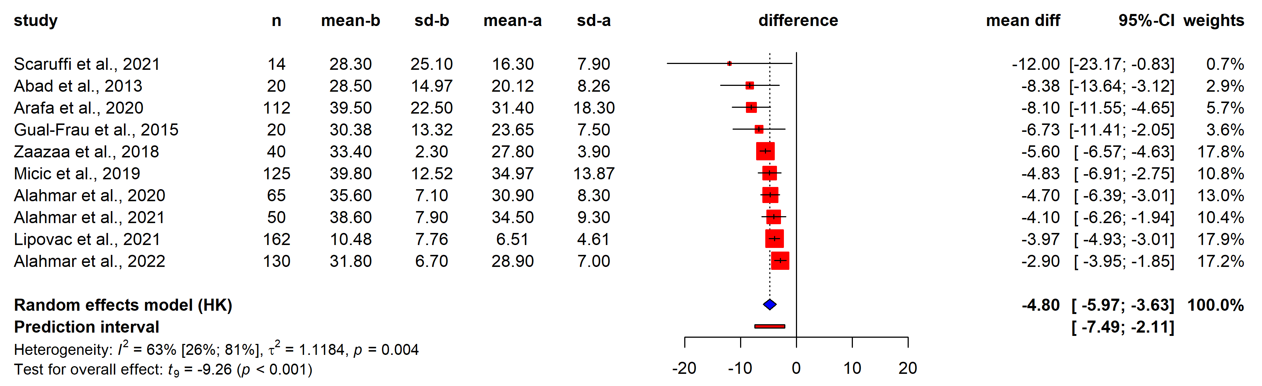
 Supplementary Figure 26:** Comparison of patients’ mean sperm DNA fragmentation values with a 95% confidence interval measured via SCD before and 3 months after antioxidant supplementation with an input correlation of 0.6


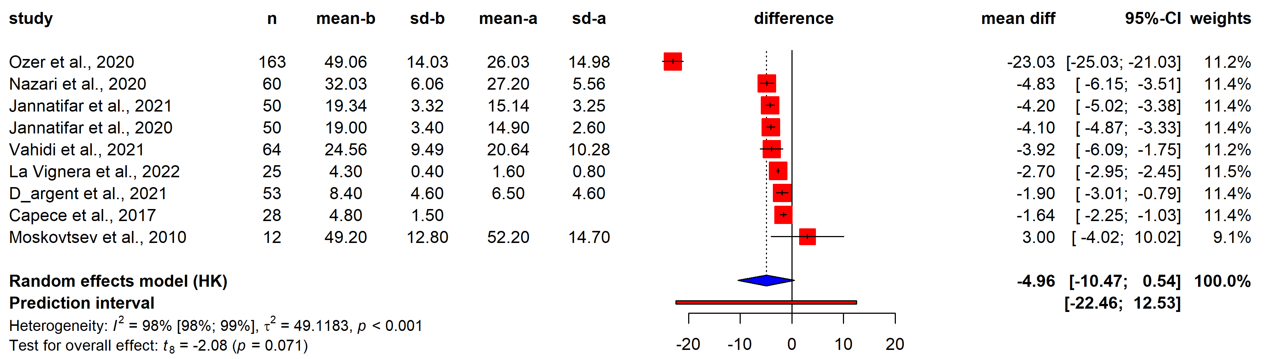
 **Supplementary Figure 27:** Comparison of patients’ mean sperm DNA fragmentation values with a 95% confidence interval measured via TUNEL assay before and 3 months after antioxidant supplementation with an input correlation of 0.6


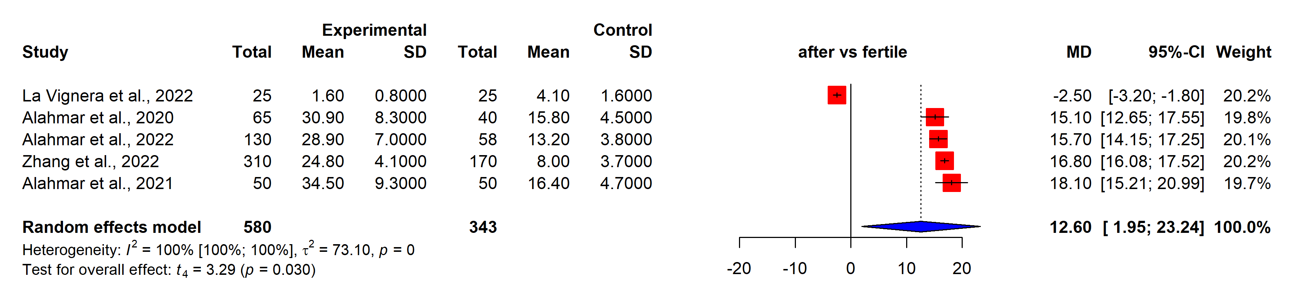
 **Supplementary Figure 28:** Comparison of patients’ and fertile controls’ mean sperm DNA fragmentation values with a 95% confidence interval 3 months following patients’ antioxidant supplementation

**
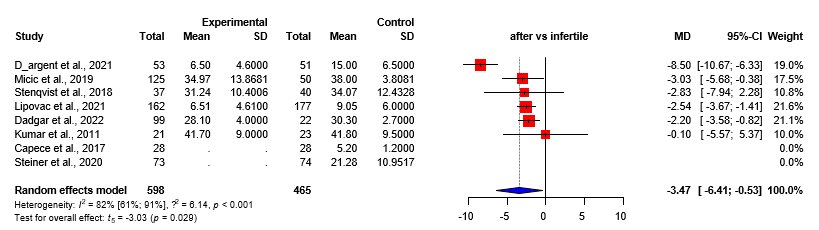
 Supplementary Figure 29:** Comparison of patients’ and infertile controls’ mean sperm DNA fragmentation values with a 95% confidence interval 3 months following patients’ antioxidant supplementation


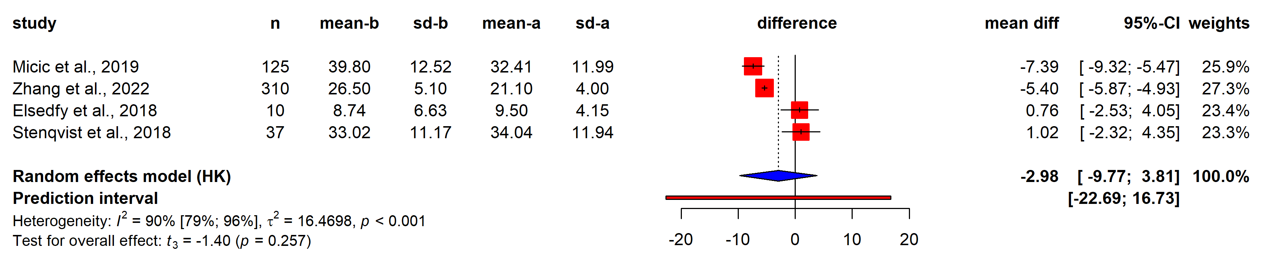
 **Supplementary Figure 30:** Comparison of patients’ mean sperm DNA fragmentation values with a 95% confidence interval before and 6 months after combined antioxidant supplementation with an input correlation of 0.6

**
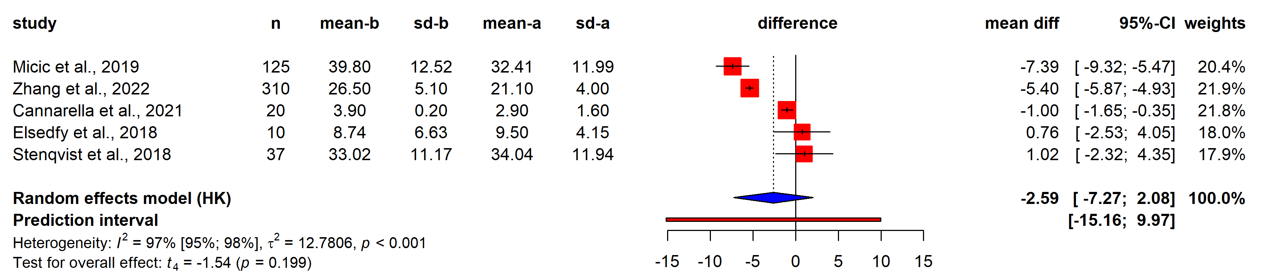
 Supplementary Figure 31:** Comparison of patients’ mean sperm DNA fragmentation values with a 95% confidence interval before and 6 months after antioxidant supplementation with an input correlation of 0.6


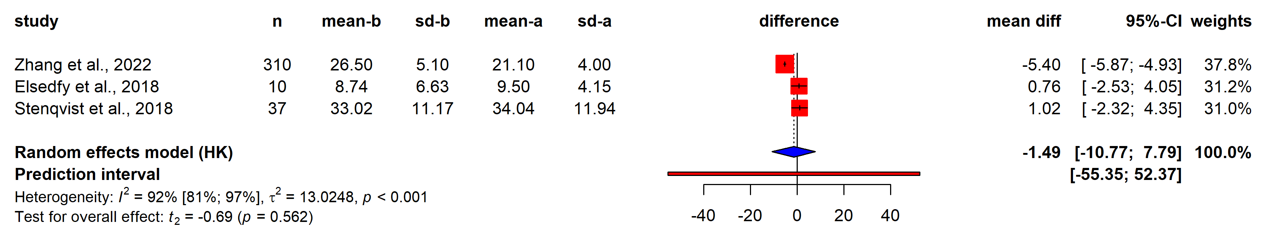
 **Supplementary Figure 32:** Comparison of patients’ mean sperm DNA fragmentation values with a 95% confidence interval measured via SCSA before and 6 months after antioxidant supplementation with an input correlation of 0.6


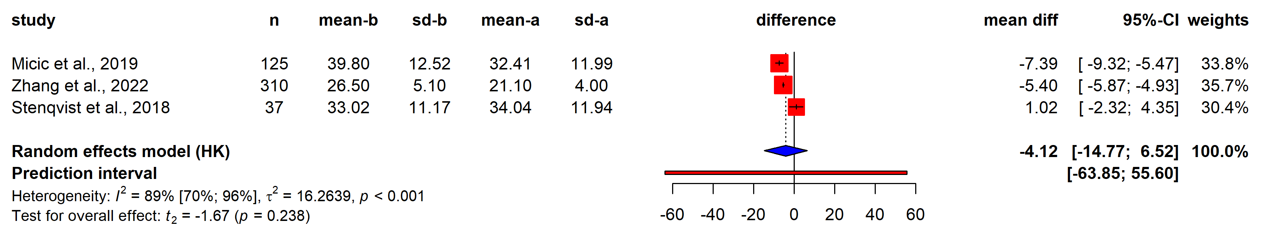
 **Supplementary Figure 33:** Comparison of mean sperm DNA fragmentation values with a 95% confidence interval of patients with no comorbidities before and 6 months after antioxidant supplementation with an input correlation of 0.6


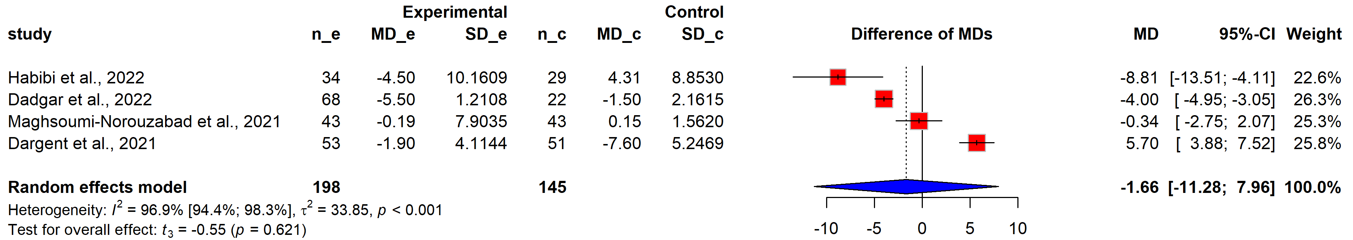


**Supplementary Figure 34:** Mean difference comparison of sperm DNA fragmentation values with a 95% confidence interval before and after single antioxidant supplementation of any duration with an input correlation of 0.6 including randomized controlled trials only


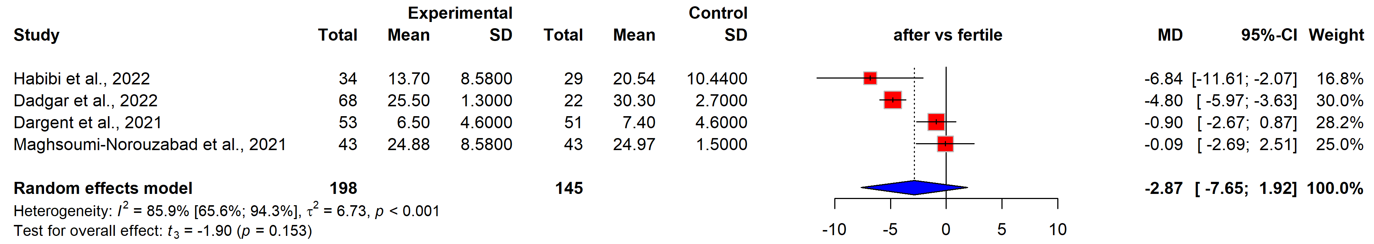


**Supplementary Figure 35:** Comparison of patients’ post-treatment and infertile controls’ mean sperm DNA fragmentation values with a 95% confidence interval before and after single antioxidant supplementation of any duration with an input correlation of 0.6 including randomized controlled trials only

**
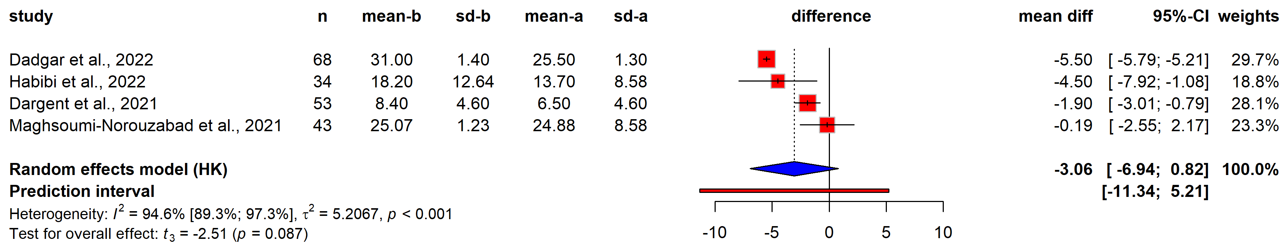
 Supplementary Figure 36:** Comparison of mean sperm DNA fragmentation values with a 95% confidence interval before and after single antioxidant supplementation of any duration with an input correlation of 0.6 including randomized controlled trials only


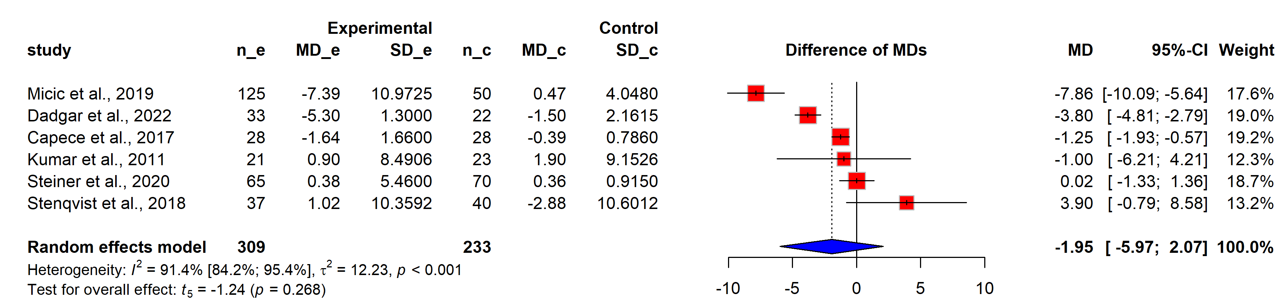
**Supplementary Figure 37:** Mean difference comparison of sperm DNA fragmentation values with a 95% confidence interval before and 3 months after combined antioxidant supplementation with an input correlation of 0.6 including randomized controlled trials only


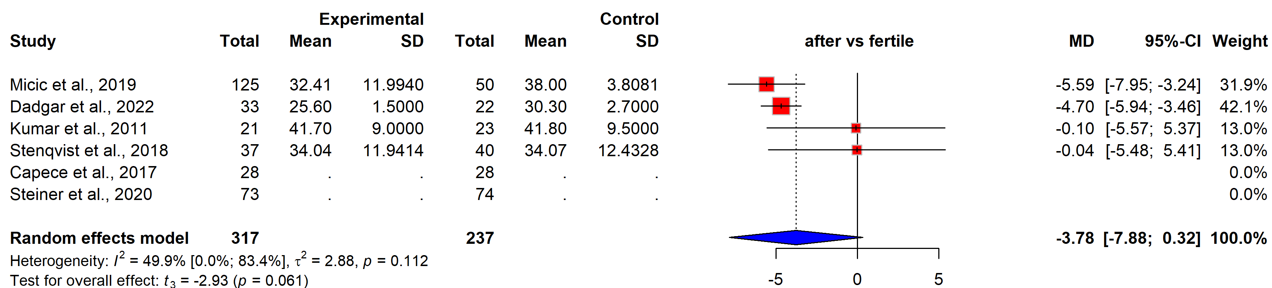


**Supplementary Figure 38:** Comparison of patients’ post-treatment and infertile controls’ mean sperm DNA fragmentation values with a 95% confidence interval before and 3 months after combined antioxidant supplementation with an input correlation of 0.6 including randomized controlled trials only


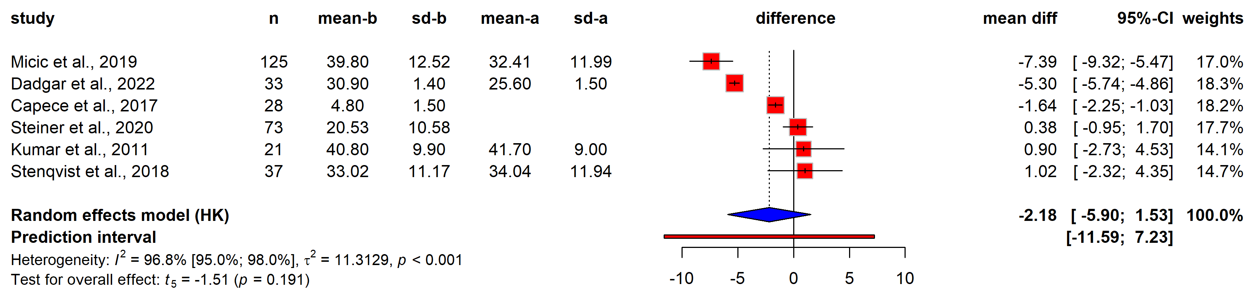


**Supplementary Figure 39:** Comparison of patients’ mean sperm DNA fragmentation values with a 95% confidence interval before and 3 months after combined antioxidant supplementation with an input correlation of 0.6 including randomized controlled trials only


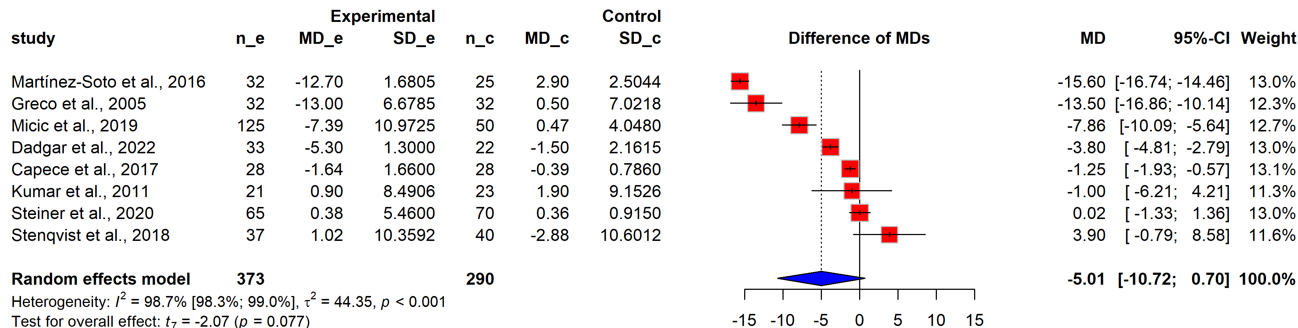


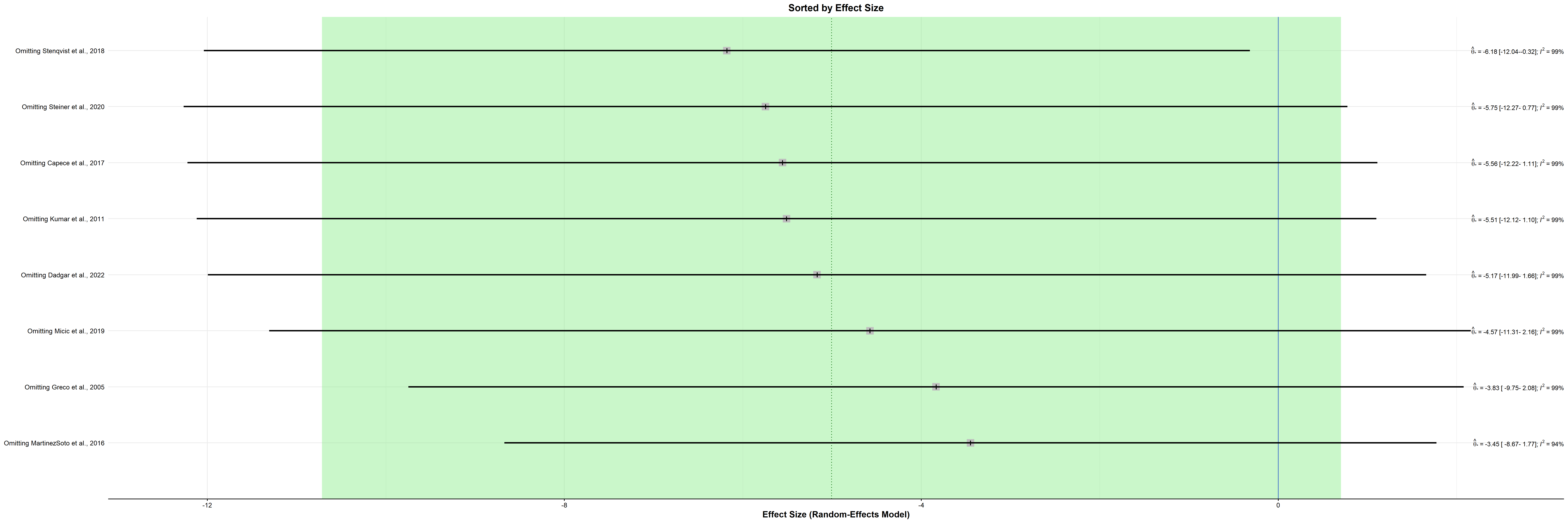


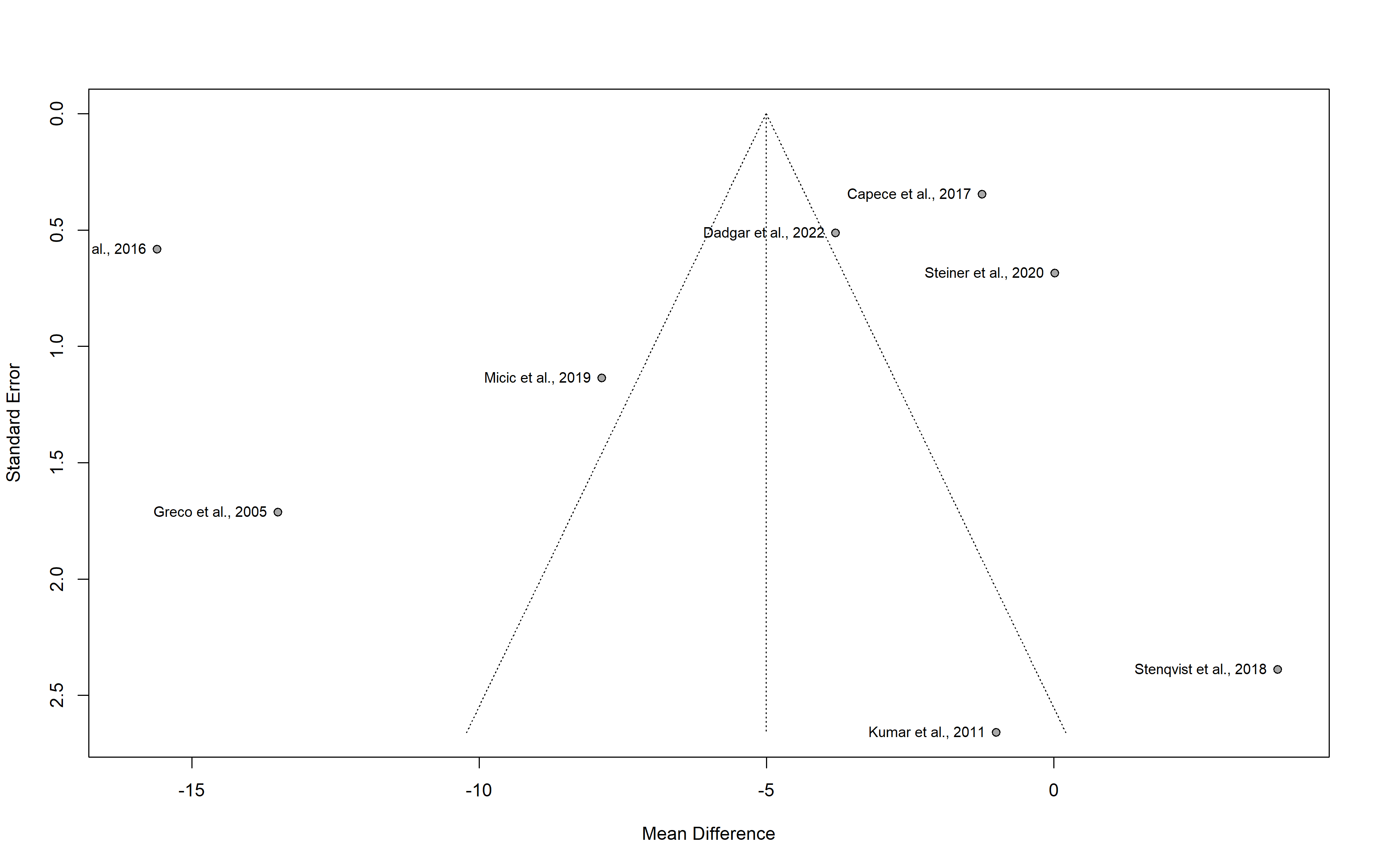


**Supplementary Figure 40/A:** Mean difference comparison of sperm DNA fragmentation values with a 95% confidence interval before and after combined antioxidant supplementation of any duration with an input correlation of 0.6 including randomized controlled trials only **B:** Corresponding leave-one-out analysis **C:** Corresponding funnel plot


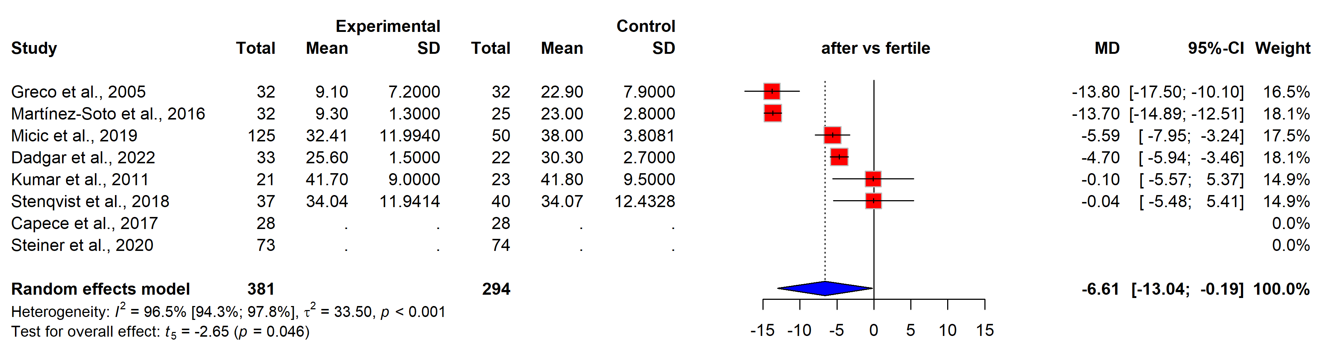


**Supplementary Figure 41:** Comparison of patients’ post-treatment and infertile controls’ mean sperm DNA fragmentation values with a 95% confidence interval before and after combined antioxidant supplementation of any duration with an input correlation of 0.6 including randomized controlled trials only


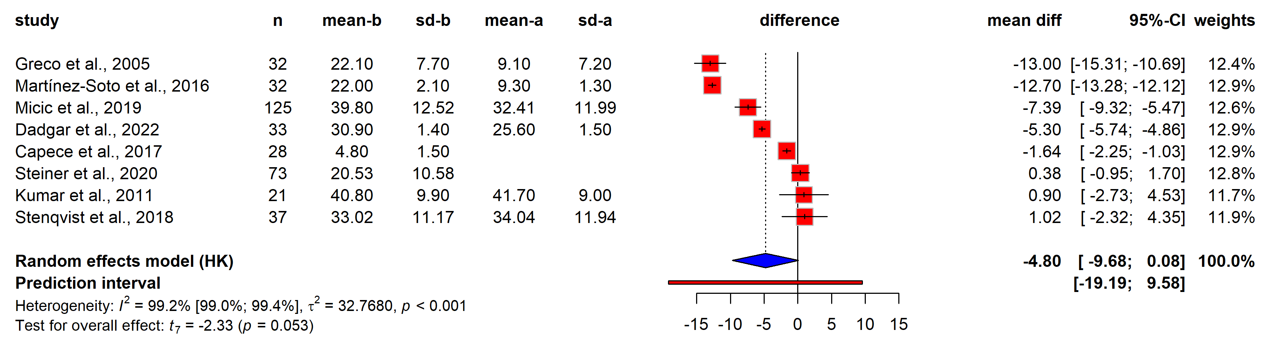


**Supplementary Figure 42:** Comparison of mean sperm DNA fragmentation values with a 95% confidence interval before and after combined antioxidant supplementation of any duration with an input correlation of 0.6 including randomized controlled trials only


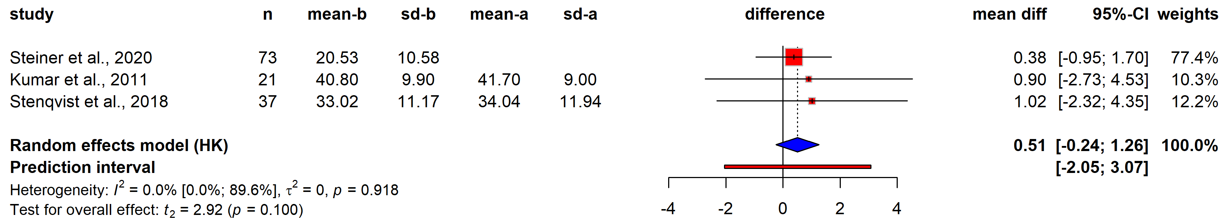


**Supplementary Figure 43:** Comparison of mean sperm DNA fragmentation values with a 95% confidence interval measured via SCSA before and 3 months after combined antioxidant supplementation of any duration with an input correlation of 0.6 including randomized controlled trials only


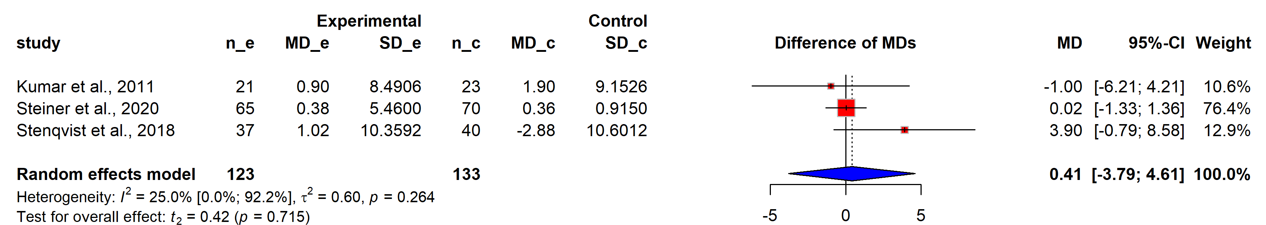


**Supplementary Figure 44:** Mean difference comparison of sperm DNA fragmentation values with a 95% confidence interval measured via SCSA before and 3 months after combined antioxidant supplementation with an input correlation of 0.6 including randomized controlled trials only

**Follicle stimulating hormone (FSH) treatment:**

**
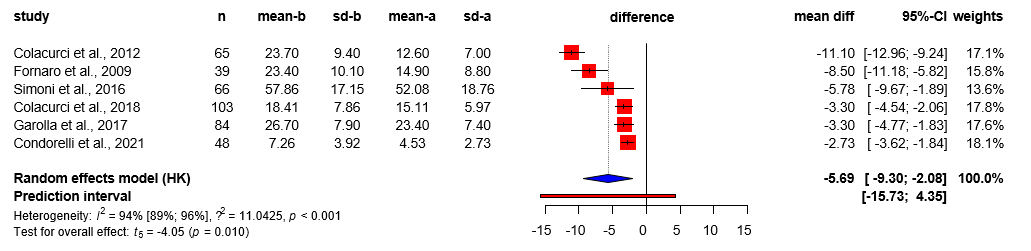
 Supplementary Figure 45:** Comparison of patients’ mean sperm DNA fragmentation values with a 95% confidence interval measured via TUNEL assay before and 3 months after FSH treatment with an input correlation of 0.6

**
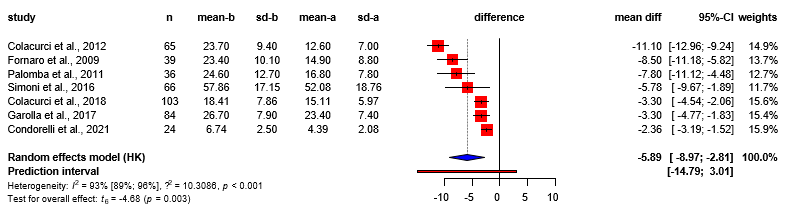
 Supplementary Figure 46: C**omparison of patients’ mean sperm DNA fragmentation values with a 95% confidence interval before and 3 months after 150 IU dosages of FSH treatment every other day with an input correlation of 0.6

**
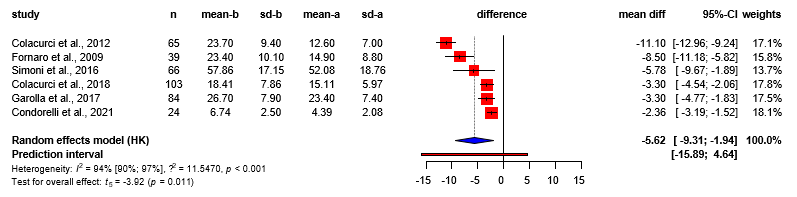
 Supplementary Figure 47:** Comparison of patients’ mean sperm DNA fragmentation values with a 95% confidence interval measured via TUNEL assay before and 3 months after 150 IU dosages of FSH treatment every other day with an input correlation of 0.6

**Lifestyle interventions:**

**
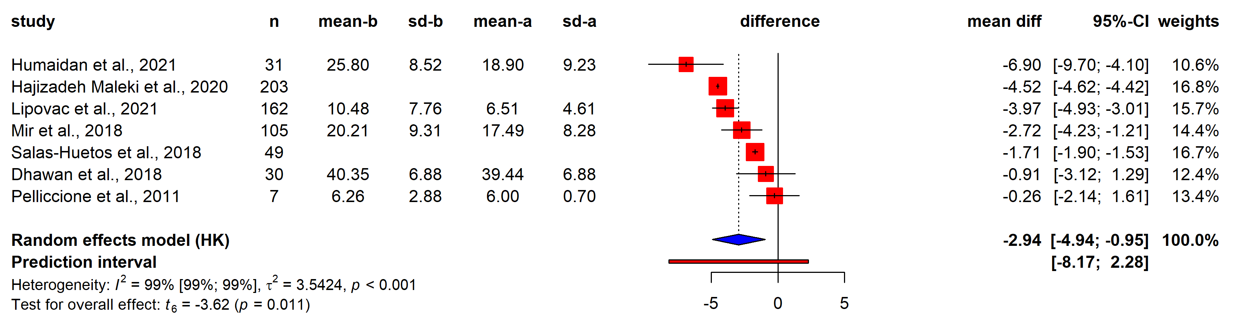
 Supplementary Figure 48:** Comparison of patients’ mean sperm DNA fragmentation values with a 95% confidence interval before and 3 months after lifestyle interventions with an input correlation of 0.6


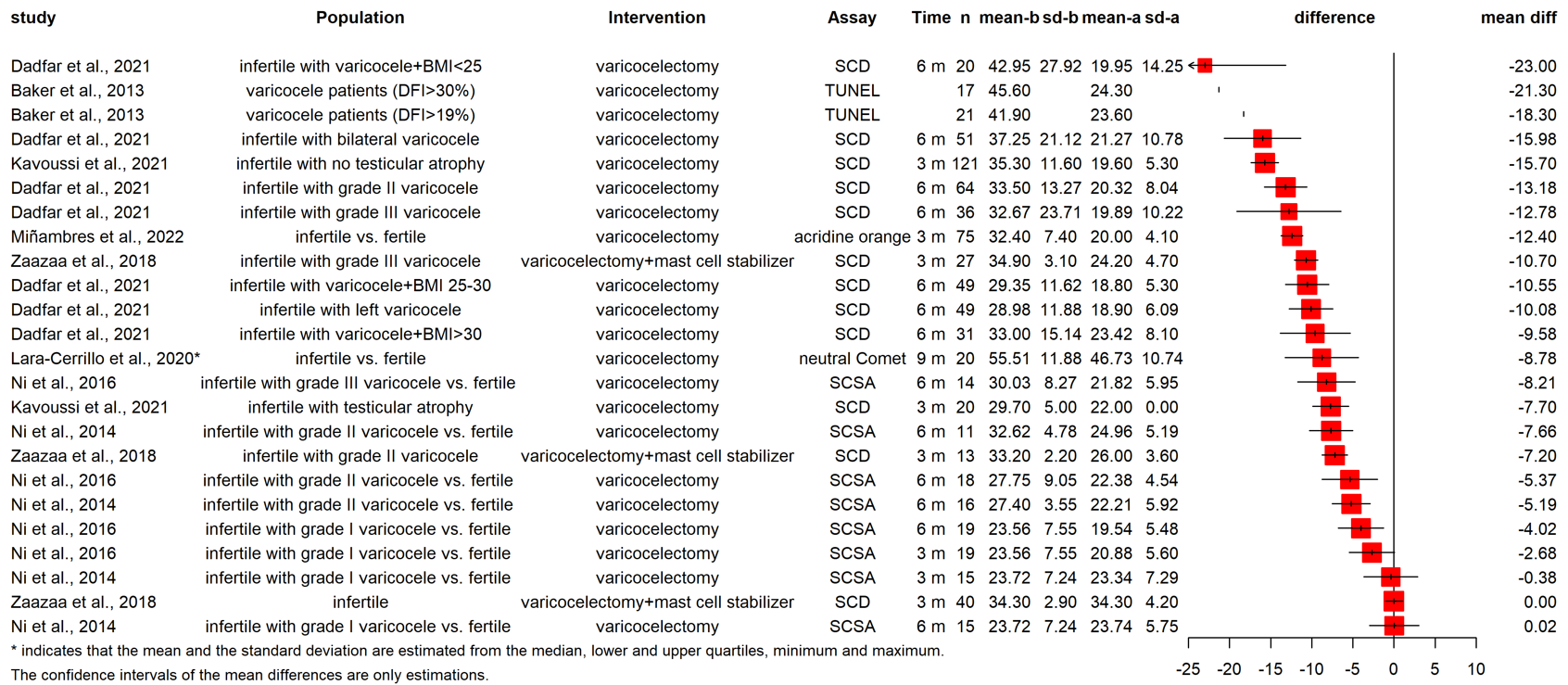
 **Supplementary Figure 49:** Summary figure of varicocelectomy not categorized otherwise with an input correlation of 0.6 and 95% CI (n: number of men involved, m: months, CI: confidence interval, DFI: DNA fragmentation index, BMI: body mass index)

**
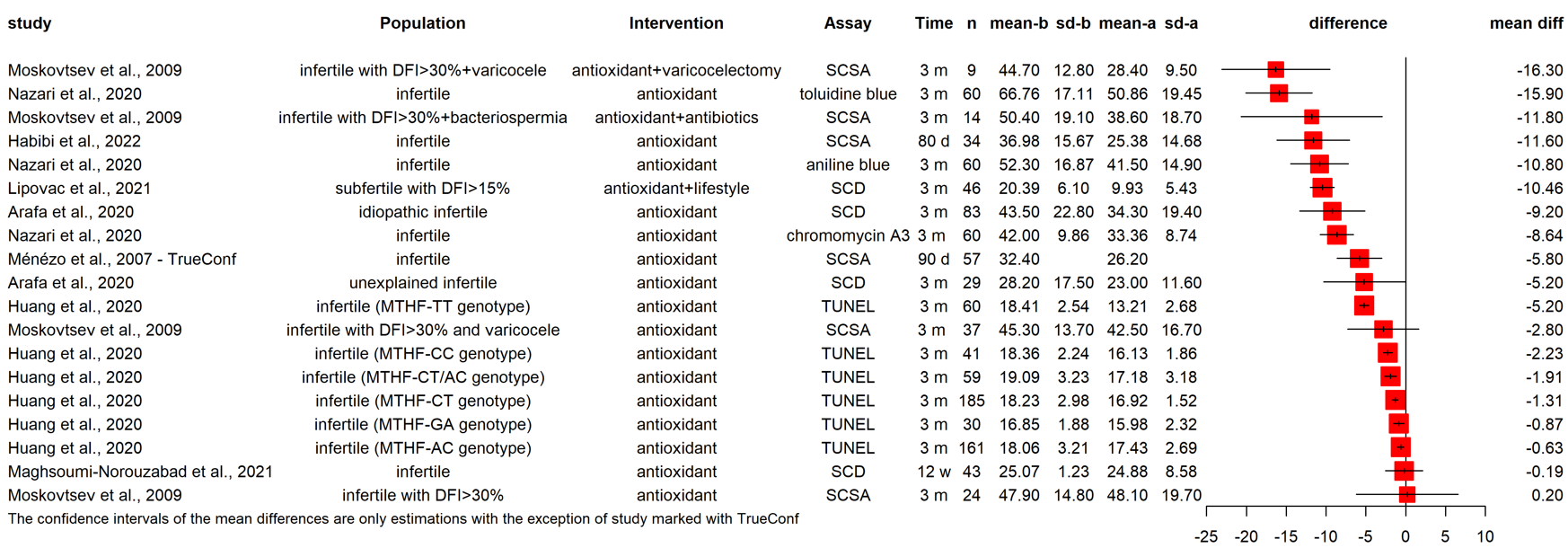
 Supplementary Figure 50:** Summary figure of antioxidant supplementations not categorized otherwise with an input correlation of 0.6 and 95% CI (n: number of men involved, CI: confidence interval, m: months, w: weeks, d: days, DFI: DNA fragmentation index)


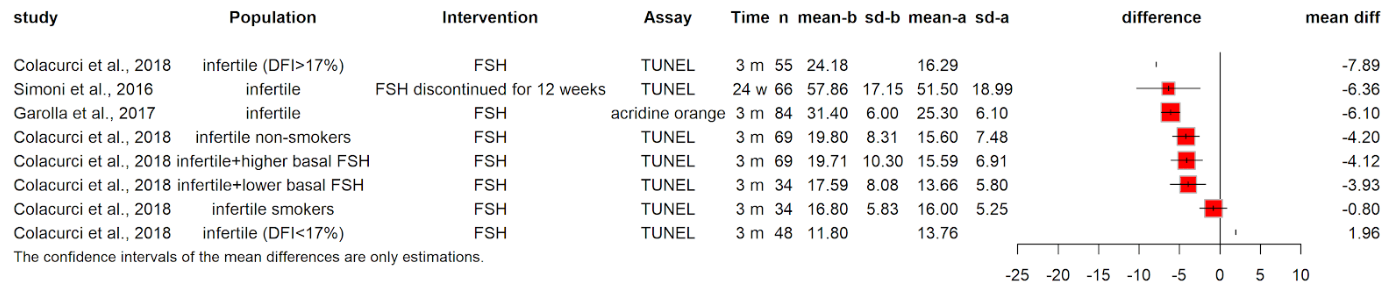
 **Supplementary Figure 51:** Summary figure of FSH treatments not categorized otherwise with an input correlation of 0.6 and 95% CI (n: number of men involved, CI: confidence interval, m: months, w: weeks, d: days, hCG: human chorionic gonadotropin)

**Additional diagrams:** The results below show that the final results do not depend on the correlation. However, the individual confidence intervals can be different. I highlight that, in this case, the forest plots show unpublished results. The studies do not report the confidence of the differences.

**Varicocelectomy:**


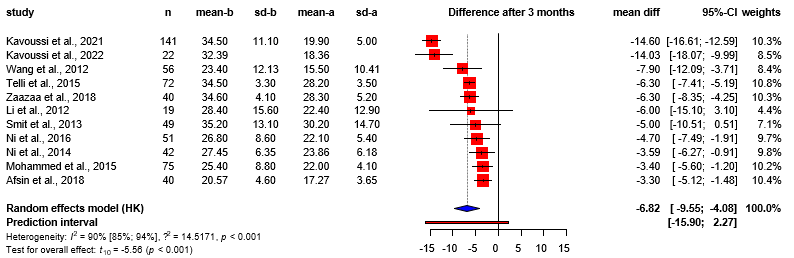
 **Supplementary Figure 52:** Comparison of patients’ mean sperm DNA fragmentation values with a 95% confidence interval before and 3 months after varicocelectomy with an input correlation of 0


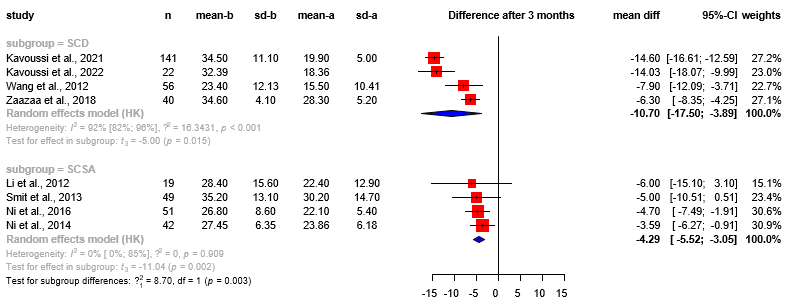
 **Supplementary Figure 53:** Comparison of patients’ mean sperm DNA fragmentation values with a 95% confidence interval based on assay types before and 3 months after varicocelectomy with an input correlation of 0


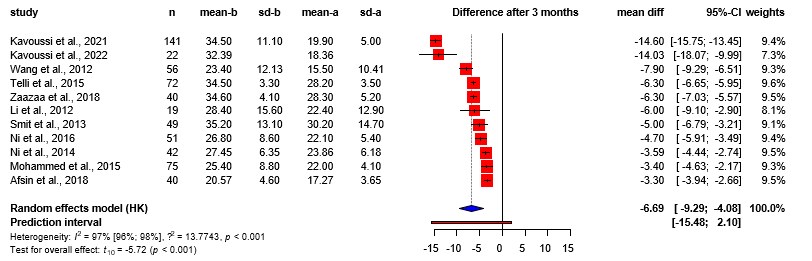
 **Supplementary Figure 54:** Comparison of patients’ mean sperm DNA fragmentation values with a 95% confidence interval before and 3 months after varicocelectomy with an input correlation of 0.9


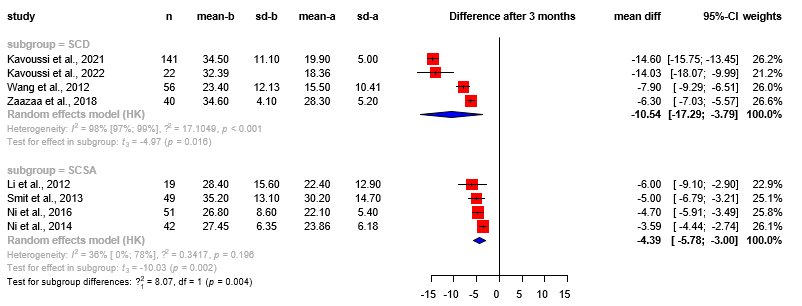
 **Supplementary Figure 55:** Comparison of patients’ mean sperm DNA fragmentation values with a 95% confidence interval based on assay types before and 3 months after varicocelectomy with an input correlation of 0.9


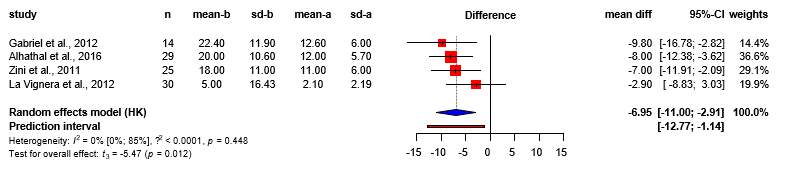
 **Supplementary Figure 56:** Comparison of patients’ mean sperm DNA fragmentation values with a 95% confidence interval before and 4 months after varicocelectomy with an input correlation of 0

**
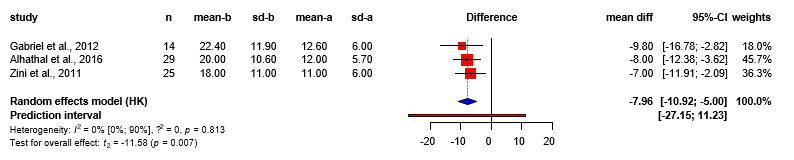
 Supplementary Figure 57:** Comparison of patients’ mean sperm DNA fragmentation values with a 95% confidence interval via SCSA before and 4 months after varicocelectomy with an input correlation of 0

**
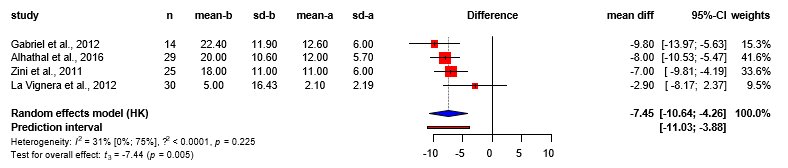
 Supplementary Figure 58:** Comparison of patients’ mean sperm DNA fragmentation values with a 95% confidence interval before and 4 months after varicocelectomy with an input correlation of 0.8


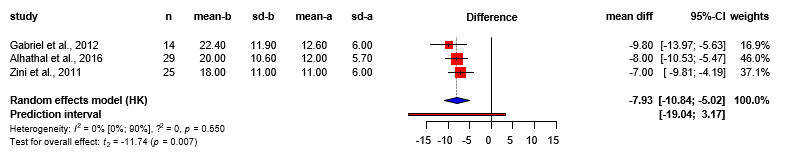
 **Supplementary Figure 59:** Comparison of patients’ mean sperm DNA fragmentation values with a 95% confidence interval via SCSA before and 4 months after varicocelectomy with an input correlation of 0.8

**
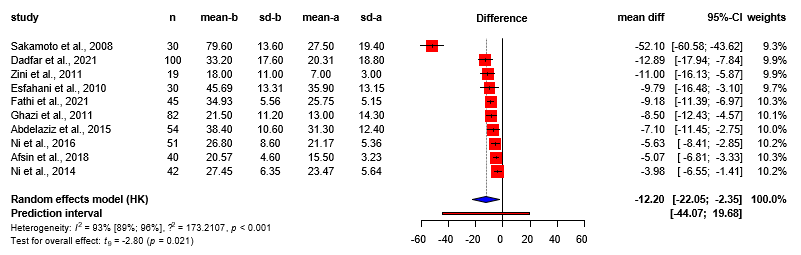
 Supplementary Figure 60:** Comparison of patients’ mean sperm DNA fragmentation values with a 95% confidence interval before and 6 months after varicocelectomy with an input correlation of 0


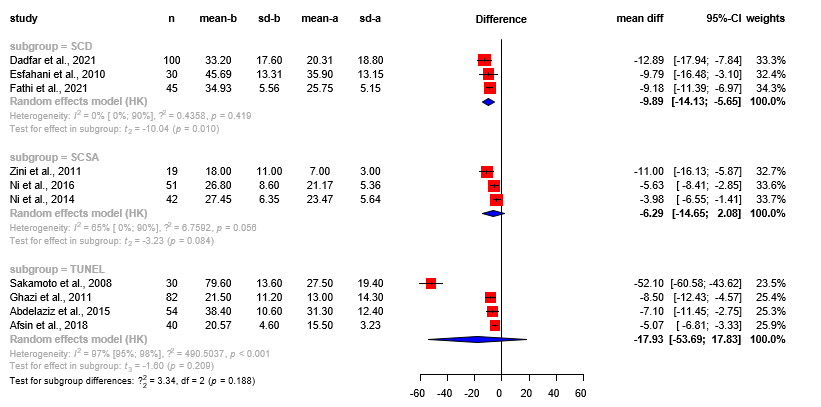
 **Supplementary Figure 61:** Comparison of patients’ mean sperm DNA fragmentation values with a 95% confidence interval based on assay types before and 6 months after varicocelectomy with an input correlation of 0

**
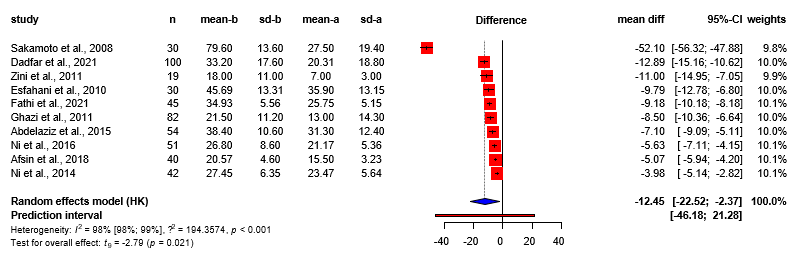
 Supplementary Figure 62:** Comparison of patients’ mean sperm DNA fragmentation values with a 95% confidence interval before and 6 months after varicocelectomy with an input correlation of 0.8

**
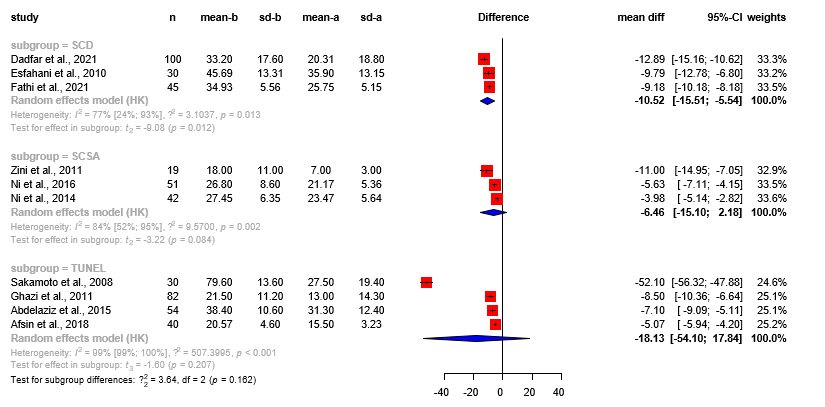
 Supplementary Figure 63:** Comparison of patients’ mean sperm DNA fragmentation values with a 95% confidence interval based on assay types before and 6 months after varicocelectomy with an input correlation of 0.8

**
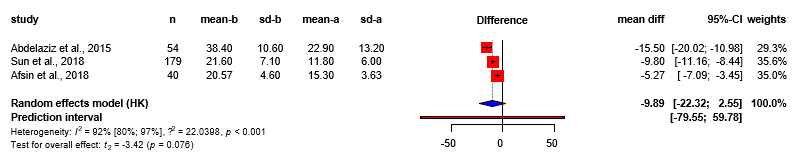
 Supplementary Figure 64:** Comparison of patients’ mean sperm DNA fragmentation values with a 95% confidence interval before and 12 months after varicocelectomy with an input correlation of 0

**
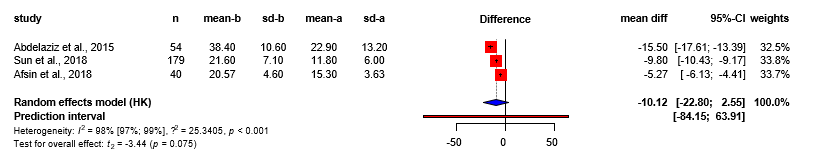
 Supplementary Figure 65:** Comparison of patients’ mean sperm DNA fragmentation values with a 95% confidence interval before and 12 months after varicocelectomy with an input correlation of 0.8


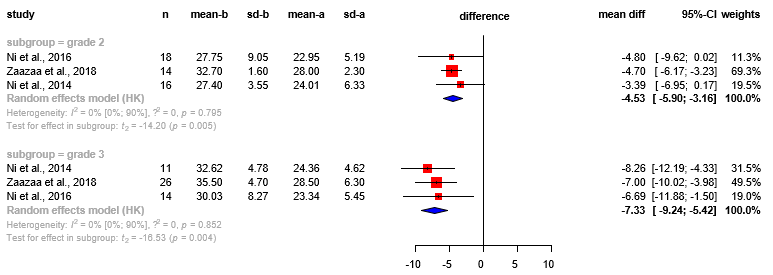
 **Supplementary Figure 66:** Comparison of patients’ mean sperm DNA fragmentation values with a 95% confidence interval based on varicocele grade with an input correlation of 0


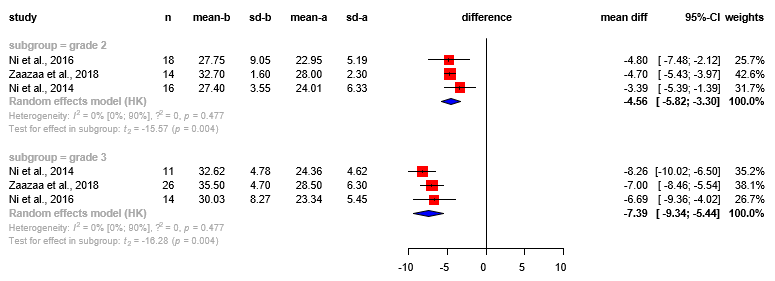
 **Supplementary Figure 67:** Comparison of patients’ mean sperm DNA fragmentation values with a 95% confidence interval based on varicocele grade with an input correlation of 0.8

**Antioxidant supplementation:**


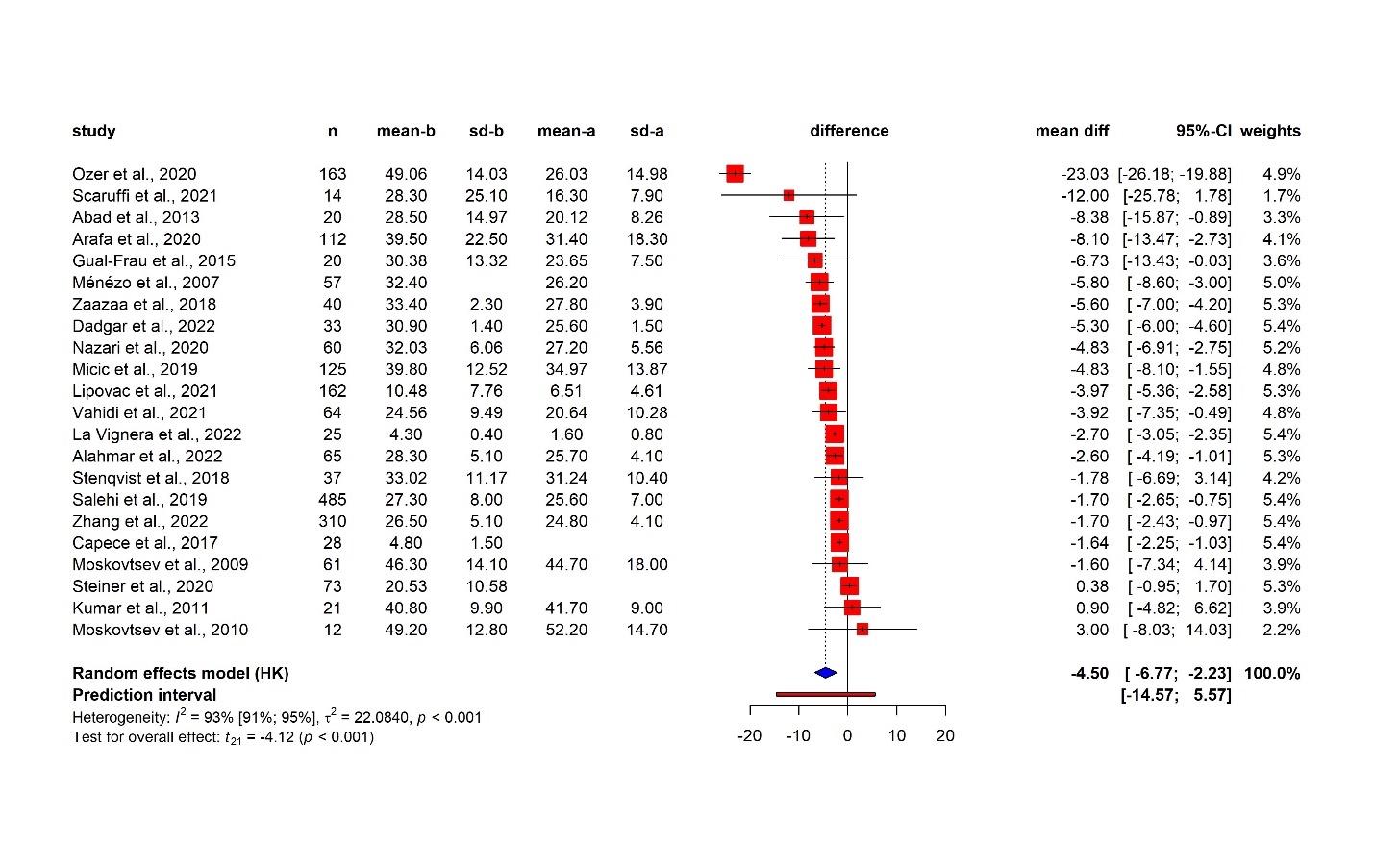
 **Supplementary Figure 68:** Comparison of patients’ mean sperm DNA fragmentation values with a 95% confidence interval before and 3 months after combined antioxidant supplementation with an input correlation of 0


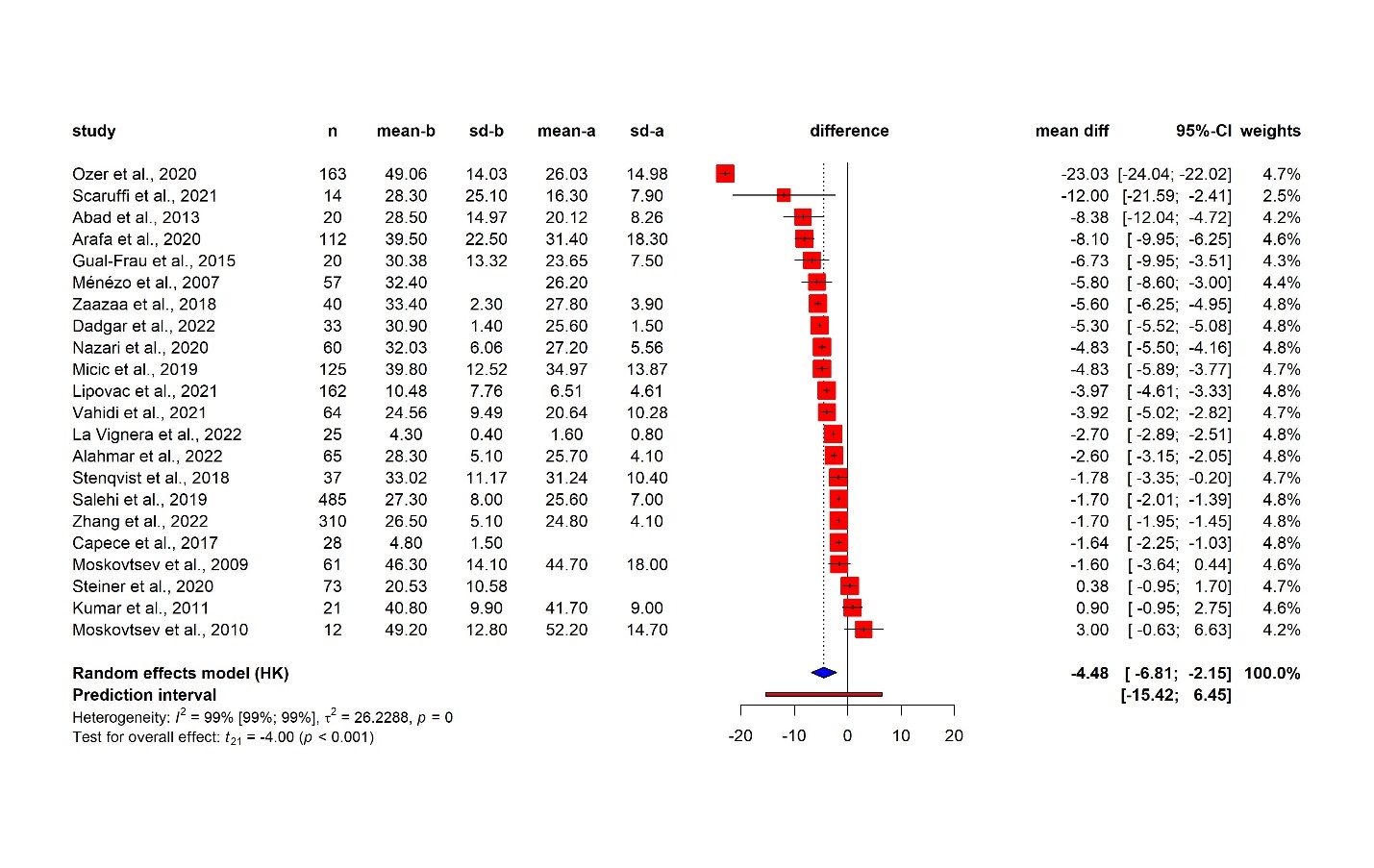
 **Supplementary Figure 69:** Comparison of patients’ mean sperm DNA fragmentation values with a 95% confidence interval before and 3 months after combined antioxidant supplementation with an input correlation of 0.9


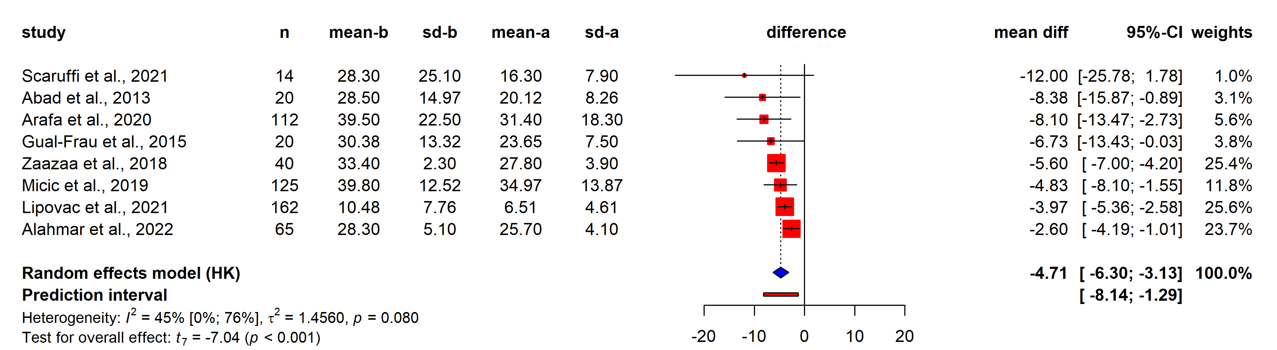
 **Supplementary Figure 70:** Comparison of patients’ mean sperm DNA fragmentation values with a 95% confidence interval measured via SCD before and 3 months after combined antioxidant supplementation with an input correlation of 0


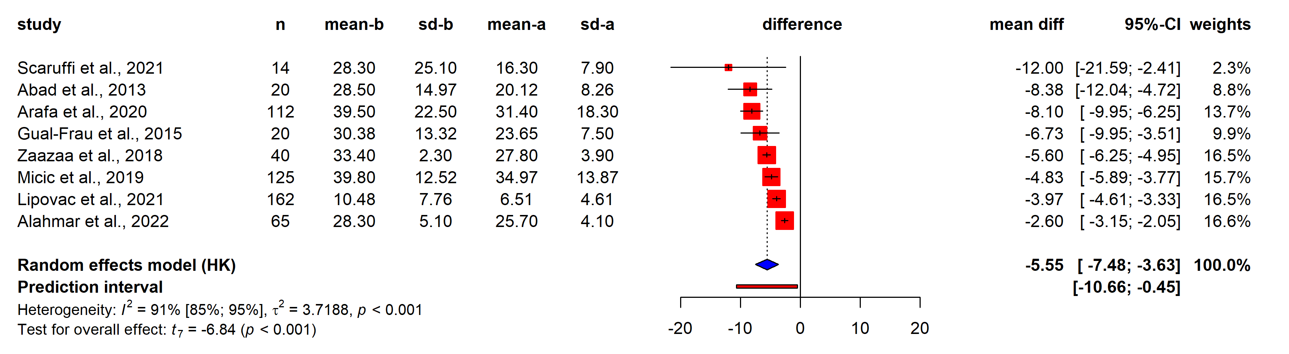
 **Supplementary Figure 71:** Comparison of patients’ mean sperm DNA fragmentation values with a 95% confidence interval measured via SCD before and 3 months after combined antioxidant supplementation with an input correlation of 0.9


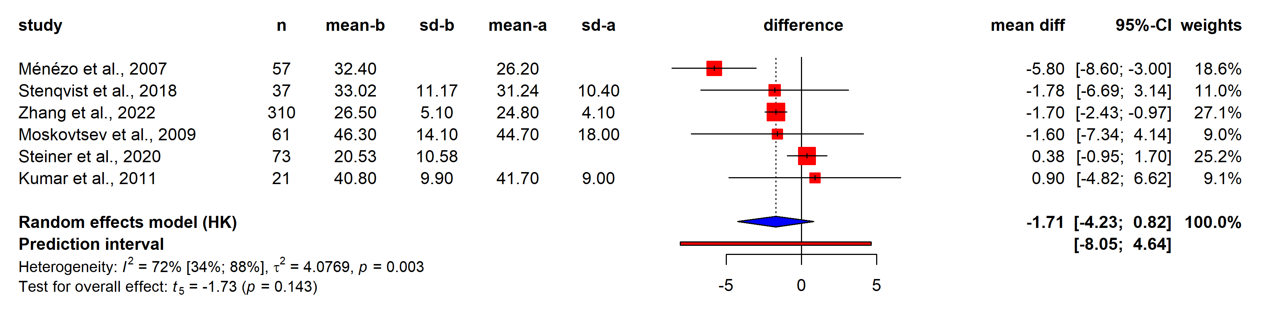
 **Supplementary Figure 72:** Comparison of patients’ mean sperm DNA fragmentation values with a 95% confidence interval measured via SCSA before and 3 months after combined antioxidant supplementation with an input correlation of 0


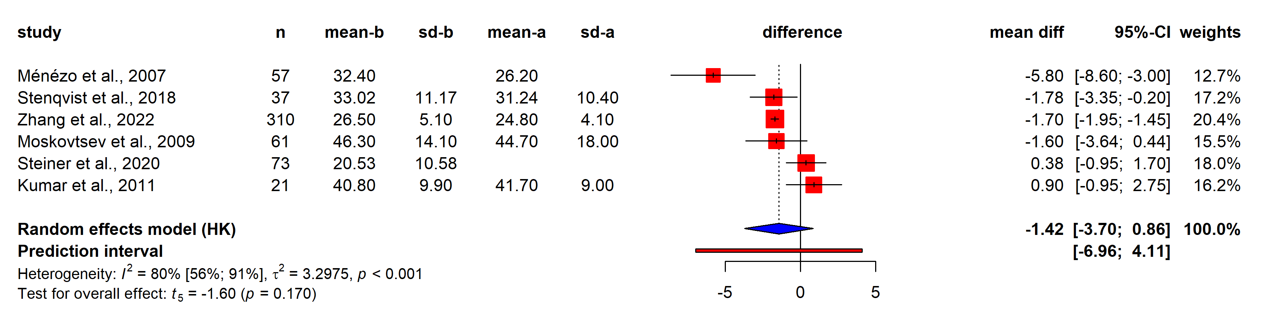
 **Supplementary Figure 73:** Comparison of patients’ mean sperm DNA fragmentation values with a 95% confidence interval measured via SCSA before and 3 months after combined antioxidant supplementation with an input correlation of 0.9


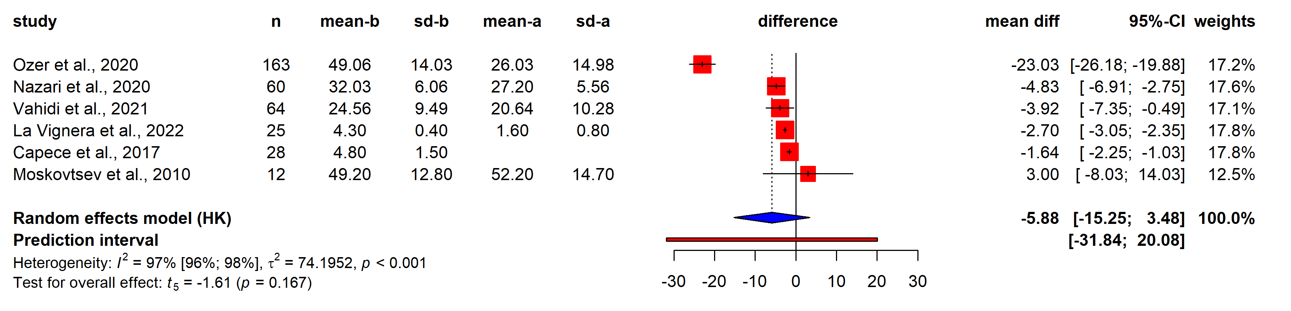
 **Supplementary Figure 74:** Comparison of patients’ mean sperm DNA fragmentation values with a 95% confidence interval measured via TUNEL assay before and 3 months after combined antioxidant supplementation with an input correlation of 0


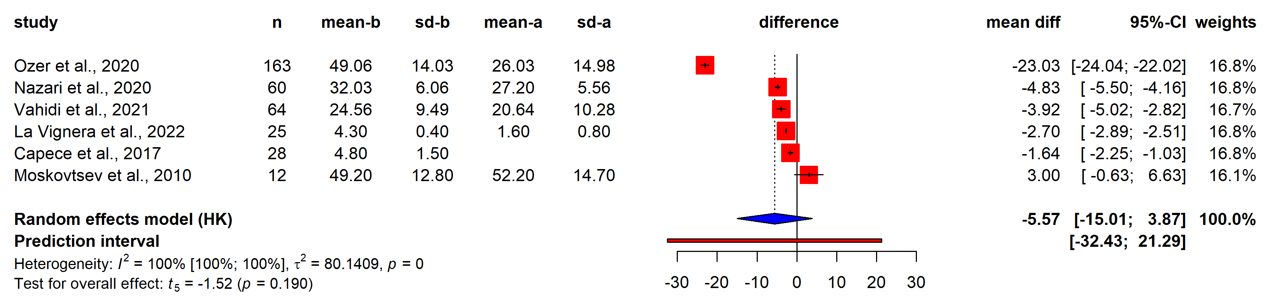
 **Supplementary Figure 75:** Comparison of patients’ mean sperm DNA fragmentation values with a 95% confidence interval measured via TUNEL assay before and 3 months after combined antioxidant supplementation with an input correlation of 0.9


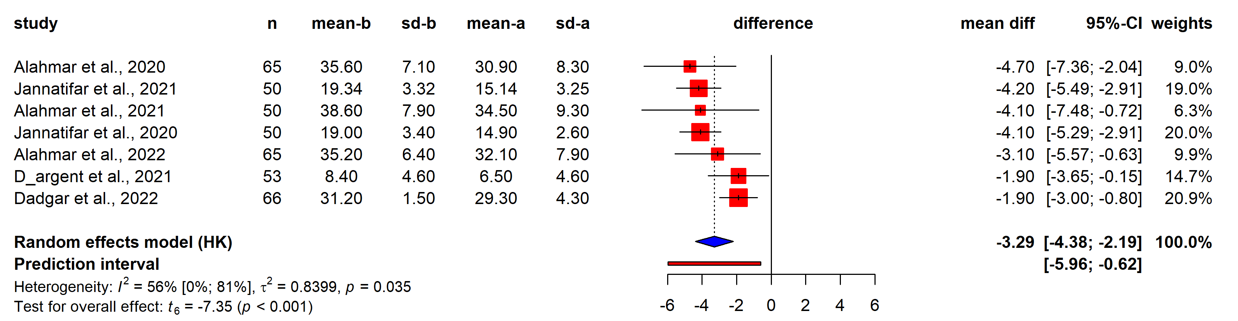
 **Supplementary Figure 76:** Comparison of patients’ mean sperm DNA fragmentation values with a 95% confidence interval before and 3 months after a single type of antioxidant supplementation with an input correlation of 0

**
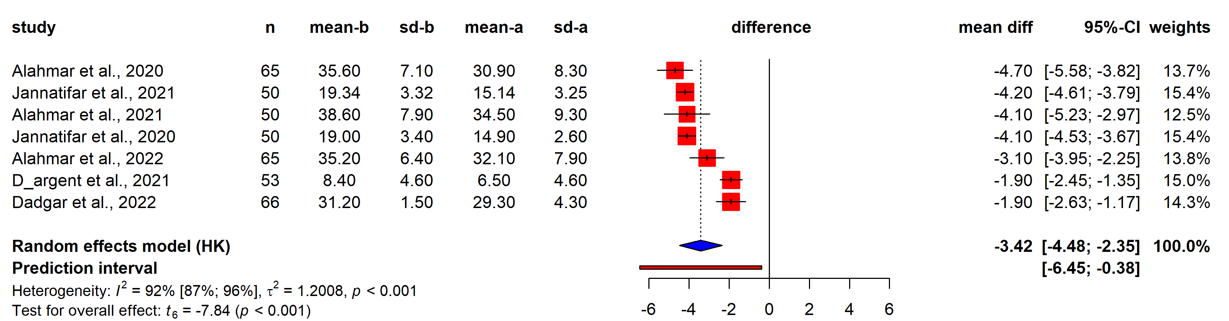
 Supplementary Figure 77:** Comparison of patients’ mean sperm DNA fragmentation values with a 95% confidence interval before and 3 months after a single type of antioxidant supplementation with an input correlation of 0.9


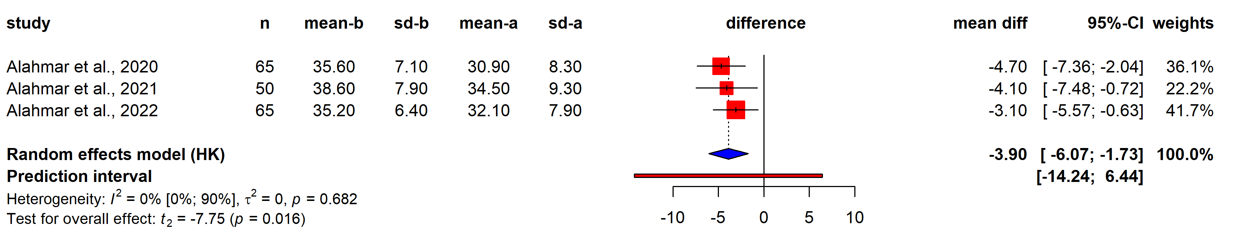
 **Supplementary Figure 78:** Comparison of patients’ mean sperm DNA fragmentation values with a 95% confidence interval measured via SCD before and 3 months after a single type of antioxidant supplementation with an input correlation of 0


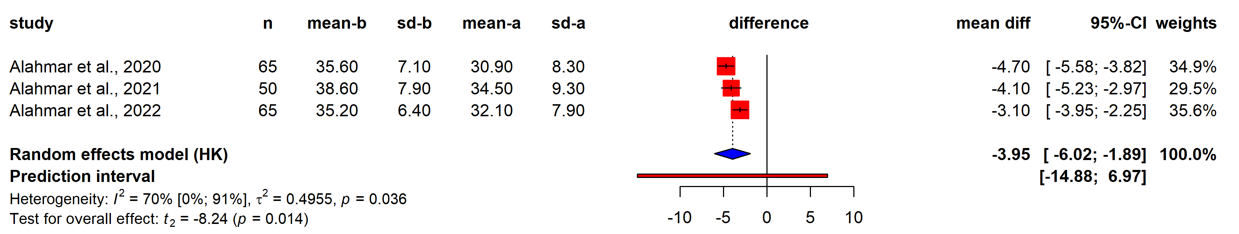
 **Supplementary Figure 79:** Comparison of patients’ mean sperm DNA fragmentation values with a 95% confidence interval measured via SCD before and 3 months after a single type of antioxidant supplementation with an input correlation of 0.9


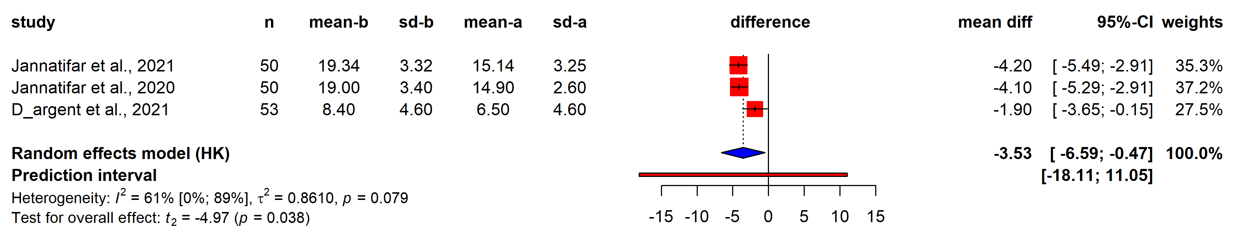
 **Supplementary Figure 80:** Comparison of patients’ mean sperm DNA fragmentation values with a 95% confidence interval measured via TUNEL assay before and 3 months after a single type of antioxidant supplementation with an input correlation of 0


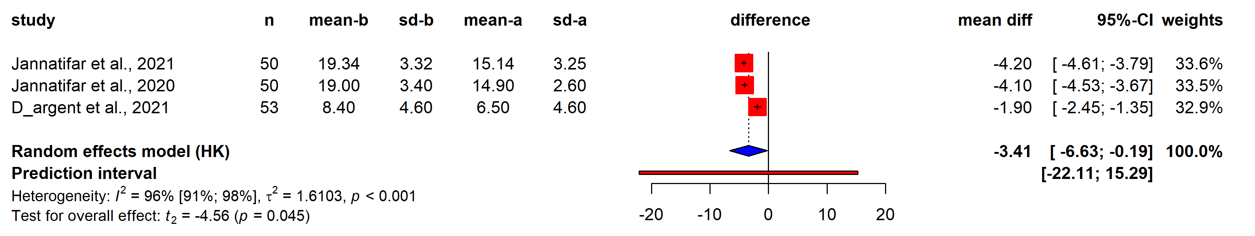
 **Supplementary Figure 81:** Comparison of patients’ mean sperm DNA fragmentation values with a 95% confidence interval measured via TUNEL assay before and 3 months after a single type of antioxidant supplementation with an input correlation of 0.9


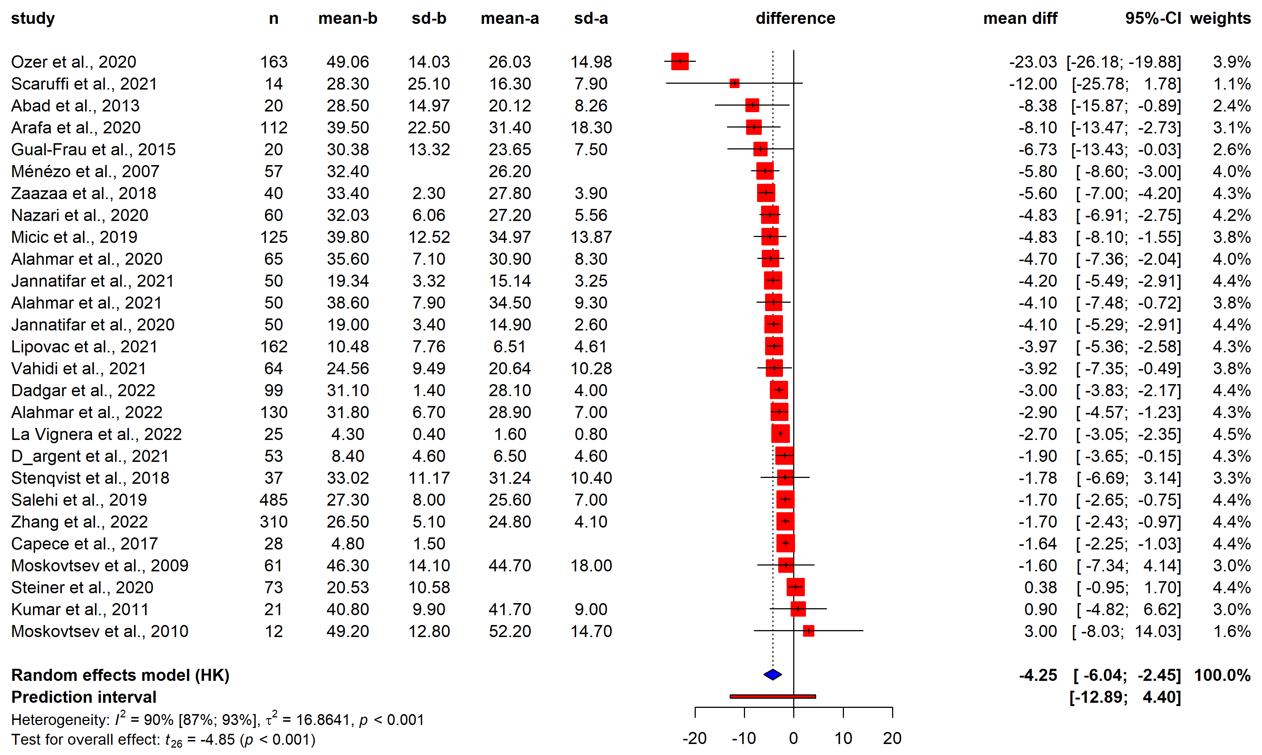
 **Supplementary Figure 82:** Comparison of patients’ mean sperm DNA fragmentation values with a 95% confidence interval before and 3 months after antioxidant supplementation with an input correlation of 0


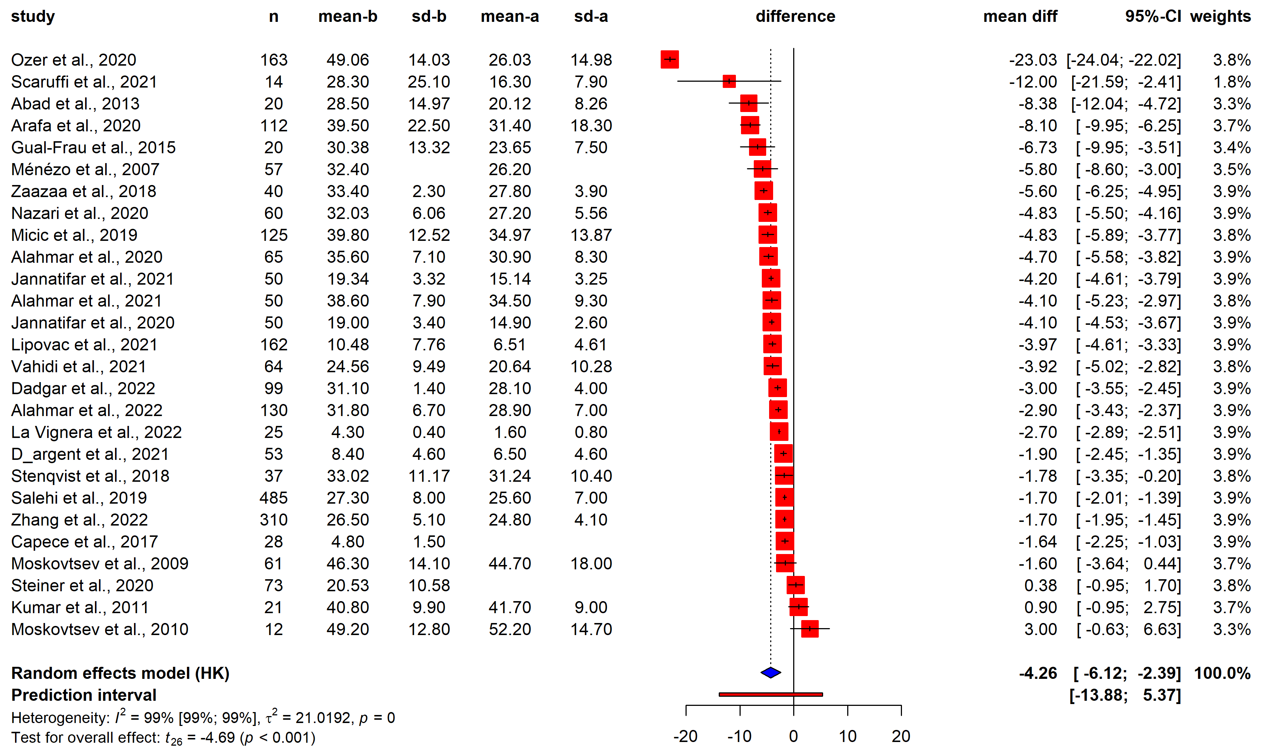
 **Supplementary Figure 83:** Comparison of patients’ mean sperm DNA fragmentation values with a 95% confidence interval before and 3 months after antioxidant supplementation with an input correlation of 0.9

**
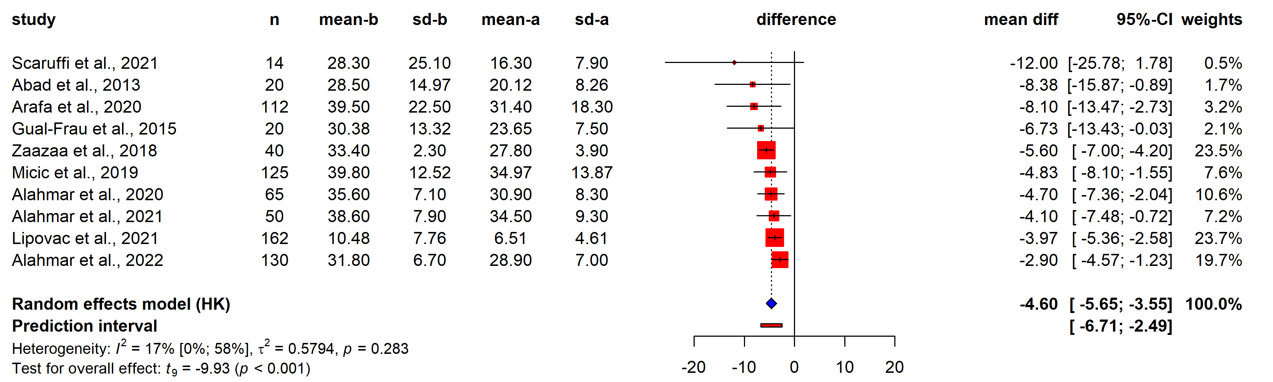
 Supplementary Figure 84:** Comparison of patients’ mean sperm DNA fragmentation values with a 95% confidence interval measured via SCD before and 3 months after antioxidant supplementation with an input correlation of 0

**
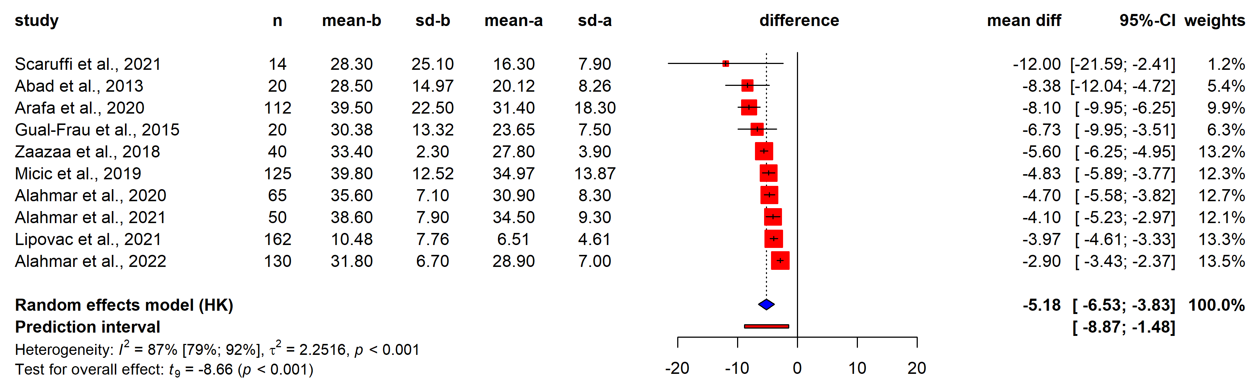
 Supplementary Figure 85:** Comparison of patients’ mean sperm DNA fragmentation values with a 95% confidence interval measured via SCD before and 3 months after antioxidant supplementation with an input correlation of 0.9

**
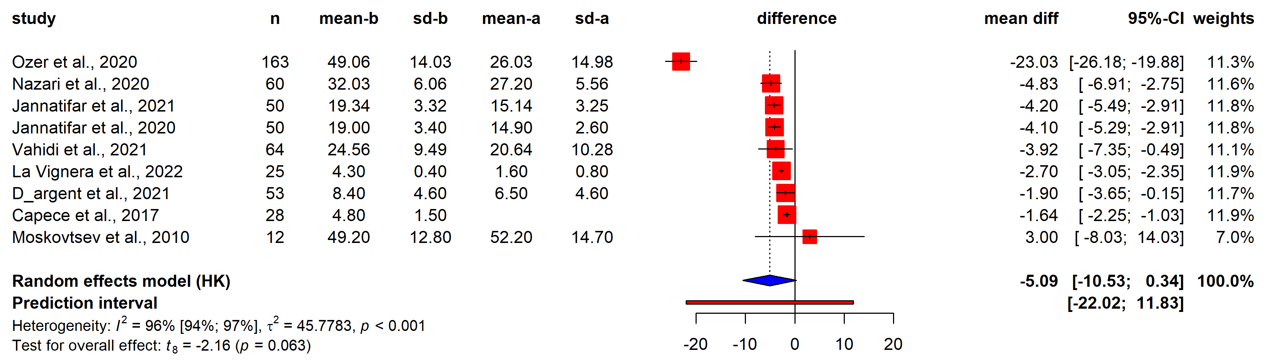
 Supplementary Figure 86:** Comparison of patients’ mean sperm DNA fragmentation values with a 95% confidence interval measured via TUNEL assay before and 3 months after antioxidant supplementation with an input correlation of 0

**
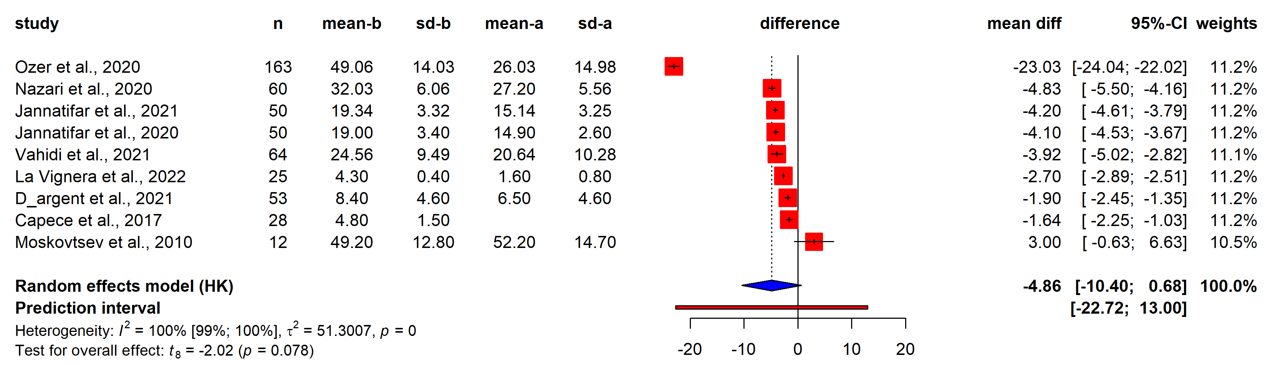
 Supplementary Figure 87:** Comparison of patients’ mean sperm DNA fragmentation values with a 95% confidence interval measured via TUNEL assay before and 3 months after antioxidant supplementation with an input correlation of 0.9


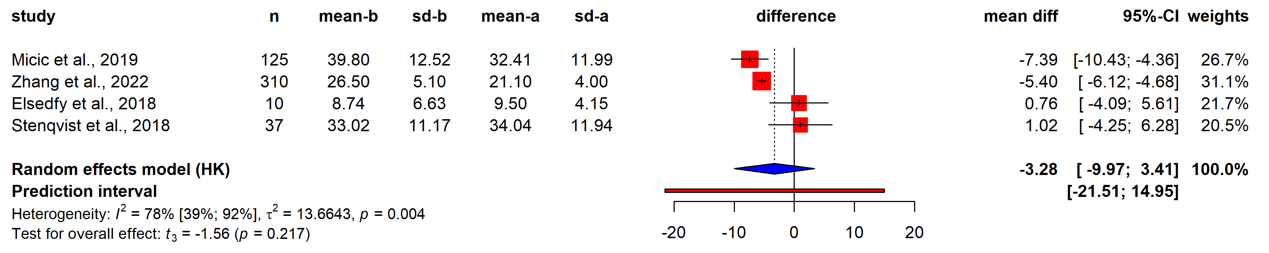
 **Supplementary Figure 88:** Comparison of patients’ mean sperm DNA fragmentation values with a 95% confidence interval before and 6 months after combined antioxidant supplementation with an input correlation of 0


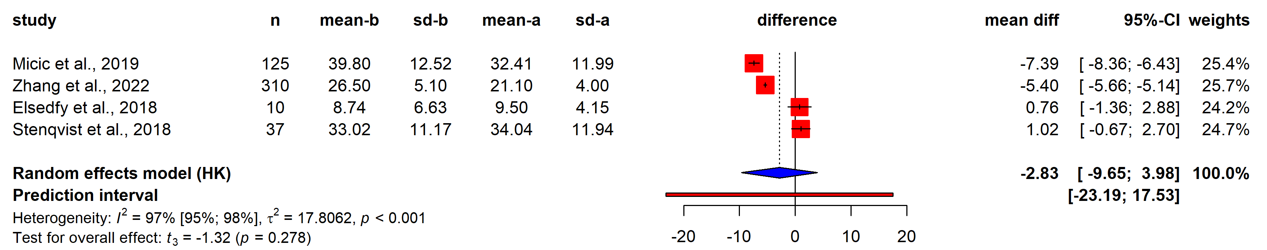
 **Supplementary Figure 89:** Comparison of patients’ mean sperm DNA fragmentation values with a 95% confidence interval before and 6 months after combined antioxidant supplementation with an input correlation of 0.9

**
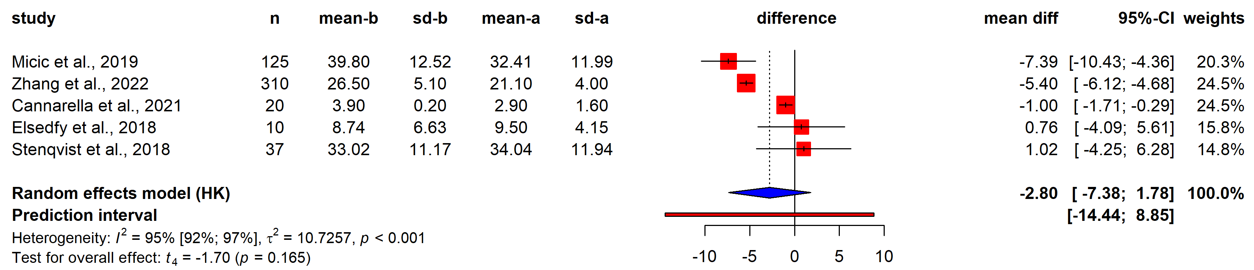
 Supplementary Figure 90:** Comparison of patients’ mean sperm DNA fragmentation values with a 95% confidence interval before and 6 months after antioxidant supplementation with an input correlation of 0

**Supplementary Figure 91:** Comparison of patients’ mean sperm DNA fragmentation values with a 95% confidence interval before and 6 months after antioxidant supplementation with an input correlation of 0.9

**Supplementary Figure 92:** Comparison of patients’ mean sperm DNA fragmentation values with a 95% confidence interval measured via SCSA before and 6 months after antioxidant supplementation with an input correlation of 0

**Supplementary Figure 93:** Comparison of patients’ mean sperm DNA fragmentation values with a 95% confidence interval measured via SCSA before and 6 months after antioxidant supplementation with an input correlation of 0.9

**Supplementary Figure 94:** Comparison of mean sperm DNA fragmentation values with a 95% confidence interval of patients with no comorbidities before and 6 months after antioxidant supplementation with an input correlation of 0

**Supplementary Figure 95:** Comparison of mean sperm DNA fragmentation values with a 95% confidence interval of patients with no comorbidities before and 6 months after antioxidant supplementation with an input correlation of 0.9

**Follicle stimulating hormone (FSH) treatment:**

**Supplementary Figure 96:** Comparison of patients’ mean sperm DNA fragmentation values with a 95% confidence interval before and 3 months after FSH treatment with an input correlation of 0

**Supplementary Figure 97:** Comparison of patients’ mean sperm DNA fragmentation values with a 95% confidence interval before and 3 months after FSH treatment with an input correlation of 0.8

**Supplementary Figure 98: C**omparison of patients’ mean sperm DNA fragmentation values with a 95% confidence interval measured via TUNEL assay before and 3 months after FSH treatment with an input correlation of 0

**Supplementary Figure 99: C**omparison of patients’ mean sperm DNA fragmentation values with a 95% confidence interval measured via TUNEL assay before and 3 months after FSH treatment with an input correlation of 0.8

**Supplementary Figure 100:** Comparison of patients’ mean sperm DNA fragmentation values with a 95% confidence interval before and 3 months after 150 IU dosages of FSH treatment every other day with an input correlation of 0

**Supplementary Figure 101:** Comparison of patients’ mean sperm DNA fragmentation values with a 95% confidence interval before and 3 months after 150 IU dosages of FSH treatment every other day with an input correlation of 0.8

**Supplementary Figure 102:** Comparison of patients’ mean sperm DNA fragmentation values with a 95% confidence interval measured via TUNEL assay before and 3 months after 150 IU dosages of FSH treatment every other day with an input correlation of 0

**Supplementary Figure 103:** Comparison of patients’ mean sperm DNA fragmentation values with a 95% confidence interval measured via TUNEL assay before and 3 months after 150 IU dosages of FSH treatment every other day with an input correlation of 0.8

**Lifestyle interventions:**

**Supplementary Figure 104:** Comparison of patients’ mean sperm DNA fragmentation values with a 95% confidence interval before and 3 months after lifestyle interventions with an input correlation of 0

**Supplementary Figure 105:** Comparison of patients’ mean sperm DNA fragmentation values with a 95% confidence interval before and 3 months after lifestyle interventions with an input correlation of 0.9

**REFERENCE**

1. Abad C, Amengual MJ, Gosálvez J, et al. Effects of oral antioxidant treatment upon the dynamics of human sperm DNA fragmentation and subpopulations of sperm with highly degraded DNA. Article. *Andrologia*. 2013;45(3):211-216. doi:10.1111/and.12003

2. Abbasi B, Abbasi H, Niroumand H. Synbiotic (FamiLact) administration in idiopathic male infertility enhances sperm quality, DNA integrity, and chromatin status: A triple-blinded randomized clinical trial. *Int J Reprod Biomed*. Mar 2021;19(3):235-244. doi:10.18502/ijrm.v19i3.8571

3. Abdelaziz AS, Burham WA, Aboelsaad AY, Badran YA, Abdel-Maguid Ahmed AF. Impact of extensive laparoscopic venous disconnection on the recurrence rate and sperm DNA quality in infertile varicocele patients. Article. *Urology Annals*. 2015;7(4):482-487. doi:10.4103/0974-7796.157964

4. Abdelbaki SA, Sabry JH, Al-Adl AM, Sabry HH. The impact of coexisting sperm DNA fragmentation and seminal oxidative stress on the outcome of varicocelectomy in infertile patients: a prospective controlled study. Article. *Arab journal of urology*. 2017;15(2):131‐139. doi:10.1016/j.aju.2017.03.002

5. Afsin M, Otludil B, Dede O, Akkus M. An examination on composition of spermatozoa obtained from pre-operative and post-operative varicocele patients. Article. *Reproductive biology*. 2018;18(4):361-367. doi:10.1016/j.repbio.2018.10.005

6. Alahmar AT, Calogero AE, Sengupta P, Dutta S. Coenzyme Q10 improves sperm parameters, oxidative stress markers and sperm DNA fragmentation in infertile patients with idiopathic oligoasthenozoospermia. Article. *World Journal of Men's Health*. 2020;38doi:10.5534/WJMH.190145

7. Alahmar AT, Sengupta P, Dutta S, Calogero AE. Coenzyme Q10, oxidative stress markers, and sperm DNA damage in men with idiopathic oligoasthenoteratospermia. *Clin Exp Reprod Med*. Jun 2021;48(2):150-155. doi:10.5653/cerm.2020.04084

8. Alahmar AT, Singh R. Comparison of the effects of coenzyme Q10 and Centrum multivitamins on semen parameters, oxidative stress markers, and sperm DNA fragmentation in infertile men with idiopathic oligoasthenospermia. *Clin Exp Reprod Med*. Mar 2022;49(1):49-56. doi:10.5653/cerm.2021.04910

9. Alhathal N, San Gabriel M, Zini A. Beneficial effects of microsurgical varicocoelectomy on sperm maturation, DNA fragmentation, and nuclear sulfhydryl groups: A prospective trial. Article. *Andrology*. 2016;4(6):1204-1208. doi:10.1111/andr.12256

10. Arafa M, Agarwal A, Majzoub A, et al. Efficacy of Antioxidant Supplementation on Conventional and Advanced Sperm Function Tests in Patients with Idiopathic Male Infertility. *Antioxidants (Basel)*. Mar 6 2020;9(3)doi:10.3390/antiox9030219

11. Baker K, McGill J, Sharma R, Agarwal A, Sabanegh E, Jr. Pregnancy after varicocelectomy: impact of postoperative motility and DFI. *Urology*. Apr 2013;81(4):760-6. doi:10.1016/j.urology.2012.12.005

12. Boeri L, Lucignani G, Jannello LMI, et al. Clinically Meaningful Improvements in Sperm DNA Fragmentation Severity in Infertile Men Treated with Superoxide Dismutase Supplementation: A Single-Center Experience. *J Clin Med*. Nov 4 2022;11(21)doi:10.3390/jcm11216540

13. Cannarella R, Condorelli RA, Calogero AE, et al. Effects of selenium supplementation on sperm parameters and dna‐fragmentation rate in patients with chronic autoimmune thyroiditis. Article. *Journal of Clinical Medicine*. 2021;10(16)doi:10.3390/jcm10163755

14. Capece M, Romeo G, Ruffo A, Romis L, Mordente S, Di Lauro G. A phytotherapic approach to reduce sperm DNA fragmentation in patients with male infertility. Journal Article; Randomized Controlled Trial. *Urologia*. 2017;84(2):79‐82. doi:10.5301/uro.5000210

15. Colacurci N, De Leo V, Ruvolo G, et al. Recombinant FSH Improves Sperm DNA Damage in Male Infertility: A Phase II Clinical Trial. *Front Endocrinol (Lausanne)*. 2018;9:383. doi:10.3389/fendo.2018.00383

16. Colacurci N, Monti MG, Fornaro F, et al. Recombinant human FSH reduces sperm DNA fragmentation in men with idiopathic oligoasthenoteratozoospermia. Journal Article; Randomized Controlled Trial. *Journal of andrology*. 2012;33(4):588‐593. doi:10.2164/jandrol.111.013326

17. Condorelli RA, Cannarella R, Crafa A, et al. Retrospective monocentric clinical study on male infertility: Comparison between two different therapeutic schemes using follicle-stimulating hormone. Article. *Journal of Clinical Medicine*. 2021;10(12)doi:10.3390/jcm10122665

18. Mathieu d'Argent E, Ravel C, Rousseau A, et al. High-Dose Supplementation of Folic Acid in Infertile Men Improves IVF-ICSI Outcomes: A Randomized Controlled Trial (FOLFIV Trial). *J Clin Med*. Apr 26 2021;10(9)doi:10.3390/jcm10091876

19. Dadfar M, Kheradmand A, Mombeini H, Mohammadi Asl J, Mahdavian A. Evaluation of the Relationship Between Body Mass Index (BMI) and DNA Fragmentation Index Changes in Primary Infertile Patients Following Microscopic Sub Inguinal Varicocelectomy. Research Article. *Nephro-Urol Mon*. 2021;13(4):e115829. doi:10.5812/numonthly.115829

20. Dadgar Z, Shariatzadeh SMA, Mehranjani MS, Kheirolahi A. The therapeutic effect of co-administration of pentoxifylline and zinc in men with idiopathic infertility. *Ir J Med Sci*. Feb 2023;192(1):431-439. doi:10.1007/s11845-022-02931-0

21. Dhawan V, Kumar M, Deka D, et al. Meditation & yoga: Impact on oxidative DNA damage & dysregulated sperm transcripts in male partners of couples with recurrent pregnancy loss. Article. *Indian Journal of Medical Research*. 2018;148(7):134-139. doi:10.4103/ijmr.IJMR_1988_17

22. Elsedfy H, De Sanctis V, Ahmed AY, Mohamed NR, Arafa M, Elalfy MS. A pilot study on sperm DNA damage in β-thalassemia major: Is there a role for antioxidants? Article. *Acta Biomedica*. 2018;89(1):47-54. doi:10.23750/abm.v89i1.6836

23. Esfahani MHN, Abbasi H, Mirhosseini Z, et al. Can altered expression of HSPA2 in varicocele patients lead to abnormal spermatogenesis? Article. *International Journal of Fertility and Sterility*. 2010;4(3):104-113.

24. Fathi A, Mohamed O, Mahmoud O, et al. The impact of varicocelectomy on sperm DNA fragmentation and pregnancy rate in subfertile men with normal semen parameters: A pilot study. *Arab J Urol*. Feb 14 2021;19(2):186-190. doi:10.1080/2090598x.2021.1889746

25. Fornaro F, La Verde E, Zurzolo L, Lambiase A, Pacilio C, Colacurci N. Effect of the therapy with recombinant FSH on the fragmentation of the spermatic DNA in patients with idiopathic oligoasthenoteratozoospermia undergoing ICSI. Article. *Giornale Italiano di Ostetricia e Ginecologia*. 2009;31(6-7):308-310.

26. Gabriel MS, Chan SW, Alhathal N, Chen JZ, Zini A. Influence of microsurgical varicocelectomy on human sperm mitochondrial DNA copy number: A pilot study. Article. *Journal of Assisted Reproduction and Genetics*. 2012;29(8):759-764. doi:10.1007/s10815-012-9785-z

27. Gallegos G, Ramos B, Santiso R, Goyanes V, Gosálvez J, Fernández JL. Sperm DNA fragmentation in infertile men with genitourinary infection by Chlamydia trachomatis and Mycoplasma. Article. *Fertility and Sterility*. 2008;90(2):328-334. doi:10.1016/j.fertnstert.2007.06.035

28. Garolla A, Ghezzi M, Cosci I, et al. FSH treatment in infertile males candidate to assisted reproduction improved sperm DNA fragmentation and pregnancy rate. Article. *Endocrine*. 2017;56(2):416-425. doi:10.1007/s12020-016-1037-z

29. Ghazi S, Abdelfattah H. Effect of microsurgical varicocelectomy on sperm DNA fragmentation in infertile men. Journal: Article. *Middle east fertility society journal*. 2011;16(2):149‐153. doi:10.1016/j.mefs.2010.12.013

30. Greco E, Iacobelli M, Rienzi L, Ubaldi F, Ferrero S, Tesarik J. Reduction of the incidence of sperm DNA fragmentation by oral antioxidant treatment. Clinical Trial; Comparative Study; Journal Article; Randomized Controlled Trial. *Journal of andrology*. 2005;26(3):349‐353. doi:10.2164/jandrol.04146

31. Gual-Frau J, Abad C, Amengual MJ, et al. Oral antioxidant treatment partly improves integrity of human sperm DNA in infertile grade i varicocele patients. Article. *Human Fertility*. 2015;18(3):225-229. doi:10.3109/14647273.2015.1050462

32. Habibi M, Abbasi B, Fakhari Zavareh Z, et al. Alpha-Lipoic Acid Ameliorates Sperm DNA Damage and Chromatin Integrity in Men with High DNA Damage: A Triple Blind Randomized Clinical Trial. *Cell J*. Oct 1 2022;24(10):603-611. doi:10.22074/cellj.2022.8273

33. Hajizadeh Maleki B, Tartibian B. High-intensity interval training modulates male factor infertility through anti-inflammatory and antioxidative mechanisms in infertile men: a randomized controlled trial. Journal Article; Randomized Controlled Trial. *Cytokine*. 2020;125:154861. doi:10.1016/j.cyto.2019.154861

34. Huang WJ, Lu XL, Li JT, Zhang JM. Effects of folic acid on oligozoospermia with MTHFR polymorphisms in term of seminal parameters, DNA fragmentation, and live birth rate: a double-blind, randomized, placebo-controlled trial. Journal Article; Randomized Controlled Trial. *Andrology*. 2020;8(1):110‐116. doi:10.1111/andr.12652

35. Humaidan P, Haahr T, Povlsen BB, et al. The combined effect of lifestyle intervention and antioxidant therapy on sperm DNA fragmentation and seminal oxidative stress in IVF patients: a pilot study. Article in Press. *International braz j urol : official journal of the Brazilian Society of Urology*. 2021;47doi:10.1590/S1677-5538.IBJU.2021.0604

36. Jannatifar R, Parivar K, Hayati Roodbari N, Nasr-Esfahani MH. The Effect of N-Acetyl-Cysteine on NRF2 Antioxidant Gene Expression in Asthenoteratozoospermia Men: A Clinical Trial Study. *Int J Fertil Steril*. Oct 2020;14(3):171-175. doi:10.22074/ijfs.2020.44411

37. Jannatifar R, Cheraghi E, Nasr-Esfahani MH, Piroozmanesh H. Association of heat shock protein A2 expression and sperm quality after N-acetyl-cysteine supplementation in astheno-terato-zoospermic infertile men. *Andrologia*. Jun 2021;53(5):e14024. doi:10.1111/and.14024

38. Kavoussi P, Abdullah N, Gilkey M, et al. The impact of ipsilateral testicular atrophy on semen quality and sperm DNA fragmentation response to varicocele repair. Journal: Article. *Asian journal of andrology*. 2021;23(2):146‐149. doi:10.4103/aja.aja_50_20

39. Kavoussi PK, Gilkey MS, Machen GL, Kavoussi SK, Dorsey C. Varicocele Repair Improves Static Oxidation Reduction Potential as a Measure of Seminal Oxidative Stress Levels in Infertile Men: A Prospective Clinical Trial Using the MiOXSYS System. *Urology*. Jul 2022;165:193-197. doi:10.1016/j.urology.2022.04.007

40. Kooshesh L, Bahmanpour S, Zeighami S, Nasr-Esfahani MH. Effect of Letrozole on sperm parameters, chromatin status and ROS level in idiopathic Oligo/Astheno/Teratozoospermia. Article. *Reproductive Biology and Endocrinology*. 2020;18(1)doi:10.1186/s12958-020-00591-2

41. Kumar R, Saxena V, Shamsi MB, Venkatesh S, Dada R. Herbo-mineral supplementation in men with idiopathic oligoasthenoteratospermia : a double blind randomized placebo-controlled trial. Journal: Article. *Indian journal of urology*. 2011;27(3):357‐362. doi:10.4103/0970-1591.85440

42. La Vignera S, Condorelli R, Vicari E, D'Agata R, Calogero AE. Effects of varicocelectomy on sperm DNA fragmentation, mitochondrial function, chromatin condensation, and apoptosis. Article. *Journal of Andrology*. 2012;33(3):389-396. doi:10.2164/jandrol.111.013433

43. La Vignera S, Basile L, Aversa A, et al. The Use of Ellagic Acid and Annona Muricata Improves Semen Quality in Men with High-Risk Papillomavirus Infection. *J Clin Med*. Aug 11 2022;11(16)doi:10.3390/jcm11164691

44. Lara-Cerrillo S, Gual-Frau J, Benet J, et al. Microsurgical varicocelectomy effect on sperm telomere length, DNA fragmentation and seminal parameters. Article in Press. *Human Fertility*. 2020;doi:10.1080/14647273.2019.1711204

45. Li F, Yamaguchi K, Okada K, et al. Significant improvement of sperm DNA quality after microsurgical repair of varicocele. Article. *Systems Biology in Reproductive Medicine*. 2012;58(5):274-277. doi:10.3109/19396368.2012.692431

46. Lipovac M, Nairz V, Aschauer J, Riedl C. The effect of micronutrient supplementation on spermatozoa DNA integrity in subfertile men and subsequent pregnancy rate. Article. *Gynecological Endocrinology*. 2021;37(8):711-715. doi:10.1080/09513590.2021.1923688

47. Maghsoumi-Norouzabad L, Zare Javid A, Mansoori A, Dadfar M, Serajian A. Vitamin D3 Supplementation Effects on Spermatogram and Oxidative Stress Biomarkers in Asthenozoospermia Infertile Men: a Randomized, Triple-Blind, Placebo-Controlled Clinical Trial. *Reprod Sci*. Mar 2022;29(3):823-835. doi:10.1007/s43032-021-00769-y

48. Martínez-Soto JC, Domingo JC, Cordobilla B, et al. Dietary supplementation with docosahexaenoic acid (DHA) improves seminal antioxidant status and decreases sperm DNA fragmentation. Journal Article; Randomized Controlled Trial. *Systems biology in reproductive medicine*. 2016;62(6):387‐395. doi:10.1080/19396368.2016.1246623

49. Ménézo YJ, Hazout A, Panteix G, et al. Antioxidants to reduce sperm DNA fragmentation: an unexpected adverse effect. *Reprod Biomed Online*. Apr 2007;14(4):418-21. doi:10.1016/s1472-6483(10)60887-5

50. Micic S, Lalic N, Djordjevic D, et al. Double-blind, randomised, placebo-controlled trial on the effect of L-carnitine and L-acetylcarnitine on sperm parameters in men with idiopathic oligoasthenozoospermia. Journal Article; Randomized Controlled Trial. *Andrologia*. 2019;51(6):e13267. doi:10.1111/and.13267

51. Miñambres I, Sardà H, Urgell E, et al. Obesity Surgery Improves Hypogonadism and Sexual Function in Men without Effects in Sperm Quality. *J Clin Med*. Aug 31 2022;11(17)doi:10.3390/jcm11175126

52. Mir J, Franken D, Andrabi SW, Ashraf M, Rao K. Impact of weight loss on sperm DNA integrity in obese men. Article. *Andrologia*. 2018;50(4)doi:10.1111/and.12957

53. Mohammed EE, Mosad E, Zahran AM, Hameed DA, Taha EA, Mohamed MA. Acridine Orange and Flow Cytometry: Which Is Better to Measure the Effect of Varicocele on Sperm DNA Integrity? *Adv Urol*. 2015;2015:814150. doi:10.1155/2015/814150

54. Moskovtsev SI, Jarvi K, Mullen JB, Cadesky KI, Hannam T, Lo KC. Testicular spermatozoa have statistically significantly lower DNA damage compared with ejaculated spermatozoa in patients with unsuccessful oral antioxidant treatment. *Fertil Steril*. Mar 1 2010;93(4):1142-6. doi:10.1016/j.fertnstert.2008.11.005

55. Moskovtsev SI, Lecker I, Mullen JB, et al. Cause-specific treatment in patients with high sperm DNA damage resulted in significant DNA improvement. *Syst Biol Reprod Med*. Mar-Apr 2009;55(2):109-15. doi:10.1080/19396360902787944

56. Nazari A, Sabeti P, Pourmasumi S. Comparison between sperm parameters and chromatin in recurrent pregnancy loss couples after antioxidant therapy. *J Family Med Prim Care*. Feb 2020;9(2):597-601. doi:10.4103/jfmpc.jfmpc_1105_19

57. Negri L, Benaglia R, Monti E, Morenghi E, Pizzocaro A, Levi Setti PE. Effect of superoxide dismutase supplementation on sperm DNA fragmentation. Article. *Archivio Italiano di Urologia e Andrologia*. 2017;89(3):212-218. doi:10.4081/aiua.2017.3.212

58. Ni K, Steger K, Yang H, Wang H, Hu K, Chen B. Sperm protamine mRNA ratio and DNA fragmentation index represent reliable clinical biomarkers for men with varicocele after microsurgical varicocele ligation. Article. *The Journal of urology*. 2014;192(1):170-176. doi:10.1016/j.juro.2014.02.046

59. Ni K, Steger K, Yang H, et al. A comprehensive investigation of sperm DNA damage and oxidative stress injury in infertile patients with subclinical, normozoospermic, and astheno/oligozoospermic clinical varicocoele. Article. *Andrology*. 2016;4(5):816-824. doi:10.1111/andr.12210

60. Ozer C. Antioxidant treatment of increased sperm DNA fragmentation: Complex combinations are not more successful. Article. *Archivio Italiano di Urologia e Andrologia*. 2020;92(4):362-365. doi:10.4081/AIUA.2020.4.362

61. Özgök Kangal K, Özgök Y. Assisted reproductive treatments with hyperbaric oxygen therapy in male infertility. *Turk J Urol*. Mar 2021;47(2):98-105. doi:10.5152/tud.2020.20328

62. Palomba S, Falbo A, Espinola S, et al. Effects of highly purified follicle-stimulating hormone on sperm DNA damage in men with male idiopathic subfertility: a pilot study. Journal: Article. *Journal of endocrinological investigation*. 2011;34(10):747‐752. doi:10.3275/7745

63. Pelliccione F, Verratti V, D'Angeli A, et al. Physical exercise at high altitude is associated with a testicular dysfunction leading to reduced sperm concentration but healthy sperm quality. Article. *Fertility and Sterility*. 2011;96(1):28-33. doi:10.1016/j.fertnstert.2011.03.111

64. Pini T, Makloski R, Maruniak K, Schoolcraft WB, Katz-Jaffe MG. Mitigating the Effects of Oxidative Sperm DNA Damage. *Antioxidants (Basel)*. Jul 6 2020;9(7)doi:10.3390/antiox9070589

65. Sakamoto Y, Ishikawa T, Kondo Y, Yamaguchi K, Fujisawa M. The assessment of oxidative stress in infertile patients with varicocele. Article. *BJU International*. 2008;101(12):1547-1552. doi:10.1111/j.1464-410X.2008.07517.x

66. Salas-Huetos A, Moraleda R, Giardina S, et al. Effect of nut consumption on semen quality and functionality in healthy men consuming a Western-style diet: a randomized controlled trial. Journal Article; Randomized Controlled Trial; Research Support, Non‐U.S. Gov't. *American journal of clinical nutrition*. 2018;108(5):953‐962. doi:10.1093/ajcn/nqy181

67. Salehi P, Shahrokhi SZ, Kamran T, Ajami A, Taghiyar S, Deemeh MR. Effect of antioxidant therapy on the sperm DNA integrity improvement; a longitudinal cohort study. Article. *International Journal of Reproductive BioMedicine*. 2019;17(2):99-106. doi:10.18502/ijrm.v17i2.3987

68. Samavat J, Cantini G, Lotti F, et al. Massive Weight Loss Obtained by Bariatric Surgery Affects Semen Quality in Morbid Male Obesity: a Preliminary Prospective Double-Armed Study. Article. *Obesity Surgery*. 2018;28(1):69-76. doi:10.1007/s11695-017-2802-7

69. Scaruffi P, Licata E, Maccarini E, et al. Oral Antioxidant Treatment of Men Significantly Improves the Reproductive Outcome of IVF Cycles. *Journal of Clinical Medicine*. 2021;10(15). doi:10.3390/jcm10153254

70. Simoni M, Santi D, Negri L, et al. Treatment with human, recombinant FSH improves sperm DNA fragmentation in idiopathic infertile men depending on the FSH receptor polymorphism p.N680S: a pharmacogenetic study. Journal: Article. *Human reproduction (Oxford, England)*. 2016;31(9):1960‐1969. doi:10.1093/humrep/dew167

71. Smit M, Romijn JC, Wildhagen MF, Veldhoven JL, Weber RF, Dohle GR. Decreased sperm DNA fragmentation after surgical varicocelectomy is associated with increased pregnancy rate. *J Urol*. Jan 2013;189(1 Suppl):S146-50. doi:10.1016/j.juro.2012.11.024

72. Steiner AZ, Hansen KR, Barnhart KT, et al. The effect of antioxidants on male factor infertility: the Males, Antioxidants, and Infertility (MOXI) randomized clinical trial. Journal Article; Multicenter Study; Randomized Controlled Trial; Research Support, N.I.H., Extramural. *Fertility and sterility*. 2020;113(3):552‐560.e3. doi:10.1016/j.fertnstert.2019.11.008

73. Stenqvist A, Oleszczuk K, Leijonhufvud I, Giwercman A. Impact of antioxidant treatment on DNA fragmentation index: a double-blind placebo-controlled randomized trial. Journal Article; Randomized Controlled Trial; Research Support, Non‐U.S. Gov't. *Andrology*. 2018;6(6):811‐816. doi:10.1111/andr.12547

74. Sun XL, Wang JL, Peng YP, et al. Bilateral is superior to unilateral varicocelectomy in infertile males with left clinical and right subclinical varicocele: a prospective randomized controlled study. Journal Article; Randomized Controlled Trial. *International urology and nephrology*. 2018;50(2):205‐210. doi:10.1007/s11255-017-1749-x

75. Telli O, Sarici H, Kabar M, Ozgur BC, Resorlu B, Bozkurt S. Does varicocelectomy affect DNA fragmentation in infertile patients? Article. *Indian Journal of Urology*. 2015;31(2):116-119. doi:10.4103/0970-1591.152811

76. Vahidi S, Narimani N, Ghanizadeh T, et al. the Short Abstinence May Have Paradoxical Effects On Sperms With Different Level Of DNA Integrity: A Prospective Study. *Urol J*. Jul 26 2021;18(6):682-687. doi:10.22037/uj.v18i.6515

77. Verdi A, Sahraei SS, Asa E, Jannatifar R, Masaeimanesh MB. The effect of recombinant human follicle-stimulating hormone on sperm DNA fragmentation and sperm parameters in oligozoospermic infertile men. Article. *Research in Molecular Medicine*. 2020;8(2):55-62. doi:10.32598/rmm.8.2.1130.1

78. Wald G, Punjani N, Gaffney C, Goldstein M, Kashanian JA. Impact of testicular delivery and vasal vein ligation on clinical outcomes in men undergoing microsurgical varicocelectomy. *Int Urol Nephrol*. Dec 2021;53(12):2453-2458. doi:10.1007/s11255-021-03009-z

79. Wang Y, Zhang W, Li D. High ligation of varicocele improves sperm DNA integrity in patients with asthenospermia. Article. *Journal of Central South University (Medical Sciences)*. 2012;37(12):1228-1232. doi:10.3969/j.issn.l672-7347.2012.12.008

80. Werthman P, Wixon R, Kasperson K, Evenson DP. Significant decrease in sperm deoxyribonucleic acid fragmentation after varicocelectomy. Article. *Fertility and Sterility*. 2008;90(5):1800-1804. doi:10.1016/j.fertnstert.2006.09.019

81. Яковлев ИБ, Теплых С, Рыбалов М, et al. Влияние препарата Простатилен® АЦ на уровень фрагментации днк сперматозоидов и антиоксидантную активность спермоплазмы у пациентов с бесплодием. *Экспериментальная и клиническая фармакология*. 2022;85(2):11-15.

82. Yuan C, Song H, Wang Z, Wang H. Peijingsu effectively improves sperm DNA integrity. Article. *Zygote (Cambridge, England)*. 2021;29(4):260-263. doi:10.1017/S0967199420000738

83. Zaazaa A, Adel A, Fahmy I, Elkhiat Y, Awaad AA, Mostafa T. Effect of varicocelectomy and/or mast cells stabilizer on sperm DNA fragmentation in infertile patients with varicocele. Clinical Trial; Journal Article; Randomized Controlled Trial. *Andrology*. 2018;6(1):146‐150. doi:10.1111/andr.12445

84. Zhang HY, Mu Y, Chen P, et al. Metabolic enzyme gene polymorphisms predict the effects of antioxidant treatment on idiopathic male infertility. *Asian J Androl*. Jul-Aug 2022;24(4):430-435. doi:10.4103/aja202180

85. Zhao N, Lu XL, Li JT, Zhang JM. Treatment of idiopathic oligozoospermia with combined human chorionic gonadotropin/human menopausal gonadotrophin: a randomised, double-blinded, placebo-controlled clinical study. *Andrologia*. 2019:e13271. doi:10.1111/and.13271

86. Zini A, Azhar R, Baazeem A, Gabriel MS. Effect of microsurgical varicocelectomy on human sperm chromatin and DNA integrity: A prospective trial. Article. *International Journal of Andrology*. 2011;34(1):14-19. doi:10.1111/j.1365-2605.2009.01048.x
